# Fecal Microbiota Transplantation Relieves Gastrointestinal and Autism Symptoms by Improving the Gut Microbiota in an Open-Label Study

**DOI:** 10.3389/fcimb.2021.759435

**Published:** 2021-10-19

**Authors:** Ning Li, Hongyan Chen, Yi Cheng, Fenghua Xu, Guangcong Ruan, Senhong Ying, Wen Tang, Lu Chen, Minjia Chen, LinLing Lv, Yi Ping, Dongfeng Chen, Yanling Wei

**Affiliations:** ^1^Department of Gastroenterology, Daping Hospital, Army Medical University (Third Military Medical University), Chongqing, China; ^2^Department of Gastroenterology, North-Kuanren General Hospital, Chongqing, China

**Keywords:** gut microbiota, fecal microbiota transplantation, autism spectrum disorders, clinic trial, microbiome-gut-brain axis

## Abstract

Autism spectrum disorder (ASD) is a severe brain development disorder that is characterized by deficits in social communication and restricted, repetitive and stereotyped behaviors. Accumulating evidence has suggested that gut microbiota disorders play important roles in gastrointestinal symptoms and neurodevelopmental dysfunction in ASD patients. Manipulation of the gut microbiota by fecal microbiota transplantation (FMT) was recently shown to be a promising therapy for the treatment of various diseases. Here, we performed a clinical trial to evaluate the effect of FMT on gastrointestinal (GI) and ASD symptoms and gut microbiota alterations in children with ASD. We found that there was a large difference in baseline characteristics of behavior, GI symptoms, and gut microbiota between children with ASD and typically developing (TD) control children. FMT could improve GI symptoms and ASD symptoms without inducing any severe complications. Similarly, FMT significantly changed the serum levels of neurotransmitters. We further observed that FMT could promote the colonization of donor microbes and shift the bacterial community of children with ASD toward that of TD controls. The abundance of *Eubacterium coprostanoligenes* pre-FMT was positively correlated with high GSRS scores, whereas a decrease in *Eubacterium coprostanoligenes* abundance induced by FMT was associated with the FMT response. Our data suggest that FMT might be a promising therapeutic strategy to improve the GI and behavioral symptoms of patients with ASD, possibly due to its ability to alter gut microbiota and highlight a specific microbiota intervention that targets *Eubacterium coprostanoligenes* that can enhance the FMT response. This trial was registered at the Chinese Clinical Trial Registry (www.chictr.org.cn) (trial registration number ChiCTR1800014745).

## Introduction

Autism spectrum disorder (ASD) is a severe brain development disorder that is characterized by deficits in social communication and restricted, repetitive and stereotyped behaviors. Studies have shown that the morbidity of ASD is approximately 147/10,000 in Western countries, approximately 264/10,000 in Eastern countries, and 120/10,000 in China (Wan et al., 2013), with the male being affected more frequently than females. The pathogenesis of ASD is unclear and is currently thought to be related to genetic factors, immunomodulatory disorders, inflammation, and exposure to environmental toxins (Han et al., 2021).

Increasing reports have identified gut microbiota as an important regulator of brain development, function, and behavior, it is identified to be involved in ASD (Fung et al., 2017). Animal models have shown that transplantation of fecal microbiota from patients with neurological disorders would lead to typical disorder symptoms in germ-free mice (Davies et al., 2021), clinical evidence also indicated that interactions exist between gut microbiota and ASD behavior. A large number of studies have shown that patients with ASD have various gastrointestinal symptoms, such as distension, abdominal pain, diarrhea, and constipation, and that these symptoms are closely related to the severity of ASD (Vargason et al., 2019; Davies et al., 2021). Thus, a possible link between gut microbiota and ASD has been proposed. Moreover, changes in the composition of the gut microbiota and metabolites have been found in both ASD patients and animal models (Kushak et al., 2016; Bermudez-Martin et al., 2021). Meanwhile, toxins produced by abnormal gut microbiota can increase intestinal permeability and aggravate ASD symptoms, for example, *Fusobacterium* produces some nerve factors and exerts systemic effects. These findings suggest that gut microbiota disorders and metabolism play important roles in gastrointestinal symptoms and neurodevelopmental dysfunction in ASD patients.

The microbiota-gut-brain axis (MGBA) was recently proposed based on many studies. Through the MGBA, the gut microbiota and its metabolites can affect the body, and the body can regulate the gut microbiota composition through neural, immune, and endocrine pathways to adapt to environmental changes and maintain a microecological balance (Powell et al., 2017). An increasing number of studies have shown that deficits in the MGBA are one of the pathogenic mechanisms of ASD. Recent studies have shown that the gut microbiota is involved in the bidirectional regulation of the intestinal and central nervous systems through neural, endocrine, metabolic, and immune pathways (Osadchiy et al., 2019). It is believed that microecological therapies that improve the gut microbiota can alleviate some gastrointestinal symptoms and ASD symptoms in ASD patients, so far, some reports have already proved that gut microbiota intervention therapy is helpful for ASD improvement (Wang et al., 2020; Kong et al., 2021).

Fecal microbiota transplantation (FMT) refers to the transplantation of fecal microbiota from healthy donors to patients. It is a novel method that has been used in recent years to treat diseases such as infection, immune diseases, liver diseases, intestinal encephalopathy, and cancer (Bajaj et al., 2017; Routy et al., 2018; Wortelboer et al., 2019). Unlike probiotic therapy or others, FMT targets the entity of gut microbiota and serves as a safe and effective method for gut microecology reconstruction (Ianiro et al., 2018). Additionally, preliminary clinical studies and research on animal models have shown that FMT can significantly alleviate neurological disorders such as Parkinson’s disease and act as a protective treatment against neuroinflammation (Vendrik et al., 2020). However, whether FMT has a therapeutic effect on ASD and how it works to improve neurological symptoms are not thoroughly understood. Since there is a growing need to achieve measurable and long-term improvements in children with neuropsychiatric disorders, we hypothesize that FMT in ASD children might induce improvement in stereotyped symptoms with good safety. In this study, we conducted an open-label clinical trial to investigate the safety and efficacy of FMT for gastrointestinal and behavioral symptoms in children with ASD and explored the underlying mechanism of FMT-induced ASD improvement through the microbiome-gut-brain axis.

## Materials and Methods

### Study Design

This study was an open-label clinical trial involving 40 children with ASD (age 3-17 years) who were diagnosed by the Autism Diagnostic Interview-Revised (ADI-R) and accompanied with symptoms of the gastrointestinal tract (such as constipation, diarrhea, etc.), whose custodian can fully understand and accept the informed consent, all participants were able to undergo follow-up examination. Moreover, children with fever, have reliance on tube feeding, and accompanying emergent gastrointestinal disease that needs prompt medication, diagnosed with severe malnutrition or excessive weight loss or severe immunodeficiency disease, have a history of severe allergies, monogenic diseases, mental disorder or depression were exclude from the study. Besides, children who were undergoing probiotics or had antibiotics 7 days before screening were also excluded.

Apart from ASD children, 16 sex- and age-matched typically developing control (TD) children without gastrointestinal (GI) disorders were included in this trial as well. TD control children had no gastrointestinal symptoms in the last 1 month and had no antibiotics therapy for the last 3 months or any medication that would affect gut microbiota (such as proton pump inhibitors, gastrointestinal stimulants, steroids, and aspirin). Meanwhile, the Bristol stool score was 4 for each TD children.

**Figure 1:**
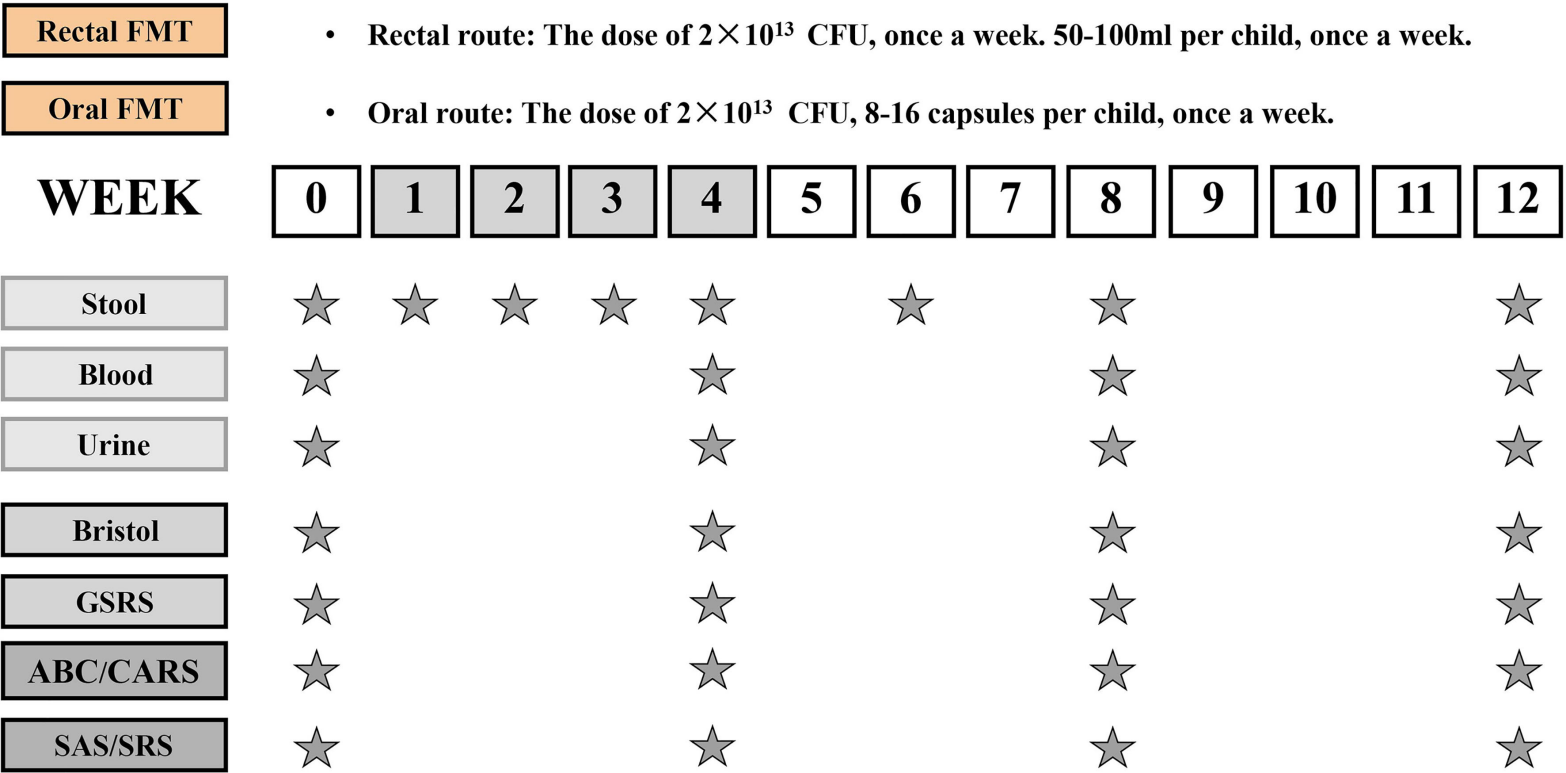
Study design timeline. The trial consisted of a 4-week period of FMT and an 8-week follow-up observation period after the end of treatment. The time schedule of sample collection and GI/behavioral assessments.

All children participated in the study for 12 weeks, which included a 4-week FMT treatment phase and an 8-week follow-up observation phase after the treatment. The TD children were monitored for 12 weeks without any intervention. There were two routes of administration for FMT, 27 children received oral route of FMT through freeze-dried capsules while 13 children received colonoscopic FMT. In this trial, participants who were not capable of swallowing capsules were chosen for colonoscopy, while the remaining participants could choose oral capsules, moreover, all participants were allowed to switch to another intervention if they had a strong preference regarding the route of administration. The researchers were not blinded to the group allocation or outcome assessment. **Figure 1** illustrates the study design. Neither vancomycin nor proton pump inhibitors (PPIs) was given before FMT. All participants received 2 liters of GOLYTELY (polyethylene glycol) before FMT and remained fasting until the scheduled treatment. To improve the tolerance of patients to GOLYTELY, we applied it by fractionated dose: 1 L was given one day before enema and the rest was given on the day of enema. For patients with poor tolerance (such as abdominal distention and vomiting), saline was added for bowel preparation. All the participants had clinic visits at weeks 0, 4, 8, and 12.

This clinical study was reviewed and approved by the Ethics Committee at the Army Medical Center of PLA. Written informed consent was obtained from each participant before enrollment. In addition, the study was registered at the Chinese Clinical Trial Registry (www.chictr.org.cn); the trial registration number was ChiCTR1800014745, through which the trial protocol can be accessed.

### Standardized Human Gut Microbiota and FMT Preparation

One rigorously screened donor volunteered to provided stool for all participants. The screening involved review of medical history, serological examinations to screen infectious disease, stool examination, gut microbiota sequencing, and confirmation of the absence of gastrointestinal disorders and other neurodevelopmental problems, meanwhile, *Helicobacter pylori* was also detected through C13 breath tests. The serological examination was performed to exclude hepatitis A, B, and C infections, human immunodeficiency virus-1 infection, human immunodeficiency virus-2 infection, TB infection of T cells, TORCH virus infection, and syphilis infection. Fasting glucose levels, lipid levels, liver function, renal function, and C-reactive protein levels were also assessed. The stool used for the preparation was tested for the presence of bacterial pathogens (*Escherichia coli* O157, *Shigella*, *Salmonella*, *Campylobacter*, *Staphylococcus aureus*, *Yersinia*, *Vibrio parahaemolyticus*, and *Vibrio cholera*), infection with viruses (rotavirus A, adenovirus, and norovirus), infection with fungi (*Candida albicans*) and the presence of parasites (Giardia, Cryptosporidium, Cyclospora, and Isospora).

The donated stool samples were collected under anaerobic and sterile conditions. The samples were mixed with sterile normal saline and then homogenized immediately. The homogenates were then filtered through 20 μm nylon filters to remove large particles and fibrous matter. The filtered suspensions were then centrifuged at 6000 g for 5 min at 4°C with a centrifuge (Sorvall SS-34). The supernatant was removed, and the precipitate was dissolved in normal saline, finally, a fecal bacterial solution of 60 mg/mL was obtained and used for future manufacture of FMT capsules or colonoscopic transplantation (10 g/50 kg per child). FMT capsules were prepared as previously reported (Xu et al., 2021), lyophilized protectant was added to the fecal bacterial solution and frozen at -80°C, which was then freeze-dried into powder using a cryogenic lyophilizer and placed in a capsule for further use.

The participants received 2 liters of GOLYTELY (polyethylene glycol) the night before transplantation. Both the oral capsule administration group and the rectal administration group received the same dose (approximately 2 × 10^14^ CFU per patient) once a week for 4 weeks.

### Evaluation and Sample Collection

During the study, the physical examination of participants was carried out at week 0. Blood samples were collected at week 0, 4, 8, and 12. Stool samples from the participants were collected at week 0, 1, 2, 3, 4, 6, 8, 10, and 12 for 16s rRNA sequencing to determine the composition and abundance of gut microbiota. For each participant, an interview by phone was conducted after FMT treatment. If the patients had any questions or adverse symptoms after FMT, more interviews or consultations would be carried out.

### Assessments of Gastrointestinal Symptoms

To evaluated the gastrointestinal symptoms of each participant, their parents were asked to complete the Gastrointestinal Symptoms Rating Scale (GSRS) and the Bristol Stool Form Scale at week 0, 4, 8, and 12. The GSRS is a self-management questionnaire that is a 7-point Likert scale with the following descriptive indicators: 1 (asymptomatic), 2 (slight), 3 (mild), 4 (moderate), 5 (moderate to severe), 6 (severe), and 7 (very severe). It was originally a hierarchical scale used for face-to-face questioning, mainly to evaluate common gastrointestinal symptoms, and was modified to generate a self-management questionnaire. The 15 items can be divided into 5 dimensions: abdominal pain (3 items), reflux (2 items), dyspepsia (4 items), diarrhea (3 items), and constipation (3 items). The questions were all regarding the severity of symptoms in the last 2 weeks. The score for each dimension was expressed as the average score of all items in the dimension, with the lowest score being 1 and the highest score being 7. The Bristol Stool Form Scale was used to assess the condition of the participant’s recent stool.

### Assessments of Autism and Related Symptoms

Autism and ASD related symptoms were assessed by the Childhood Autism Rating Scale (CARS), Social Responsiveness Scale (SRS) and Autism Behavior Checklist (ABC). The CARS is a 15-item scale that can be used to both diagnose ASD and assess the overall severity of the symptoms. The ABC assesses problem behaviors in five areas that are common in children with ASD, including irritability, lethargy, stereotypy, hyperactivity, and inappropriate speech. Additionally, we assessed parents’ levels of anxiety with the Self-Rating Anxiety Scale (SAS). The SAS is a 20-item scale that measures the severity of anxiety in parents of children with ASD. The CARS, ABC, SRS, and SAS were administered at baseline (week 0), at the end of treatment (week 4), and at the end of the observation period (week 12).

### Microbial DNA Extraction and 16S rRNA Sequencing

We collected fecal samples, stored them in a refrigerator at -80°C, and then delivered them to the G-Bio Sequencing Company (www.igeneseq.com) in dry ice, for further 16S rRNA sequencing. Microbial DNA was extracted from the fecal samples from ASD children, TD controls, and donors by using the PowerSoil DNA Isolation Kit (Mobio Carlsbad, CA). The purified PCR products were used to prepare a sequencing library using the TruSeq DNA Kit from Illumina following the manufacturer’s manual and were subjected to gene sequencing using Reagent Kit v3 on the MiSeq sequencer (Illumina, San Diego, CA, USA). The Quantitative Insights into Microbial Ecology 2 (QIIME2, version 2019.7) platform within a condo environment was used to process the sequencing data on our Linux server. Bioinformatics analysis was performed according to the official “Moving Pictures” tutorial provided by the QIIME2 website (https://docs.qiime2.org/2019.7/tutorials). Linear discriminant analysis effect size (LEfSe) was used to select significantly enriched candidates at the genus level. We compared the relative abundance of taxa between the two groups and within each group at different periods using the nonparametric Mann-Whitney U test followed by linear discriminant analysis (LDA) to estimate the effect size of each microbial feature with differential abundance. A taxon was considered significantly enriched if it had an LDA score greater than 2.0 and p value < 0.05.

### Serum 5-HT, GABA, and DA Measurements

Blood samples were taken from ASD children on week 0, 4, 8, and 12 in vacutainer tubes without anticoagulant, and were centrifuged at 3000 r/min for 10 min at room temperature. After centrifugation, the serum (supernatant) was transferred to clean tubes and was immediately stored at -80°C for future experiments. Each serum sample was kept at 4°C for 12 hours for thawing before use. In this study, ELISA kits (Thermo Fisher Scientific, USA) were used to quantify the serum concentration of 5-hydroxytryptamine (5-HT), dopamine (DA), and γ-aminobutyric acid (GABA) of each ASD child, based on the manufacturer’s instruction.

### Statistical Methods

For statistical analysis, SPSS 22.0 statistical software (SPSS Inc., Chicago, IL, USA) was used in this study. Measurement data are validated by a normality test and presented as mean ± SD. Comparisons of the measurement data were performed by t-test, Mann-Whitney U test, and one-way ANOVA (analysis of variance), and comparisons of categorical data were performed by chi-square test. Unless otherwise indicated, P < 0.05 was considered statistically significant.

## Results

### Characteristics of ASD and TD Subjects

Forty children who were diagnosed with ASD based on critical inclusion criteria, and 16 typically developing (TD) children from different families were enrolled in this study. All children with ASD received FMT treatment for consecutive 4 weeks and then underwent 8 weeks of follow-up study (the TD children were not treated during the whole trial) (**Figure 1**). The TD children had no relatives with any mental disorders. All participants enrolled in this trial were comparable in most demographic characteristics at baseline (**Table 1**), including ages, gender distributions, body mass indexes (BMIs). We also evaluated the consumption of the nutrients among participants, the consumption of carbohydrates and fat was comparable between children with ASD and TD cohort, while protein consumption was higher in ASD children (51.95 ± 9.84 g) than TD controls (48.44 ± 11.36 g) (p < 0.05). Moreover, children in the ASD cohort took more rounds of antibiotics during the first 3 years of life (5.75 ± 1.86 *vs* 10.35 ± 3.36, p<0.001), and suffer from a higher rate of food allergy incidence (6.25% *vs* 60.00%, p < 0.001).

**Table 1:** Demographic characteristics of study participants and their medical history.

Category	TD controls	ASD children	P value
	(n = 16)	(n = 40)	
Gender			
Female (n)	1	3	
Male (n)	15	37	
Age range	3-15	3-17	
2-3 years old (n)	1	2	
4-6 years old (n)	6	17	
7-11 years old (n)	7	14	
12-17 years old (n)	2	7	
Age (years), mean (SD)	7.13 ± 3.20	8.03 ± 3.73	0.564
BMI, (mean ± SD)	16.90 ± 2.52	17.96 ± 4.05	0.446
Autism severity, n (%)			
Mild		14 (35.00)	
Moderate		13 (32.50)	
Severe		13 (32.50)	
Food allergy (%)	6.25	60.00	P < 0.001
Oral antibiotic use during first 3 years of life (total rounds)	5.75 ± 1.86	10.35 ± 3.36	P < 0.001
Carbohydrate consumption (g)	106.56 ± 17.20	115.95 ± 16.20	0.059
Fat consumption (g)	54.06 ± 14.05	56.80 ± 15.50	0.543
Protein consumption (g)	48.44 ± 11.36	51.95 ± 9.84	P < 0.05

All values are mean ± standard deviation (SD). P value is calculated by one-way ANOVA analysis.

### Fecal Microbiota Transplantation Improves GI Symptoms and ASD Symptoms

**Figure 2:**
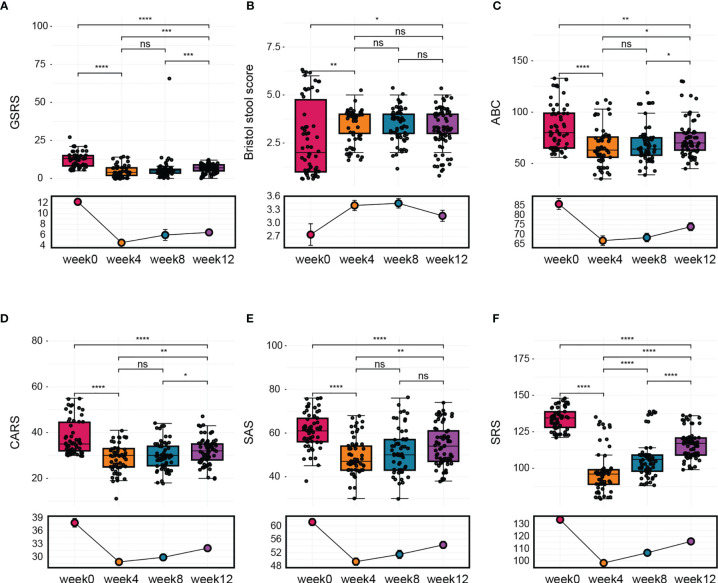
The change in GI symptoms and ASD symptoms after FMT. Children were treated with FMT for 4 weeks and underwent follow-up evaluation for 8 weeks after treatment ended. **(A)** Changes in average GSRS scores after FMT. **(B)** Bristol stool scores. **(C)** Results of ABC assessment at different time points. **(D)** CARS scores before treatment, after treatment, and 8 weeks after treatment. **(E)** Total SAS scores before treatment, after treatment, and 8 weeks after treatment. **(F)** Total SRS scores before treatment, after treatment, and 8 weeks after treatment. The two-tailed Wilcoxon signed-rank test was used to determine the significance. *P < 0.05, **P < 0.01, ***P < 0.001, ****P < 0.0001, ns, no significance.

During the follow-up phase, we found that children after FMT showed obvious improvement on symptoms like abdominal pain, constipation, or diarrhea, some children relived a lot from reflux as well. The overall GI symptoms of participants after treatment were measured using the GSRS. The average GSRS scores of ASD children decreased 35% after 4 weeks of FMT treatment and last for the next 8 weeks (**Figure 2A**), indicating that symptoms including abdominal pain, reflux, indigestion, diarrhea, and constipation were significantly improved after FMT. The Bristol Stool Form Scale and Daily Stool Record (DSR) were used to evaluate changes in stool properties. The occurrence of no stool, hard stool (type 1 or 2), and soft/liquid stool (type 6 or 7) was significantly decreased at the end of treatment compared to baseline, and this improvement persisted 8 weeks after FMT (**Figure 2B** and **Table 2**).

**Table 2:** The percent of stool style of ASD is based on the daily stool record and the Bristol Stool Form Scale (p valve by two-tailed χ^2^ test).

Category	Week 0	Week 4	Week 12	P value (Week 0 *vs.*)	
	(%)	(%)	(%)		
				Week 4	Week 12
No stool	0	0	0	NA	NA
Hard stool	60	17.5	10	P < 0.001	P < 0.001
(Type 1 or 2)					
Soft/liquid stool	10	0	0	0.040	0.040
(Type 6 or 7)					
Abnormal stool	70	17.5	10	P < 0.001	P < 0.001
(Hard/soft/liquid stool or no stool)					

NA, not applicable.

In addition to GI symptoms, ASD symptoms were also improved after FMT treatment. Scores on the ABC, which includes 57 items that assess mood, behavior, emotion, and language, were significantly alleviated by the treatment, and no obvious reversion was observed during 8 weeks after FMT (**Figure 2C**). Scores on the CARS, which evaluates core ASD symptoms, were decreased by 10% at the end of the treatment and remained decreased by 6% after 8 weeks (**Figure 2D**). The SAS was used to assess the level of anxiety in the parents of children with ASD. We found that parents’ anxiety levels decreased with the improvement of gastrointestinal symptoms and autism-like symptoms in the children with autism but returned to baseline levels by 8 weeks after the end of treatment (**Figure 2E**). Furthermore, children with ASD exhibited improvements in scores on the SRS, which assesses social skill deficits, at the end of treatment, but these improvements were reversed after 8 to 12 weeks without further treatment (**Figure 2F**).

Moreover, FMT was generally safe and only induced minimal adverse effects, including hyperactivity and aggression. Nausea/vomiting, major changes in blood chemistry, or long-term adverse effects were observed during follow-up (**Table 3**), suggesting that FMT was well tolerated. Thus, our data implied that FMT could improve GI symptoms and ASD symptoms without eliciting any severe complications and that the beneficial effects were gradually lost within a few weeks of the end of therapy, suggesting that extended treatment with FMT is needed.

**Table 3:** Adverse effects of oral and rectal administration.

Adverse effect	Incidence (%)	
	Oral	Rectal
Rash	0	0
Fever	3.7	0
Hyperactivity	3.7	7.7
Tantrums/aggression	3.7	0
Nausea/vomiting	0	0

### Oral and Rectal Route of FMT Induced Similar Effect on ASD Children

In this trial, FMT treatment was performed through oral or rectal (colonoscopic) route based on the condition of participants. To understand whether the administration route would lead to a different outcome on FMT recipients, we also analyzed the clinical indicators of oral and rectal FMT subgroups (**Supplementary Figure 1**).

Participants in the rectal FMT subgroup had a higher Bristol stool score at week 0, showing that children in the rectal subgroup tended to be suffered from diarrhea while the oral subgroup suffered more from constipation. At week 4, 8 and 12, the Bristol score of the two subgroups was also statistically different, interestingly, the discrepancy between the oral and the rectal subgroup decreased after 4 weeks of FMT treatment, and the score of the two subgroups get closer to 3 to 4 after FMT simultaneously, indicating that both oral and rectal FMT improved stool characteristics in ASD children. As for GSRS, the score of the rectal subgroup was significantly higher than the oral subgroup at week 0, this is consistent with the fact that children in the rectal subgroup had more severe symptoms and were not capable of swallowing capsules, however, after 4 weeks of FMT treatment, GSRS score of oral and rectal subgroup both decreased remarkably and showed no significance in comparison, this effect continued during the whole follow-up phase after FMT. This result implies that both oral and rectal routes of FMT could induce significant improvement in GI symptoms, and no significant difference was observed between oral or rectal FMT administration.

Consistent with GI symptoms, autism in participants in both oral and rectal subgroups showed similar change after FMT. At week 0, children in the rectal subgroup had a higher score of ABC and CARS, showing the severity of autism in rectal children, while children in the oral subgroup had a higher SRS score. After 4 consecutive weeks of FMT treatment, ABC, CARS, and SRS score of children in both subgroups decreased significantly and had no statistical difference at week 4, which sustained to week 8 and 12.

### FMT Alters the Serum Levels of 5-HT, DA, and GABA in Children With ASD

**Figure 3:**
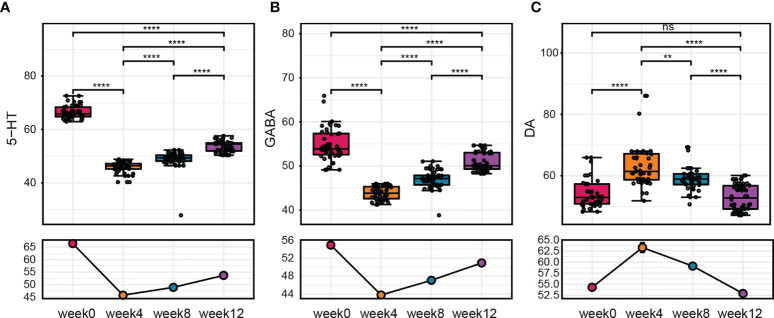
The change in neurotransmitter levels after FMT. **(A–C)** Changes in 5-HT, GABA, and DA levels at before treatment, after treatment, and 8 weeks after treatment. The Wilcoxon signed-rank test was used to determine the significance. **P < 0.01, ****P < 0.0001, ns, no significance.

Neurotransmitters are important in neurological regulation and can affect mood, cognition, and behavior in human beings, abnormality of neurotransmitters would lead to autistic behavior and other neurodevelopmental disorders, alteration of several neurotransmitters are also found in serum or plasma samples of ASD children (Marotta et al., 2020). To further evaluated the effect of FMT on ASD children, we next measured the serum levels of neurotransmitters after FMT. We found that 5-HT and GABA concentrations in the serum decreased after treatment, while the level of DA increased after 4 weeks of FMT (**Figure 3**). Similar to the GI symptom scores, the change of these neurotransmitters in serum was maintained weeks after FMT. During the whole follow-up, the level of 5-HT, DA and GABA changed most significantly at week 4 when FMT treatment was just completed and did not go further since then. Moreover, correlation analysis between the serum level of neurotransmitters and scales for clinical outcomes reveal that 5-HT had a negative correlation with Bristol stool score (r = -0.434, p = 0.001) while GABA had a positive correlation with Bristol score (r = 0.527, p < 0.001) (**Supplementary Table 1**), these results suggested that alteration of serum neurotransmitters could have a therapeutic outcome on clinical symptoms on ASD children. Furthermore, at week 8 and 12, these neurotransmitters were still at an altered level significantly but tend to get closer to the original level (**Figure 3**).

### Gut Microbiota Changes After FMT

We first evaluated alpha diversity and found that there were no significant differences in alpha diversity between TD and ASD groups at baseline (**Supplementary Figure 2**). To examine the differences in the microbiota between the two groups, we calculated beta diversity and found significant differences in beta diversity between the two groups (**Supplementary Figure 3**). At both the phylum and genus levels, the microbiota composition of patients with ASD differed from that of the TD controls (**Supplementary Figure 4**). To further identify the bacterial taxa with differential abundance between the ASD group and TD controls at baseline, we performed LEfSe analysis and identified eight genera with significant difference, we found that the abundance of gut microbiota in ASD children differs from TD controls, children in ASD group had a relative higher abundance of *Christensenellaceae*, *Akkermansiaceae* in family level, and *Christensenellaceae R 7 group*, *Akkermansia*, *Coprococcus 2*, *Eisenbergiella*, and *Tyzzerella 3* in genus level, while TD cohorts had a higher abundance of *Peptostreptococcaceae* in family level, and *Romboutsia*, *Fusicatenibacter*, *Eubacterium eligens group* in genus level (**Supplementary Figure 5**).

**Figure 4:**
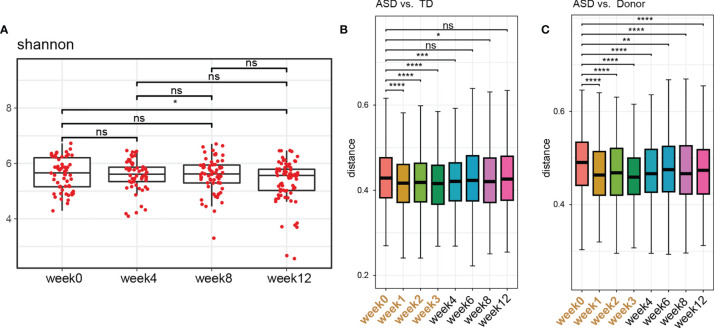
Gut microbiota changes after FMT. **(A)** Changes in alpha diversity were determined using Shannon indices in stool samples from children with ASD collected at different time points. The two-tailed Wilcoxon signed-rank test was used to analyze the difference. *P < 0.05. ns, not significant. **(B)** Unweighted UniFrac distance between ASD children treated with FMT at each timepoint and TD controls. **(C)** Unweighted UniFrac distance between ASD children treated with FMT at each timepoint and donors. The two-tailed Wilcoxon signed-rank test was used to analyze the difference. *P < 0.05, **P < 0.01, ***P < 0.0001. ****P < 0.00001. NS, not significant.

After FMT treatment for 4 weeks, although the gut microbiota diversity in ASD children remained unaltered, suggesting that FMT did not affect the overall structure of gut microbial communities (**Figure 4A**), the unweighted UniFrac distances of the recipient patient samples were significantly decreased compared with those of the TD controls and donor after 4 weeks of FMT (**Figures 4B, C**). Eight weeks after the FMT treatment (week 12), the distances increased to the level observed before FMT (**Figures 4B, C**), suggesting that FMT could promote the colonization of donor microbes and shift the bacterial community of patients with ASD toward that of TD controls or donor.

### Association of the Specific Gut Microbiota With the FMT Response

**Figure 5:**
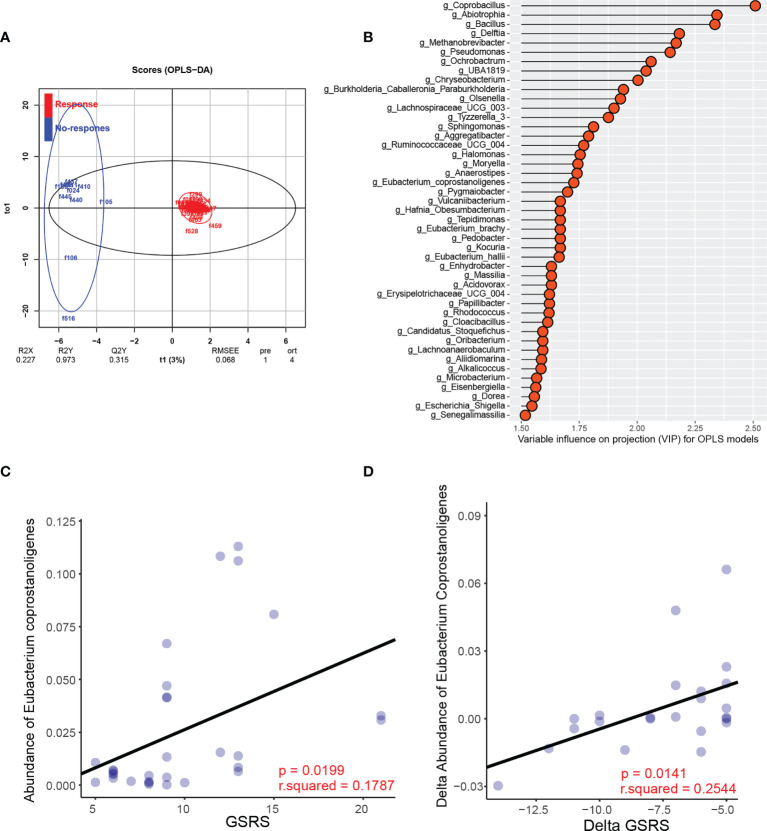
Specific microbial changes are associated with the response to FMT. **(A)** OPLS-DA analysis of the responder group and nonresponder group pre-FMT. **(B)** Variable influence on projection (VIP) for OPLS models. **(C)** Correlation between the abundance of *Eubacterium* and GSRS scores post-FMT in the FMT recipients. **(D)** Correlation between the change in the relative abundance of *Eubacterium* after FMT and the change in GSRS scores after FMT.

Because the pre-existing intestinal landscape can dramatically affect the response to FMT (Ericsson et al., 2017; Zuo et al., 2018), we next assessed the composition of the gut microbiota pre-FMT and analyzed its correlations with clinical outcomes post-FMT therapy. Children with ASD who achieved a less than 50% reduction in average GSRS score were defined as nonresponders. We found that FMT responders and nonresponders clustered separately based on orthogonal partial least squares discriminant analysis (OPLS-DA) (**Figure 5A**). The variable importance in projection (VIP) score for the gut microbiota showed that several genera contributed significantly to group separation (**Figure 5B**). Further comparisons of the relative abundance of all the significant bacteria between responders and nonresponders were performed. A significantly lower relative abundance of *Eubacterium coprostanoligenes* was reported in the responder group than in the nonresponder group (**Supplementary Figure 6**), whereas no significant differences in the abundance of the other bacterial genera were found (**Supplementary Figure 6**).

Furthermore, we observed a significant negative correlation between the pre-FMT relative abundance of *Eubacterium coprostanoligenes* and GSRS scores post-FMT in the FMT recipients (**Figure 5C**). We further assessed the changes in *Eubacterium coprostanoligenes* abundance after FMT treatment and found that the reduction in *Eubacterium coprostanoligenes* abundance was positively correlated with a reduction in GI symptoms, as indicated by the change in GSRS score (**Figure 5D**). Besides, the correlation analysis of neurotransmitters between gut microbiota showed that *Eubacterium coprostanoligenes* had a negative correlation at week 12 (r = 0.341, p = 0.048) with serum concentration of GABA. This result indicated that FMT might act partially to treat ASD by reducing the abundance of *Eubacterium coprostanoligenes* and that the genus *Eubacterium coprostanoligenes* is a potential regulator of FMT treatment response in children with ASD.

## Discussion

In this trial, to better understand how the gut microbiota works in ASD through the microbiome-gut-brain axis, we performed FMT by oral and rectal routes in ASD children who have associated gastrointestinal disorders and analyzed the gut microbiota and serum levels of several neurotransmitters. As our data suggested, ASD children with gastrointestinal symptoms suffered from gut dysbiosis, and FMT could serve as a protective treatment for reconstructing gut microbiota at both the phylum and genus levels and had a therapeutic outcome on autism symptoms and on gastrointestinal disorders. In addition, FMT also induced a desirable effect on recovering the serum levels of 5-HT, GABA, and DA in the ASD cohort, indicating that FMT might play an important role in modulating neurotransmitters through the MGB axis.

ASDs are complex neurobiological disorders that impair social interactions and communication and represent one of the pervasive developmental disorders. According to a recent report, the prevalence of ASD is 0.6% to 1.7% in children and adolescents, posing a heavy burden on public health (Iglesias-Vazquez et al., 2020). In ASD patients, symptoms are heterogeneous from one individual to another, and their disorders could be identified by two main features, regardless of sex, race, ethnicity, or other factors: social communication and restricted, repetitive sensory-motor behaviors (Lord et al., 2018). It is generally believed that various hazardous factors are associated with the onset of ASD, including both genetic and environmental factors, such as nutritional deficiencies or overloads, exposure to viruses, errors during embryonic neural tube closure, dysfunctional immune systems, and allergies (Bhandari et al., 2020). The genetic factors related to ASD are complex, and more than 100 genes and genomic regions have been reported and identified to be involved in the etiology of ASD (Li et al., 2017).

Apart from the main disorders, ASD patients often suffer from comorbidities, such as intellectual disability and gastrointestinal symptoms, and gastrointestinal disorders are reported to be highly prevalent. Many subjects with ASDs have significant gastrointestinal dysfunctions, including altered bowel habits and chronic abdominal pain, that accompany their neurological alterations (Madra et al., 2020). The incidence of constipation, diarrhea, abdominal pain, vomiting, and flatulence ranges from 9% to 90% in patients with ASD (Vuong and Hsiao, 2017). Gastroenterology disorders can be of great threat to children’s quality of life, and some typical presentations, such as diarrhea or constipation, easily affect well-being. Additionally, some less attractive and nontypical presentations can be of great harm to children’s growth; for example, maladaptive behaviors, sleep disorders, aggressive behavior, irritability, and self-injury may result from symptoms in ASD children who are undergoing gastrointestinal disorders (Patusco and Ziegler, 2018).

Although the cause and effect of gastrointestinal dysfunction in ASD children have not been fully elucidated, increasing evidence has shown that gut microbiota plays an important role in this process. The gut microbiota, which is composed of bacteria, fungi, and viruses, is essential in maintaining a healthy gut microenvironment for gastrointestinal function and immune and endocrine homeostasis (Osadchiy et al., 2019). Abnormal alterations in gut microbiota can lead to intestinal malfunction, thus resulting in increased intestinal permeability (also known as leaky gut), inflammation, and metabolism dysfunction (Powell et al., 2017), thus affecting distant organs or systems of the host. Increasing reports have indicated that the gut microbiota participates in a bidirectional signaling pathway between the gastrointestinal system and the brain, which is known as the microbiota-gut-brain (MGB) axis (Naveed et al., 2021). Recent studies reported that alterations in the gut microbiota composition or gut microbiota dysbiosis in children with ASD may contribute to both gastrointestinal and nervous system symptoms (Hughes et al., 2018), while several studies have suggested a potential link between the microbiota and ASD: great differences exist in the gut microbiota between children with ASD and TD controls (Fattorusso et al., 2019).

Consistent with numerous studies of diverse cohorts that have reported differences in the microbiome profiles of individuals with ASD and TD controls, we found significant differences in the gut microbiota between individuals with ASD and TD controls in this trial. We analyzed the diversity and abundance of gut microbiota in ASD populations and TD controls and observed dysbiosis in children with ASD. As our data presented, alpha and beta diversity did not show any significant differences between the TD cohort and ASD; however, gut microbiota at the phylum and genus levels were found to be variable between the two groups. We speculated that although the diversity was not statistically different, the microecology in the intestinal tract was disturbed due to alterations in several microorganisms of low abundance. In particular, in ASD populations, *Verrucomicrobia* at the phylum level was remarkably increased compared to that in TD children, which has been identified as an upregulated intestinal microorganism in many neurological disorders, such as Parkinson’s disease. It has also been reported by many other publications that the abundance of *Verrucomicrobia* is altered significantly in autism populations. In addition, at the genus level, *Ruminococcus*, *Corprococcus*, *Akkermansia*, and *Christensenellaceae* were found to be increased in ASD children, while *Romboutsia*, *Fusicatenibacter*, and *Eubacterium coprostanoligenes* were found to be significantly decreased.

To date, there is no confirmed evidence showing that autism can be cured by any medication. Currently approved and recommended treatments for ASD essentially include rehabilitation, educational therapy and psychopharmacological approaches (Famitafreshi and Karimian, 2018). Recently, microbiota intervention treatment by nutritional management and probiotic therapy has attracted increasing attention, since some trials have already reported that by reconstruction of the gut microbiota, gastrointestinal disorders, along with autism symptoms, can be alleviated (Patusco and Ziegler, 2018). Among microbiota therapies, fecal microbiota transplantation (FMT) is thought to be a preferred method due to its capacity to target the entire gut microbiota with good safety. FMT is mainly applied as an effective treatment for recurrent *Clostridium difficile* infection (Kelly et al., 2016) and has also been developed as an alternative therapy for many diseases complicated by gastrointestinal disorders. Recent studies have shown that FMT also has a therapeutic effect on neurological disorders, both in animal models and clinical trials. In 2017, Kang et al. reported that microbiota transplantation from healthy donors improves gastrointestinal and autism disorders in an open-label study (Kang et al., 2017).

To better understand the efficacy and mechanism of FMT in improving ASD symptoms, we carried out this trial, and our data showed that FMT by both the rectal route and oral administration had a significant beneficial clinical effect in children with ASD, especially in young children, which lasted for 8 weeks without inducing severe complications. ASD children who participated in this trial were allocated to oral or rectal FMT subgroups based on whether they could comply with capsule swallowing. During enrollment, we found that children with more severe symptoms (both gastrointestinal disorders and autism) tend to be not capable of cooperating and it is difficult for them to swallow FMT capsules, this leads to the variation in baseline characteristics of clinic symptoms between the oral and rectal group. Children in the rectal group suffered more from diarrhea and have more severe GI symptoms (such as abdominal pain, reflux, flatulence), also, the scale on autistic symptoms showed that they have a higher score on ABC, CARS, and lower score on SRS. Fortunately, oral FMT and rectal FMT both lead to improvement of GI symptoms and autism in participants, furthermore, after 4 consecutive weeks of FMT therapy, no significant difference in GI symptoms or autism was observed between the two subgroups, this result verified the equality of FMT through oral and rectal routes. When applying FMT for ASD children, the oral or rectal route would not cause a prominent difference in the therapeutical outcome, whether the recipient is capable of taking FMT capsules by oral is the key factor that should be taken into consideration.

Gut microbiota diversity after FMT treatment in ASD children also showed significant differences, which implied that improvement of symptoms is associated with the change in gut microecology. It is believed that the bidirectional interaction between the brain and gut through the MGB axis is associated with neuroendocrine, neuroimmune, and autonomic nervous system mechanisms. An increasing number of publications have reported that gut microbiota can affect the metabolism of neurotransmitters. For example, some metabolites derived from gut microbiota can be absorbed and enter the blood circulation, and these metabolites can cross the blood-brain barrier and modulate cerebral function (Fung et al., 2017). *Lactobacillus rhamnosus* YS9 can produce gamma-aminobutyric acid (GABA), an important inhibitory neurotransmitter in the system, while monoamines, such as noradrenaline, dopamine, and serotonin, are also produced by several strains of bacteria colonizing the intestinal tract (Strandwitz, 2018). Take GABA as an example, which is derived from glutamate thanks to the action of glutamate decarboxylase, it is important in neuronal excitability, alterations in gabaminergic and glutaminergic systems would cause a disrupted excitatory/inhibitory balance, and it is found that plasma level of GABA in ASD children is altered compared to healthy cohorts (Marotta et al., 2020). As for 5-HT, it intervenes in multiple brain functions, studies have revealed that the 5-HT transporter (SERT or 5-HTT) or 5-HT levels were higher in autistic individuals both in clinic studies and in animal models (Muller et al., 2016). Based on current evidence, we evaluated the serum level of neurotransmitters in participants to better understand the possible mechanism by which FMT showed a therapeutic effect on ASD. We found that FMT in the ASD cohort induced a significant change in neurotransmitters in serum; 5-HT and GABA decreased after FMT, while DA levels were elevated. As our results show, we speculate that FMT might be helpful in modulating the central nerve through the MGB axis by regulating neurotransmitters.

In this trial, we also found that the gut microbiota α diversity did not change significantly, suggesting that FMT did not affect the overall structure of gut microbial communities, or at least, was not sufficient to induce a response in each participant. In addition, FMT treatment could promote the colonization of donor microbes and shift the bacterial community of individuals with ASD toward that of TD controls or donors. Because the pre-existing intestinal landscape can dramatically affect the outcome of FMT, we assessed the composition of the gut microbiota pre-FMT and divided the participants from the ASD group into two subgroups, responders and nonresponders to FMT, according to the microbiota analysis. We found that responders and nonresponders clustered separately and that a high abundance of *Eubacterium coprostanoligenes* in ASD patients prior to FMT was associated with high GSRS scores. We thus hypothesized that in FMT clinical responders, FMT might reduce the abundance of *Eubacterium coprostanoligenes*, thus improving GI symptoms in individuals with ASD. We further found that the reduction in *Eubacterium coprostanoligenes* abundance was positively correlated with a reduction in GI symptoms, indicating an association between clinical improvements and the changes in bacterial profiles following FMT in ASD patients. Moreover, a negative correlation exists between *Eubacterium coprostanoligenes* abundance and serum GABA concentration, indicating that FMT might have an influence on neurotransmitters that would regulate mood, behavior, and neurodevelopment, and this might be a possible explanation that why FMT induced improvement not only GI symptoms but also autistic symptoms on ASD children.

In conclusion, our work proved that FMT was well tolerated and effective in improving gastrointestinal symptoms and autism-like behaviors in children with ASD. FMT seemed to induce the production of a microbiota that was significantly different from the pre-FMT microbiota and much more similar to those of the healthy donor and typically developing children. However, this study did not include children under other therapies as the control group, and only include participants and donor in southwest China, whether geological factors would have impact on the therapeutical outcomes in ASD children is still lack of evidence. Our findings identified specific bacteria *Eubacterium coprostanoligenes* that might be associated with therapeutic outcomes, which should be further explored in future FMT trials in ASD patients.

## Data Availability Statement

The data presented in the study are deposited in the NCBI Sequence Read Archive, accession number PRJNA758217.

## Ethics Statement

The present clinical trial study was reviewed and approved by the Ethics Committee at the Army Medical Center of PLA. Written informed consent to participate in this study was provided by the participants’ legal guardian/next of kin.

## Author Contributions

NL, DC, and YW conceived the idea. NL, HC, YC, FX, SY, LC, MC, LL, YP, and WT performed the experiments and analyzed the data. YC, NL, and WT wrote the manuscript. DC and YW supervised the study. NL, HC, and YC contributed equally to this work. All authors contributed to the article and approved the submitted version.

## Funding

The study was supported by Army Medical University (Grant No: 2017XYY06), Military Science and Technology Innovation Project (Grant No: 17-163-12-ZT-002-060-01), Key Science and Health Joint Project of Chongqing (Grant No: 2019ZDXM026 and 2020MSXM017).

## Conflict of Interest

The authors declare that the research was conducted in the absence of any commercial or financial relationships that could be construed as a potential conflict of interest.

## Publisher’s Note

All claims expressed in this article are solely those of the authors and do not necessarily represent those of their affiliated organizations, or those of the publisher, the editors and the reviewers. Any product that may be evaluated in this article, or claim that may be made by its manufacturer, is not guaranteed or endorsed by the publisher.
