# Corrigendum: Fecal Microbiota Transplantation Relieves Gastrointestinal and Autism Symptoms by Improving the Gut Microbiota in an Open-Label Study

**DOI:** 10.3389/fcimb.2021.801376

**Published:** 2021-11-23

**Authors:** Ning Li, Hongyan Chen, Yi Cheng, Fenghua Xu, Guangcong Ruan, Senhong Ying, Wen Tang, Lu Chen, Minjia Chen, LinLing Lv, Yi Ping, Dongfeng Chen, Yanling Wei

**Affiliations:** ^1^ Department of gastroenterology, Daping Hospital, Army Medical University (Third Military Medical University), Chongqing, China; ^2^ Department of Gastroenterology, North-Kuanren General Hospital, Chongqing, China

**Keywords:** gut microbiota, fecal microbiota transplantation, autism spectrum disorders, microbiome-gut-brain axis, clinic trial

In the original article, there was a mistake in ***Figure 1*** as published. **The dose of oral route FMT was mislabeled.** The corrected ***Figure 1*** appears below.

**Figure 1:**
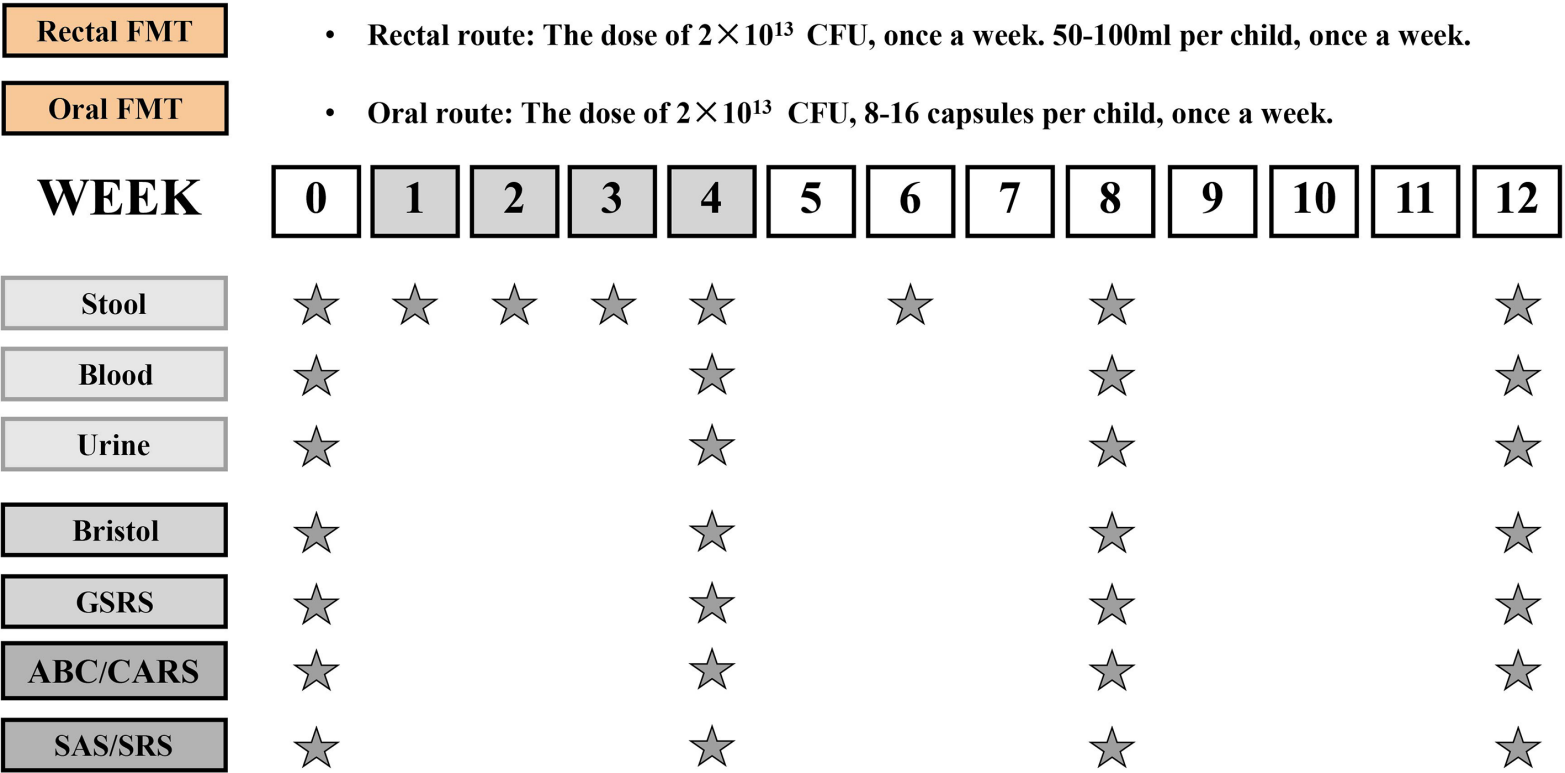
Study design timeline. The trial consisted of a 4-week period of FMT and an 8-week follow-up observation period after the end of treatment. The time schedule of sample collection and GI/behavioral assessments.

The authors apologize for this error and state that this does not change the scientific conclusions of the article in any way. The original article has been updated.

## Publisher’s Note

All claims expressed in this article are solely those of the authors and do not necessarily represent those of their affiliated organizations, or those of the publisher, the editors and the reviewers. Any product that may be evaluated in this article, or claim that may be made by its manufacturer, is not guaranteed or endorsed by the publisher.

