# Supplementary material for: Fecal Microbiota Transplantation Relieves Gastrointestinal and Autism Symptoms by Improving the Gut Microbiota in an Open-Label Study
Source: Front Cell Infect Microbiol. 2021 Oct 19;11:759435. doi: 10.3389/fcimb.2021.759435 (PMC8560686; doi:10.3389/fcimb.2021.759435)
Supplement: Supplementary file 1 [file DataSheet_1.zip › raw data/Figure 2/CARS+CBCL+ABC+SAS/ABC-Oral statistics.tif.doc]

ONEWAY VAR00001 BY VAR00002
  /STATISTICS DESCRIPTIVES HOMOGENEITY
  /MISSING ANALYSIS
  /POSTHOC=LSD T2 ALPHA(0.05).


Oneway


附注	
已创建输出	14-SEP-2019 14:46:39	
注释		
输入	过滤器	<无>	
	宽度(W)	<无>	
	拆分文件	<无>	
	工作数据文件中的行数	108	
缺失值处理	缺失定义	用户定义的缺失值视为缺失。	
	使用的个案	每个分析的统计量都基于对于该分析中的任意变量都没有缺失数据的个案。	
语法	ONEWAY VAR00001 BY VAR00002
  /STATISTICS DESCRIPTIVES HOMOGENEITY
  /MISSING ANALYSIS
  /POSTHOC=LSD T2 ALPHA(0.05).	
资源	处理器时间	00:00:00.02	
	用时	00:00:00.02	


描述性	
VAR00001  	
	N	平均值	标准 偏差	标准 错误	平均值 95% 置信区间	最小值	最大值	
					下限值	上限			
1.00	27	41.8519	15.96319	3.07212	35.5370	48.1667	11.00	72.00	
2.00	27	25.2963	15.46165	2.97560	19.1799	31.4127	1.00	58.00	
3.00	27	29.8148	15.08702	2.90350	23.8466	35.7830	4.00	58.00	
4.00	27	33.3704	15.66758	3.01523	27.1725	39.5683	5.00	60.00	
总计	108	32.5833	16.49646	1.58737	29.4366	35.7301	1.00	72.00	


方差同质性检验	
VAR00001  	
Levene 统计	df1	df2	显著性	
.081	3	104	.970	


ANOVA	
VAR00001  	
	平方和	df	均方	F	显著性	
组之间	3976.843	3	1325.614	5.484	.002	
组内	25141.407	104	241.744			
总计	29118.250	107				


事后检验


多重比较	
因变量:   VAR00001  	
	(I) VAR00002	(J) VAR00002	平均差 (I-J)	标准 错误	显著性	95% 置信区间	
						下限值	
LSD(L)	1.00	2.00	16.55556*	4.23166	.000	8.1640	
		3.00	12.03704*	4.23166	.005	3.6455	
		4.00	8.48148*	4.23166	.048	.0899	
	2.00	1.00	-16.55556*	4.23166	.000	-24.9471	
		3.00	-4.51852	4.23166	.288	-12.9101	
		4.00	-8.07407	4.23166	.059	-16.4656	
	3.00	1.00	-12.03704*	4.23166	.005	-20.4286	
		2.00	4.51852	4.23166	.288	-3.8730	
		4.00	-3.55556	4.23166	.403	-11.9471	
	4.00	1.00	-8.48148*	4.23166	.048	-16.8730	
		2.00	8.07407	4.23166	.059	-.3175	
		3.00	3.55556	4.23166	.403	-4.8360	
Tamhane	1.00	2.00	16.55556*	4.27692	.002	4.8580	
		3.00	12.03704*	4.22708	.037	.4748	
		4.00	8.48148	4.30459	.284	-3.2914	
	2.00	1.00	-16.55556*	4.27692	.002	-28.2531	
		3.00	-4.51852	4.15746	.863	-15.8892	
		4.00	-8.07407	4.23624	.320	-19.6600	
	3.00	1.00	-12.03704*	4.22708	.037	-23.5993	
		2.00	4.51852	4.15746	.863	-6.8521	
		4.00	-3.55556	4.18592	.953	-15.0044	
	4.00	1.00	-8.48148	4.30459	.284	-20.2544	
		2.00	8.07407	4.23624	.320	-3.5118	
		3.00	3.55556	4.18592	.953	-7.8933	

多重比较	
因变量:   VAR00001  	
	(I) VAR00002	(J) VAR00002	95% 置信区间	
			上限	
LSD(L)	1.00	2.00	24.9471	
		3.00	20.4286	
		4.00	16.8730	
	2.00	1.00	-8.1640	
		3.00	3.8730	
		4.00	.3175	
	3.00	1.00	-3.6455	
		2.00	12.9101	
		4.00	4.8360	
	4.00	1.00	-.0899	
		2.00	16.4656	
		3.00	11.9471	
Tamhane	1.00	2.00	28.2531	
		3.00	23.5993	
		4.00	20.2544	
	2.00	1.00	-4.8580	
		3.00	6.8521	
		4.00	3.5118	
	3.00	1.00	-.4748	
		2.00	15.8892	
		4.00	7.8933	
	4.00	1.00	3.2914	
		2.00	19.6600	
		3.00	15.0044	

*. 均值差的显著性水平为 0.05。	
