# Supplementary material for: Fecal Microbiota Transplantation Relieves Gastrointestinal and Autism Symptoms by Improving the Gut Microbiota in an Open-Label Study
Source: Front Cell Infect Microbiol. 2021 Oct 19;11:759435. doi: 10.3389/fcimb.2021.759435 (PMC8560686; doi:10.3389/fcimb.2021.759435)
Supplement: Supplementary file 1 [file DataSheet_1.zip › raw data/Figure 2/CARS+CBCL+ABC+SAS/ABC-Rectal statistics.tif.doc]

ONEWAY VAR00001 BY VAR00002
  /STATISTICS DESCRIPTIVES HOMOGENEITY
  /MISSING ANALYSIS
  /POSTHOC=LSD T2 ALPHA(0.05).


Oneway


附注	
已创建输出	14-SEP-2019 14:56:05	
注释		
输入	过滤器	<无>	
	宽度(W)	<无>	
	拆分文件	<无>	
	工作数据文件中的行数	108	
缺失值处理	缺失定义	用户定义的缺失值视为缺失。	
	使用的个案	每个分析的统计量都基于对于该分析中的任意变量都没有缺失数据的个案。	
语法	ONEWAY VAR00001 BY VAR00002
  /STATISTICS DESCRIPTIVES HOMOGENEITY
  /MISSING ANALYSIS
  /POSTHOC=LSD T2 ALPHA(0.05).	
资源	处理器时间	00:00:00.02	
	用时	00:00:00.02	


描述性	
VAR00001  	
	N	平均值	标准 偏差	标准 错误	平均值 95% 置信区间	最小值	最大值	
					下限值	上限			
1.00	13	41.6154	19.44882	5.39413	29.8626	53.3682	17.00	73.00	
2.00	13	25.5385	12.03201	3.33708	18.2676	32.8093	10.00	45.00	
3.00	13	33.1538	13.26553	3.67920	25.1376	41.1701	11.00	50.00	
4.00	13	34.0000	12.96148	3.59487	26.1675	41.8325	13.00	54.00	
总计	52	33.5769	15.39588	2.13502	29.2907	37.8632	10.00	73.00	


方差同质性检验	
VAR00001  	
Levene 统计	df1	df2	显著性	
1.921	3	48	.139	


ANOVA	
VAR00001  	
	平方和	df	均方	F	显著性	
组之间	1684.692	3	561.564	2.591	.064	
组内	10404.000	48	216.750			
总计	12088.692	51				


事后检验


多重比较	
因变量:   VAR00001  	
	(I) VAR00002	(J) VAR00002	平均差 (I-J)	标准 错误	显著性	95% 置信区间	
						下限值	
LSD(L)	1.00	2.00	16.07692*	5.77461	.008	4.4663	
		3.00	8.46154	5.77461	.149	-3.1491	
		4.00	7.61538	5.77461	.194	-3.9953	
	2.00	1.00	-16.07692*	5.77461	.008	-27.6876	
		3.00	-7.61538	5.77461	.194	-19.2260	
		4.00	-8.46154	5.77461	.149	-20.0722	
	3.00	1.00	-8.46154	5.77461	.149	-20.0722	
		2.00	7.61538	5.77461	.194	-3.9953	
		4.00	-.84615	5.77461	.884	-12.4568	
	4.00	1.00	-7.61538	5.77461	.194	-19.2260	
		2.00	8.46154	5.77461	.149	-3.1491	
		3.00	.84615	5.77461	.884	-10.7645	
Tamhane	1.00	2.00	16.07692	6.34293	.113	-2.4281	
		3.00	8.46154	6.52941	.755	-10.4750	
		4.00	7.61538	6.48226	.827	-11.2096	
	2.00	1.00	-16.07692	6.34293	.113	-34.5819	
		3.00	-7.61538	4.96715	.591	-21.8633	
		4.00	-8.46154	4.90501	.459	-22.5263	
	3.00	1.00	-8.46154	6.52941	.755	-27.3981	
		2.00	7.61538	4.96715	.591	-6.6325	
		4.00	-.84615	5.14389	1.000	-15.5893	
	4.00	1.00	-7.61538	6.48226	.827	-26.4404	
		2.00	8.46154	4.90501	.459	-5.6032	
		3.00	.84615	5.14389	1.000	-13.8970	

多重比较	
因变量:   VAR00001  	
	(I) VAR00002	(J) VAR00002	95% 置信区间	
			上限	
LSD(L)	1.00	2.00	27.6876	
		3.00	20.0722	
		4.00	19.2260	
	2.00	1.00	-4.4663	
		3.00	3.9953	
		4.00	3.1491	
	3.00	1.00	3.1491	
		2.00	19.2260	
		4.00	10.7645	
	4.00	1.00	3.9953	
		2.00	20.0722	
		3.00	12.4568	
Tamhane	1.00	2.00	34.5819	
		3.00	27.3981	
		4.00	26.4404	
	2.00	1.00	2.4281	
		3.00	6.6325	
		4.00	5.6032	
	3.00	1.00	10.4750	
		2.00	21.8633	
		4.00	13.8970	
	4.00	1.00	11.2096	
		2.00	22.5263	
		3.00	15.5893	

*. 均值差的显著性水平为 0.05。	
