# Supplementary material for: Fecal Microbiota Transplantation Relieves Gastrointestinal and Autism Symptoms by Improving the Gut Microbiota in an Open-Label Study
Source: Front Cell Infect Microbiol. 2021 Oct 19;11:759435. doi: 10.3389/fcimb.2021.759435 (PMC8560686; doi:10.3389/fcimb.2021.759435)
Supplement: Supplementary file 1 [file DataSheet_1.zip › raw data/Figure 2/CARS+CBCL+ABC+SAS/CARS-Rectal statistics.tif.doc]

ONEWAY VAR00001 BY VAR00002
  /STATISTICS DESCRIPTIVES HOMOGENEITY
  /MISSING ANALYSIS
  /POSTHOC=LSD T2 ALPHA(0.05).


Oneway


附注	
已创建输出	14-SEP-2019 14:50:17	
注释		
输入	过滤器	<无>	
	宽度(W)	<无>	
	拆分文件	<无>	
	工作数据文件中的行数	108	
缺失值处理	缺失定义	用户定义的缺失值视为缺失。	
	使用的个案	每个分析的统计量都基于对于该分析中的任意变量都没有缺失数据的个案。	
语法	ONEWAY VAR00001 BY VAR00002
  /STATISTICS DESCRIPTIVES HOMOGENEITY
  /MISSING ANALYSIS
  /POSTHOC=LSD T2 ALPHA(0.05).	
资源	处理器时间	00:00:00.00	
	用时	00:00:00.00	


描述性	
VAR00001  	
	N	平均值	标准 偏差	标准 错误	平均值 95% 置信区间	最小值	最大值	
					下限值	上限			
1.00	13	37.0769	8.06703	2.23739	32.2021	41.9518	25.00	52.00	
2.00	13	26.9231	5.26722	1.46086	23.7401	30.1060	18.00	36.00	
3.00	13	29.6923	4.42313	1.22676	27.0194	32.3652	24.00	38.00	
4.00	13	31.9231	4.94067	1.37030	28.9375	34.9087	25.00	42.00	
总计	52	31.4038	6.80617	.94385	29.5090	33.2987	18.00	52.00	


方差同质性检验	
VAR00001  	
Levene 统计	df1	df2	显著性	
1.666	3	48	.187	


ANOVA	
VAR00001  	
	平方和	df	均方	F	显著性	
组之间	720.981	3	240.327	7.027	.001	
组内	1641.538	48	34.199			
总计	2362.519	51				


事后检验


多重比较	
因变量:   VAR00001  	
	(I) VAR00002	(J) VAR00002	平均差 (I-J)	标准 错误	显著性	95% 置信区间	
						下限值	
LSD(L)	1.00	2.00	10.15385*	2.29376	.000	5.5419	
		3.00	7.38462*	2.29376	.002	2.7727	
		4.00	5.15385*	2.29376	.029	.5419	
	2.00	1.00	-10.15385*	2.29376	.000	-14.7658	
		3.00	-2.76923	2.29376	.233	-7.3811	
		4.00	-5.00000*	2.29376	.034	-9.6119	
	3.00	1.00	-7.38462*	2.29376	.002	-11.9965	
		2.00	2.76923	2.29376	.233	-1.8427	
		4.00	-2.23077	2.29376	.336	-6.8427	
	4.00	1.00	-5.15385*	2.29376	.029	-9.7658	
		2.00	5.00000*	2.29376	.034	.3881	
		3.00	2.23077	2.29376	.336	-2.3811	
Tamhane	1.00	2.00	10.15385*	2.67209	.006	2.3844	
		3.00	7.38462	2.55164	.055	-.1201	
		4.00	5.15385	2.62367	.326	-2.5055	
	2.00	1.00	-10.15385*	2.67209	.006	-17.9233	
		3.00	-2.76923	1.90763	.649	-8.2510	
		4.00	-5.00000	2.00296	.113	-10.7426	
	3.00	1.00	-7.38462	2.55164	.055	-14.8893	
		2.00	2.76923	1.90763	.649	-2.7126	
		4.00	-2.23077	1.83920	.803	-7.5076	
	4.00	1.00	-5.15385	2.62367	.326	-12.8132	
		2.00	5.00000	2.00296	.113	-.7426	
		3.00	2.23077	1.83920	.803	-3.0461	

多重比较	
因变量:   VAR00001  	
	(I) VAR00002	(J) VAR00002	95% 置信区间	
			上限	
LSD(L)	1.00	2.00	14.7658	
		3.00	11.9965	
		4.00	9.7658	
	2.00	1.00	-5.5419	
		3.00	1.8427	
		4.00	-.3881	
	3.00	1.00	-2.7727	
		2.00	7.3811	
		4.00	2.3811	
	4.00	1.00	-.5419	
		2.00	9.6119	
		3.00	6.8427	
Tamhane	1.00	2.00	17.9233	
		3.00	14.8893	
		4.00	12.8132	
	2.00	1.00	-2.3844	
		3.00	2.7126	
		4.00	.7426	
	3.00	1.00	.1201	
		2.00	8.2510	
		4.00	3.0461	
	4.00	1.00	2.5055	
		2.00	10.7426	
		3.00	7.5076	

*. 均值差的显著性水平为 0.05。	
