# Supplementary material for: Fecal Microbiota Transplantation Relieves Gastrointestinal and Autism Symptoms by Improving the Gut Microbiota in an Open-Label Study
Source: Front Cell Infect Microbiol. 2021 Oct 19;11:759435. doi: 10.3389/fcimb.2021.759435 (PMC8560686; doi:10.3389/fcimb.2021.759435)
Supplement: Supplementary file 1 [file DataSheet_1.zip › raw data/Figure 2/CARS+CBCL+ABC+SAS/CBCL-Oral statistics.tif.doc]

ONEWAY VAR00001 BY VAR00002
  /STATISTICS DESCRIPTIVES HOMOGENEITY
  /MISSING ANALYSIS
  /POSTHOC=LSD T2 ALPHA(0.05).


Oneway


附注	
已创建输出	14-SEP-2019 14:45:14	
注释		
输入	过滤器	<无>	
	宽度(W)	<无>	
	拆分文件	<无>	
	工作数据文件中的行数	108	
缺失值处理	缺失定义	用户定义的缺失值视为缺失。	
	使用的个案	每个分析的统计量都基于对于该分析中的任意变量都没有缺失数据的个案。	
语法	ONEWAY VAR00001 BY VAR00002
  /STATISTICS DESCRIPTIVES HOMOGENEITY
  /MISSING ANALYSIS
  /POSTHOC=LSD T2 ALPHA(0.05).	
资源	处理器时间	00:00:00.02	
	用时	00:00:00.02	


描述性	
VAR00001  	
	N	平均值	标准 偏差	标准 错误	平均值 95% 置信区间	最小值	最大值	
					下限值	上限			
1.00	27	57.1481	23.27214	4.47873	47.9420	66.3543	24.00	110.00	
2.00	27	39.8148	21.81396	4.19810	31.1855	48.4441	9.00	94.00	
3.00	27	43.6296	21.44608	4.12730	35.1458	52.1134	18.00	98.00	
4.00	27	47.2222	21.82506	4.20024	38.5885	55.8559	22.00	102.00	
总计	108	46.9537	22.72936	2.18713	42.6180	51.2894	9.00	110.00	


方差同质性检验	
VAR00001  	
Levene 统计	df1	df2	显著性	
.151	3	104	.929	


ANOVA	
VAR00001  	
	平方和	df	均方	F	显著性	
组之间	4482.324	3	1494.108	3.059	.032	
组内	50796.444	104	488.427			
总计	55278.769	107				


事后检验


多重比较	
因变量:   VAR00001  	
	(I) VAR00002	(J) VAR00002	平均差 (I-J)	标准 错误	显著性	95% 置信区间	
						下限值	
LSD(L)	1.00	2.00	17.33333*	6.01496	.005	5.4054	
		3.00	13.51852*	6.01496	.027	1.5906	
		4.00	9.92593	6.01496	.102	-2.0020	
	2.00	1.00	-17.33333*	6.01496	.005	-29.2612	
		3.00	-3.81481	6.01496	.527	-15.7427	
		4.00	-7.40741	6.01496	.221	-19.3353	
	3.00	1.00	-13.51852*	6.01496	.027	-25.4464	
		2.00	3.81481	6.01496	.527	-8.1131	
		4.00	-3.59259	6.01496	.552	-15.5205	
	4.00	1.00	-9.92593	6.01496	.102	-21.8538	
		2.00	7.40741	6.01496	.221	-4.5205	
		3.00	3.59259	6.01496	.552	-8.3353	
Tamhane	1.00	2.00	17.33333*	6.13865	.040	.5418	
		3.00	13.51852	6.09045	.171	-3.1428	
		4.00	9.92593	6.14011	.510	-6.8696	
	2.00	1.00	-17.33333*	6.13865	.040	-34.1249	
		3.00	-3.81481	5.88716	.988	-19.9160	
		4.00	-7.40741	5.93852	.771	-23.6488	
	3.00	1.00	-13.51852	6.09045	.171	-30.1798	
		2.00	3.81481	5.88716	.988	-12.2863	
		4.00	-3.59259	5.88868	.991	-19.6979	
	4.00	1.00	-9.92593	6.14011	.510	-26.7214	
		2.00	7.40741	5.93852	.771	-8.8340	
		3.00	3.59259	5.88868	.991	-12.5127	

多重比较	
因变量:   VAR00001  	
	(I) VAR00002	(J) VAR00002	95% 置信区间	
			上限	
LSD(L)	1.00	2.00	29.2612	
		3.00	25.4464	
		4.00	21.8538	
	2.00	1.00	-5.4054	
		3.00	8.1131	
		4.00	4.5205	
	3.00	1.00	-1.5906	
		2.00	15.7427	
		4.00	8.3353	
	4.00	1.00	2.0020	
		2.00	19.3353	
		3.00	15.5205	
Tamhane	1.00	2.00	34.1249	
		3.00	30.1798	
		4.00	26.7214	
	2.00	1.00	-.5418	
		3.00	12.2863	
		4.00	8.8340	
	3.00	1.00	3.1428	
		2.00	19.9160	
		4.00	12.5127	
	4.00	1.00	6.8696	
		2.00	23.6488	
		3.00	19.6979	

*. 均值差的显著性水平为 0.05。	
