# Supplementary material for: Fecal Microbiota Transplantation Relieves Gastrointestinal and Autism Symptoms by Improving the Gut Microbiota in an Open-Label Study
Source: Front Cell Infect Microbiol. 2021 Oct 19;11:759435. doi: 10.3389/fcimb.2021.759435 (PMC8560686; doi:10.3389/fcimb.2021.759435)
Supplement: Supplementary file 1 [file DataSheet_1.zip › raw data/Figure 2/CARS+CBCL+ABC+SAS/CBCL-Rectal statistics.tif.doc]

ONEWAY VAR00001 BY VAR00002
  /STATISTICS DESCRIPTIVES HOMOGENEITY
  /MISSING ANALYSIS
  /POSTHOC=LSD T2 ALPHA(0.05).


Oneway


附注	
已创建输出	14-SEP-2019 14:54:46	
注释		
输入	过滤器	<无>	
	宽度(W)	<无>	
	拆分文件	<无>	
	工作数据文件中的行数	108	
缺失值处理	缺失定义	用户定义的缺失值视为缺失。	
	使用的个案	每个分析的统计量都基于对于该分析中的任意变量都没有缺失数据的个案。	
语法	ONEWAY VAR00001 BY VAR00002
  /STATISTICS DESCRIPTIVES HOMOGENEITY
  /MISSING ANALYSIS
  /POSTHOC=LSD T2 ALPHA(0.05).	
资源	处理器时间	00:00:00.02	
	用时	00:00:00.02	


描述性	
VAR00001  	
	N	平均值	标准 偏差	标准 错误	平均值 95% 置信区间	最小值	最大值	
					下限值	上限			
1.00	13	69.4615	19.74679	5.47678	57.5287	81.3944	38.00	101.00	
2.00	13	45.3846	11.35443	3.14915	38.5232	52.2460	27.00	63.00	
3.00	13	43.4615	9.17075	2.54351	37.9197	49.0034	31.00	60.00	
4.00	13	54.0000	14.31782	3.97105	45.3478	62.6522	32.00	78.00	
总计	52	53.0769	17.24546	2.39151	48.2758	57.8781	27.00	101.00	


方差同质性检验	
VAR00001  	
Levene 统计	df1	df2	显著性	
2.900	3	48	.044	


ANOVA	
VAR00001  	
	平方和	df	均方	F	显著性	
组之间	5472.154	3	1824.051	9.030	.000	
组内	9695.538	48	201.990			
总计	15167.692	51				


事后检验


多重比较	
因变量:   VAR00001  	
	(I) VAR00002	(J) VAR00002	平均差 (I-J)	标准 错误	显著性	95% 置信区间	
						下限值	
LSD(L)	1.00	2.00	24.07692*	5.57454	.000	12.8686	
		3.00	26.00000*	5.57454	.000	14.7916	
		4.00	15.46154*	5.57454	.008	4.2532	
	2.00	1.00	-24.07692*	5.57454	.000	-35.2853	
		3.00	1.92308	5.57454	.732	-9.2853	
		4.00	-8.61538	5.57454	.129	-19.8237	
	3.00	1.00	-26.00000*	5.57454	.000	-37.2084	
		2.00	-1.92308	5.57454	.732	-13.1314	
		4.00	-10.53846	5.57454	.065	-21.7468	
	4.00	1.00	-15.46154*	5.57454	.008	-26.6699	
		2.00	8.61538	5.57454	.129	-2.5930	
		3.00	10.53846	5.57454	.065	-.6699	
Tamhane	1.00	2.00	24.07692*	6.31761	.007	5.5564	
		3.00	26.00000*	6.03858	.003	8.0336	
		4.00	15.46154	6.76493	.179	-4.0941	
	2.00	1.00	-24.07692*	6.31761	.007	-42.5975	
		3.00	1.92308	4.04804	.998	-9.7243	
		4.00	-8.61538	5.06817	.478	-23.2079	
	3.00	1.00	-26.00000*	6.03858	.003	-43.9664	
		2.00	-1.92308	4.04804	.998	-13.5705	
		4.00	-10.53846	4.71579	.201	-24.2663	
	4.00	1.00	-15.46154	6.76493	.179	-35.0172	
		2.00	8.61538	5.06817	.478	-5.9772	
		3.00	10.53846	4.71579	.201	-3.1894	

多重比较	
因变量:   VAR00001  	
	(I) VAR00002	(J) VAR00002	95% 置信区间	
			上限	
LSD(L)	1.00	2.00	35.2853	
		3.00	37.2084	
		4.00	26.6699	
	2.00	1.00	-12.8686	
		3.00	13.1314	
		4.00	2.5930	
	3.00	1.00	-14.7916	
		2.00	9.2853	
		4.00	.6699	
	4.00	1.00	-4.2532	
		2.00	19.8237	
		3.00	21.7468	
Tamhane	1.00	2.00	42.5975	
		3.00	43.9664	
		4.00	35.0172	
	2.00	1.00	-5.5564	
		3.00	13.5705	
		4.00	5.9772	
	3.00	1.00	-8.0336	
		2.00	9.7243	
		4.00	3.1894	
	4.00	1.00	4.0941	
		2.00	23.2079	
		3.00	24.2663	

*. 均值差的显著性水平为 0.05。	
