# Supplementary material for: Fecal Microbiota Transplantation Relieves Gastrointestinal and Autism Symptoms by Improving the Gut Microbiota in an Open-Label Study
Source: Front Cell Infect Microbiol. 2021 Oct 19;11:759435. doi: 10.3389/fcimb.2021.759435 (PMC8560686; doi:10.3389/fcimb.2021.759435)
Supplement: Supplementary file 1 [file DataSheet_1.zip › raw data/Figure 2/CARS+CBCL+ABC+SAS/SAS-Oral statistics.tif.doc]

ONEWAY VAR00001 BY VAR00002
  /STATISTICS DESCRIPTIVES HOMOGENEITY
  /MISSING ANALYSIS
  /POSTHOC=LSD T2 ALPHA(0.05).


Oneway


附注	
已创建输出	14-SEP-2019 14:47:48	
注释		
输入	过滤器	<无>	
	宽度(W)	<无>	
	拆分文件	<无>	
	工作数据文件中的行数	108	
缺失值处理	缺失定义	用户定义的缺失值视为缺失。	
	使用的个案	每个分析的统计量都基于对于该分析中的任意变量都没有缺失数据的个案。	
语法	ONEWAY VAR00001 BY VAR00002
  /STATISTICS DESCRIPTIVES HOMOGENEITY
  /MISSING ANALYSIS
  /POSTHOC=LSD T2 ALPHA(0.05).	
资源	处理器时间	00:00:00.00	
	用时	00:00:00.00	


描述性	
VAR00001  	
	N	平均值	标准 偏差	标准 错误	平均值 95% 置信区间	最小值	最大值	
					下限值	上限			
1.00	27	62.9630	16.01077	3.08127	56.6293	69.2966	31.00	98.00	
2.00	27	46.7407	13.06961	2.51525	41.5706	51.9109	29.00	85.00	
3.00	27	50.2593	15.49340	2.98171	44.1303	56.3882	30.00	86.00	
4.00	27	53.1852	14.42497	2.77609	47.4789	58.8915	34.00	87.00	
总计	108	53.2870	15.79368	1.51975	50.2743	56.2998	29.00	98.00	


方差同质性检验	
VAR00001  	
Levene 统计	df1	df2	显著性	
.378	3	104	.769	


ANOVA	
VAR00001  	
	平方和	df	均方	F	显著性	
组之间	3932.694	3	1310.898	5.991	.001	
组内	22757.407	104	218.821			
总计	26690.102	107				


事后检验


多重比较	
因变量:   VAR00001  	
	(I) VAR00002	(J) VAR00002	平均差 (I-J)	标准 错误	显著性	95% 置信区间	
						下限值	
LSD(L)	1.00	2.00	16.22222*	4.02604	.000	8.2384	
		3.00	12.70370*	4.02604	.002	4.7199	
		4.00	9.77778*	4.02604	.017	1.7940	
	2.00	1.00	-16.22222*	4.02604	.000	-24.2060	
		3.00	-3.51852	4.02604	.384	-11.5023	
		4.00	-6.44444	4.02604	.112	-14.4282	
	3.00	1.00	-12.70370*	4.02604	.002	-20.6875	
		2.00	3.51852	4.02604	.384	-4.4653	
		4.00	-2.92593	4.02604	.469	-10.9097	
	4.00	1.00	-9.77778*	4.02604	.017	-17.7616	
		2.00	6.44444	4.02604	.112	-1.5393	
		3.00	2.92593	4.02604	.469	-5.0579	
Tamhane	1.00	2.00	16.22222*	3.97753	.001	5.3268	
		3.00	12.70370*	4.28775	.027	.9765	
		4.00	9.77778	4.14740	.126	-1.5699	
	2.00	1.00	-16.22222*	3.97753	.001	-27.1177	
		3.00	-3.51852	3.90090	.938	-14.1992	
		4.00	-6.44444	3.74608	.437	-16.6936	
	3.00	1.00	-12.70370*	4.28775	.027	-24.4309	
		2.00	3.51852	3.90090	.938	-7.1621	
		4.00	-2.92593	4.07397	.979	-14.0702	
	4.00	1.00	-9.77778	4.14740	.126	-21.1255	
		2.00	6.44444	3.74608	.437	-3.8047	
		3.00	2.92593	4.07397	.979	-8.2183	

多重比较	
因变量:   VAR00001  	
	(I) VAR00002	(J) VAR00002	95% 置信区间	
			上限	
LSD(L)	1.00	2.00	24.2060	
		3.00	20.6875	
		4.00	17.7616	
	2.00	1.00	-8.2384	
		3.00	4.4653	
		4.00	1.5393	
	3.00	1.00	-4.7199	
		2.00	11.5023	
		4.00	5.0579	
	4.00	1.00	-1.7940	
		2.00	14.4282	
		3.00	10.9097	
Tamhane	1.00	2.00	27.1177	
		3.00	24.4309	
		4.00	21.1255	
	2.00	1.00	-5.3268	
		3.00	7.1621	
		4.00	3.8047	
	3.00	1.00	-.9765	
		2.00	14.1992	
		4.00	8.2183	
	4.00	1.00	1.5699	
		2.00	16.6936	
		3.00	14.0702	

*. 均值差的显著性水平为 0.05。	
