# Supplementary material for: Fecal Microbiota Transplantation Relieves Gastrointestinal and Autism Symptoms by Improving the Gut Microbiota in an Open-Label Study
Source: Front Cell Infect Microbiol. 2021 Oct 19;11:759435. doi: 10.3389/fcimb.2021.759435 (PMC8560686; doi:10.3389/fcimb.2021.759435)
Supplement: Supplementary file 1 [file DataSheet_1.zip › raw data/Figure 2/GSRS/GSRS subgroup-Indigestion statistics.doc]

ONEWAY VAR00007 BY VAR00008
  /STATISTICS DESCRIPTIVES HOMOGENEITY
  /MISSING ANALYSIS
  /POSTHOC=LSD T2 ALPHA(0.05).


Oneway


附注	
已创建输出	12-SEP-2019 22:34:18	
注释		
输入	活动数据集	数据集1	
	过滤器	<无>	
	宽度(W)	<无>	
	拆分文件	<无>	
	工作数据文件中的行数	160	
缺失值处理	缺失定义	用户定义的缺失值视为缺失。	
	使用的个案	每个分析的统计量都基于对于该分析中的任意变量都没有缺失数据的个案。	
语法	ONEWAY VAR00007 BY VAR00008
  /STATISTICS DESCRIPTIVES HOMOGENEITY
  /MISSING ANALYSIS
  /POSTHOC=LSD T2 ALPHA(0.05).	
资源	处理器时间	00:00:00.02	
	用时	00:00:00.01	


描述性	
VAR00007  	
	N	平均值	标准 偏差	标准 错误	平均值 95% 置信区间	最小值	最大值	
					下限值	上限			
1.00	40	3.7750	2.00624	.31721	3.1334	4.4166	1.00	7.00	
2.00	40	1.9500	1.08486	.17153	1.6030	2.2970	1.00	4.00	
3.00	40	1.9500	1.08486	.17153	1.6030	2.2970	1.00	4.00	
4.00	40	2.6000	1.03280	.16330	2.2697	2.9303	1.00	4.00	
总计	160	2.5688	1.54440	.12210	2.3276	2.8099	1.00	7.00	


方差同质性检验	
VAR00007  	
Levene 统计	df1	df2	显著性	
13.005	3	156	.000	


ANOVA	
VAR00007  	
	平方和	df	均方	F	显著性	
组之间	88.869	3	29.623	15.915	.000	
组内	290.375	156	1.861			
总计	379.244	159				


事后检验


多重比较	
因变量:   VAR00007  	
	(I) VAR00008	(J) VAR00008	平均差 (I-J)	标准 错误	显著性	95% 置信区间	
						下限值	
LSD(L)	1.00	2.00	1.82500*	.30507	.000	1.2224	
		3.00	1.82500*	.30507	.000	1.2224	
		4.00	1.17500*	.30507	.000	.5724	
	2.00	1.00	-1.82500*	.30507	.000	-2.4276	
		3.00	.00000	.30507	1.000	-.6026	
		4.00	-.65000*	.30507	.035	-1.2526	
	3.00	1.00	-1.82500*	.30507	.000	-2.4276	
		2.00	.00000	.30507	1.000	-.6026	
		4.00	-.65000*	.30507	.035	-1.2526	
	4.00	1.00	-1.17500*	.30507	.000	-1.7776	
		2.00	.65000*	.30507	.035	.0474	
		3.00	.65000*	.30507	.035	.0474	
Tamhane	1.00	2.00	1.82500*	.36062	.000	.8439	
		3.00	1.82500*	.36062	.000	.8439	
		4.00	1.17500*	.35678	.010	.2034	
	2.00	1.00	-1.82500*	.36062	.000	-2.8061	
		3.00	.00000	.24258	1.000	-.6548	
		4.00	-.65000*	.23683	.044	-1.2894	
	3.00	1.00	-1.82500*	.36062	.000	-2.8061	
		2.00	.00000	.24258	1.000	-.6548	
		4.00	-.65000*	.23683	.044	-1.2894	
	4.00	1.00	-1.17500*	.35678	.010	-2.1466	
		2.00	.65000*	.23683	.044	.0106	
		3.00	.65000*	.23683	.044	.0106	

多重比较	
因变量:   VAR00007  	
	(I) VAR00008	(J) VAR00008	95% 置信区间	
			上限	
LSD(L)	1.00	2.00	2.4276	
		3.00	2.4276	
		4.00	1.7776	
	2.00	1.00	-1.2224	
		3.00	.6026	
		4.00	-.0474	
	3.00	1.00	-1.2224	
		2.00	.6026	
		4.00	-.0474	
	4.00	1.00	-.5724	
		2.00	1.2526	
		3.00	1.2526	
Tamhane	1.00	2.00	2.8061	
		3.00	2.8061	
		4.00	2.1466	
	2.00	1.00	-.8439	
		3.00	.6548	
		4.00	-.0106	
	3.00	1.00	-.8439	
		2.00	.6548	
		4.00	-.0106	
	4.00	1.00	-.2034	
		2.00	1.2894	
		3.00	1.2894	

*. 均值差的显著性水平为 0.05。	
