# Supplementary material for: Fecal Microbiota Transplantation Relieves Gastrointestinal and Autism Symptoms by Improving the Gut Microbiota in an Open-Label Study
Source: Front Cell Infect Microbiol. 2021 Oct 19;11:759435. doi: 10.3389/fcimb.2021.759435 (PMC8560686; doi:10.3389/fcimb.2021.759435)
Supplement: Supplementary file 1 [file DataSheet_1.zip › raw data/Figure 2/GSRS/GSRS-Oral statistics.doc]

ONEWAY VAR00004 BY VAR00005
  /STATISTICS DESCRIPTIVES HOMOGENEITY
  /MISSING ANALYSIS
  /POSTHOC=LSD T2 ALPHA(0.05).


Oneway


附注	
已创建输出	12-SEP-2019 22:06:03	
注释		
输入	活动数据集	数据集1	
	过滤器	<无>	
	宽度(W)	<无>	
	拆分文件	<无>	
	工作数据文件中的行数	111	
缺失值处理	缺失定义	用户定义的缺失值视为缺失。	
	使用的个案	每个分析的统计量都基于对于该分析中的任意变量都没有缺失数据的个案。	
语法	ONEWAY VAR00004 BY VAR00005
  /STATISTICS DESCRIPTIVES HOMOGENEITY
  /MISSING ANALYSIS
  /POSTHOC=LSD T2 ALPHA(0.05).	
资源	处理器时间	00:00:00.02	
	用时	00:00:00.03	


描述性	
VAR00004  	
	N	平均值	标准 偏差	标准 错误	平均值 95% 置信区间	最小值	最大值	
					下限值	上限			
1.00	27	59.7407	24.72711	4.75874	49.9590	69.5225	21.00	102.00	
2.00	27	29.0370	9.60161	1.84783	25.2388	32.8353	14.00	46.00	
3.00	27	28.7407	7.20893	1.38736	25.8890	31.5925	18.00	42.00	
4.00	27	34.3704	9.98903	1.92239	30.4188	38.3219	18.00	54.00	
总计	108	37.9722	19.29624	1.85678	34.2914	41.6531	14.00	102.00	


方差同质性检验	
VAR00004  	
Levene 统计	df1	df2	显著性	
27.023	3	104	.000	


ANOVA	
VAR00004  	
	平方和	df	均方	F	显著性	
组之间	17601.287	3	5867.096	27.437	.000	
组内	22239.630	104	213.843			
总计	39840.917	107				


事后检验


多重比较	
因变量:   VAR00004  	
	(I) VAR00005	(J) VAR00005	平均差 (I-J)	标准 错误	显著性	95% 置信区间	
						下限值	
LSD(L)	1.00	2.00	30.70370*	3.97997	.000	22.8113	
		3.00	31.00000*	3.97997	.000	23.1076	
		4.00	25.37037*	3.97997	.000	17.4779	
	2.00	1.00	-30.70370*	3.97997	.000	-38.5961	
		3.00	.29630	3.97997	.941	-7.5961	
		4.00	-5.33333	3.97997	.183	-13.2258	
	3.00	1.00	-31.00000*	3.97997	.000	-38.8924	
		2.00	-.29630	3.97997	.941	-8.1887	
		4.00	-5.62963	3.97997	.160	-13.5221	
	4.00	1.00	-25.37037*	3.97997	.000	-33.2628	
		2.00	5.33333	3.97997	.183	-2.5591	
		3.00	5.62963	3.97997	.160	-2.2628	
Tamhane	1.00	2.00	30.70370*	5.10490	.000	16.4368	
		3.00	31.00000*	4.95685	.000	17.0538	
		4.00	25.37037*	5.13236	.000	11.0423	
	2.00	1.00	-30.70370*	5.10490	.000	-44.9706	
		3.00	.29630	2.31068	1.000	-6.0427	
		4.00	-5.33333	2.66647	.268	-12.6264	
	3.00	1.00	-31.00000*	4.95685	.000	-44.9462	
		2.00	-.29630	2.31068	1.000	-6.6353	
		4.00	-5.62963	2.37073	.123	-12.1388	
	4.00	1.00	-25.37037*	5.13236	.000	-39.6985	
		2.00	5.33333	2.66647	.268	-1.9597	
		3.00	5.62963	2.37073	.123	-.8796	

多重比较	
因变量:   VAR00004  	
	(I) VAR00005	(J) VAR00005	95% 置信区间	
			上限	
LSD(L)	1.00	2.00	38.5961	
		3.00	38.8924	
		4.00	33.2628	
	2.00	1.00	-22.8113	
		3.00	8.1887	
		4.00	2.5591	
	3.00	1.00	-23.1076	
		2.00	7.5961	
		4.00	2.2628	
	4.00	1.00	-17.4779	
		2.00	13.2258	
		3.00	13.5221	
Tamhane	1.00	2.00	44.9706	
		3.00	44.9462	
		4.00	39.6985	
	2.00	1.00	-16.4368	
		3.00	6.6353	
		4.00	1.9597	
	3.00	1.00	-17.0538	
		2.00	6.0427	
		4.00	.8796	
	4.00	1.00	-11.0423	
		2.00	12.6264	
		3.00	12.1388	

*. 均值差的显著性水平为 0.05。	
