# Supplementary material for: Fecal Microbiota Transplantation Relieves Gastrointestinal and Autism Symptoms by Improving the Gut Microbiota in an Open-Label Study
Source: Front Cell Infect Microbiol. 2021 Oct 19;11:759435. doi: 10.3389/fcimb.2021.759435 (PMC8560686; doi:10.3389/fcimb.2021.759435)
Supplement: Supplementary file 1 [file DataSheet_1.zip › raw data/Figure 2/GSRS/GSRS-Rectal statistics.doc]

ONEWAY VAR00006 BY VAR00005
  /STATISTICS DESCRIPTIVES HOMOGENEITY
  /MISSING ANALYSIS
  /POSTHOC=LSD T2 ALPHA(0.05).


Oneway


附注	
已创建输出	12-SEP-2019 22:19:26	
注释		
输入	活动数据集	数据集1	
	过滤器	<无>	
	宽度(W)	<无>	
	拆分文件	<无>	
	工作数据文件中的行数	111	
缺失值处理	缺失定义	用户定义的缺失值视为缺失。	
	使用的个案	每个分析的统计量都基于对于该分析中的任意变量都没有缺失数据的个案。	
语法	ONEWAY VAR00006 BY VAR00005
  /STATISTICS DESCRIPTIVES HOMOGENEITY
  /MISSING ANALYSIS
  /POSTHOC=LSD T2 ALPHA(0.05).	
资源	处理器时间	00:00:00.00	
	用时	00:00:00.01	


描述性	
VAR00006  	
	N	平均值	标准 偏差	标准 错误	平均值 95% 置信区间	最小值	最大值	
					下限值	上限			
1.00	13	69.3846	21.05366	5.83923	56.6620	82.1072	35.00	97.00	
2.00	13	30.1538	8.34512	2.31452	25.1109	35.1968	17.00	43.00	
3.00	13	30.3077	7.35283	2.03931	25.8644	34.7510	19.00	43.00	
4.00	13	35.4615	8.62762	2.39287	30.2479	40.6752	16.00	45.00	
总计	52	41.3269	20.57003	2.85255	35.6002	47.0537	16.00	97.00	


方差同质性检验	
VAR00006  	
Levene 统计	df1	df2	显著性	
7.543	3	48	.000	


ANOVA	
VAR00006  	
	平方和	df	均方	F	显著性	
组之间	13882.673	3	4627.558	28.859	.000	
组内	7696.769	48	160.349			
总计	21579.442	51				


事后检验


多重比较	
因变量:   VAR00006  	
	(I) VAR00005	(J) VAR00005	平均差 (I-J)	标准 错误	显著性	95% 置信区间	
						下限值	
LSD(L)	1.00	2.00	39.23077*	4.96680	.000	29.2443	
		3.00	39.07692*	4.96680	.000	29.0905	
		4.00	33.92308*	4.96680	.000	23.9367	
	2.00	1.00	-39.23077*	4.96680	.000	-49.2172	
		3.00	-.15385	4.96680	.975	-10.1403	
		4.00	-5.30769	4.96680	.291	-15.2941	
	3.00	1.00	-39.07692*	4.96680	.000	-49.0633	
		2.00	.15385	4.96680	.975	-9.8326	
		4.00	-5.15385	4.96680	.305	-15.1403	
	4.00	1.00	-33.92308*	4.96680	.000	-43.9095	
		2.00	5.30769	4.96680	.291	-4.6787	
		3.00	5.15385	4.96680	.305	-4.8326	
Tamhane	1.00	2.00	39.23077*	6.28121	.000	20.3458	
		3.00	39.07692*	6.18510	.000	20.3402	
		4.00	33.92308*	6.31051	.000	14.9902	
	2.00	1.00	-39.23077*	6.28121	.000	-58.1157	
		3.00	-.15385	3.08477	1.000	-9.0073	
		4.00	-5.30769	3.32909	.548	-14.8499	
	3.00	1.00	-39.07692*	6.18510	.000	-57.8136	
		2.00	.15385	3.08477	1.000	-8.6996	
		4.00	-5.15385	3.14398	.518	-14.1846	
	4.00	1.00	-33.92308*	6.31051	.000	-52.8560	
		2.00	5.30769	3.32909	.548	-4.2345	
		3.00	5.15385	3.14398	.518	-3.8770	

多重比较	
因变量:   VAR00006  	
	(I) VAR00005	(J) VAR00005	95% 置信区间	
			上限	
LSD(L)	1.00	2.00	49.2172	
		3.00	49.0633	
		4.00	43.9095	
	2.00	1.00	-29.2443	
		3.00	9.8326	
		4.00	4.6787	
	3.00	1.00	-29.0905	
		2.00	10.1403	
		4.00	4.8326	
	4.00	1.00	-23.9367	
		2.00	15.2941	
		3.00	15.1403	
Tamhane	1.00	2.00	58.1157	
		3.00	57.8136	
		4.00	52.8560	
	2.00	1.00	-20.3458	
		3.00	8.6996	
		4.00	4.2345	
	3.00	1.00	-20.3402	
		2.00	9.0073	
		4.00	3.8770	
	4.00	1.00	-14.9902	
		2.00	14.8499	
		3.00	14.1846	

*. 均值差的显著性水平为 0.05。	
