# Supplementary material for: Fecal Microbiota Transplantation Relieves Gastrointestinal and Autism Symptoms by Improving the Gut Microbiota in an Open-Label Study
Source: Front Cell Infect Microbiol. 2021 Oct 19;11:759435. doi: 10.3389/fcimb.2021.759435 (PMC8560686; doi:10.3389/fcimb.2021.759435)
Supplement: Supplementary file 1 [file DataSheet_1.zip › raw data/Figure 3/5-HT/5-HT control vs week 0.doc]

ONEWAY VAR00001 BY VAR00002
  /STATISTICS DESCRIPTIVES HOMOGENEITY
  /MISSING ANALYSIS
  /POSTHOC=LSD T2 ALPHA(0.05).


Oneway


附注	
已创建输出	14-SEP-2019 21:49:04	
注释		
输入	过滤器	<无>	
	宽度(W)	<无>	
	拆分文件	<无>	
	工作数据文件中的行数	56	
缺失值处理	缺失定义	用户定义的缺失值视为缺失。	
	使用的个案	每个分析的统计量都基于对于该分析中的任意变量都没有缺失数据的个案。	
语法	ONEWAY VAR00001 BY VAR00002
  /STATISTICS DESCRIPTIVES HOMOGENEITY
  /MISSING ANALYSIS
  /POSTHOC=LSD T2 ALPHA(0.05).	
资源	处理器时间	00:00:00.00	
	用时	00:00:00.02	


描述性	
VAR00001  	
	N	平均值	标准 偏差	标准 错误	平均值 95% 置信区间	最小值	最大值	
					下限值	上限			
1.00	9	44.7881	5.75084	1.91695	40.3676	49.2086	38.34	56.78	
2.00	21	67.1575	2.55258	.55702	65.9956	68.3194	63.30	72.59	
3.00	12	64.8926	1.28904	.37211	64.0736	65.7116	62.91	66.69	
总计	42	61.7169	9.54456	1.47276	58.7426	64.6912	38.34	72.59	


方差同质性检验	
VAR00001  	
Levene 统计	df1	df2	显著性	
7.899	2	39	.001	


ANOVA	
VAR00001  	
	平方和	df	均方	F	显著性	
组之间	3321.877	2	1660.938	156.780	.000	
组内	413.169	39	10.594			
总计	3735.045	41				


事后检验


多重比较	
因变量:   VAR00001  	
	(I) VAR00002	(J) VAR00002	平均差 (I-J)	标准 错误	显著性	95% 置信区间	
						下限值	
LSD(L)	1.00	2.00	-22.36939*	1.29676	.000	-24.9923	
		3.00	-20.10446*	1.43526	.000	-23.0075	
	2.00	1.00	22.36939*	1.29676	.000	19.7464	
		3.00	2.26492	1.17784	.062	-.1175	
	3.00	1.00	20.10446*	1.43526	.000	17.2014	
		2.00	-2.26492	1.17784	.062	-4.6473	
Tamhane	1.00	2.00	-22.36939*	1.99624	.000	-28.1527	
		3.00	-20.10446*	1.95273	.000	-25.8697	
	2.00	1.00	22.36939*	1.99624	.000	16.5861	
		3.00	2.26492*	.66988	.006	.5735	
	3.00	1.00	20.10446*	1.95273	.000	14.3392	
		2.00	-2.26492*	.66988	.006	-3.9564	

多重比较	
因变量:   VAR00001  	
	(I) VAR00002	(J) VAR00002	95% 置信区间	
			上限	
LSD(L)	1.00	2.00	-19.7464	
		3.00	-17.2014	
	2.00	1.00	24.9923	
		3.00	4.6473	
	3.00	1.00	23.0075	
		2.00	.1175	
Tamhane	1.00	2.00	-16.5861	
		3.00	-14.3392	
	2.00	1.00	28.1527	
		3.00	3.9564	
	3.00	1.00	25.8697	
		2.00	-.5735	

*. 均值差的显著性水平为 0.05。	
