# Supplementary material for: Fecal Microbiota Transplantation Relieves Gastrointestinal and Autism Symptoms by Improving the Gut Microbiota in an Open-Label Study
Source: Front Cell Infect Microbiol. 2021 Oct 19;11:759435. doi: 10.3389/fcimb.2021.759435 (PMC8560686; doi:10.3389/fcimb.2021.759435)
Supplement: Supplementary file 1 [file DataSheet_1.zip › raw data/Figure 3/5-HT/5-HT Oral-week 0, 4, 8, 12.doc]

ONEWAY VAR00001 BY VAR00002
  /STATISTICS DESCRIPTIVES HOMOGENEITY
  /MISSING ANALYSIS
  /POSTHOC=LSD T2 ALPHA(0.05).


Oneway


附注	
已创建输出	14-SEP-2019 21:58:05	
注释		
输入	过滤器	<无>	
	宽度(W)	<无>	
	拆分文件	<无>	
	工作数据文件中的行数	84	
缺失值处理	缺失定义	用户定义的缺失值视为缺失。	
	使用的个案	每个分析的统计量都基于对于该分析中的任意变量都没有缺失数据的个案。	
语法	ONEWAY VAR00001 BY VAR00002
  /STATISTICS DESCRIPTIVES HOMOGENEITY
  /MISSING ANALYSIS
  /POSTHOC=LSD T2 ALPHA(0.05).	
资源	处理器时间	00:00:00.02	
	用时	00:00:00.02	


描述性	
VAR00001  	
	N	平均值	标准 偏差	标准 错误	平均值 95% 置信区间	最小值	最大值	
					下限值	上限			
1.00	21	67.1575	2.55258	.55702	65.9956	68.3194	63.30	72.59	
2.00	21	47.0387	.97601	.21298	46.5944	47.4830	45.23	48.81	
3.00	21	48.2857	5.00070	1.09124	46.0094	50.5619	27.85	52.29	
4.00	21	55.1729	1.13902	.24855	54.6544	55.6914	53.40	57.60	
总计	84	54.4137	8.52273	.92991	52.5641	56.2632	27.85	72.59	


方差同质性检验	
VAR00001  	
Levene 统计	df1	df2	显著性	
3.206	3	80	.027	


ANOVA	
VAR00001  	
	平方和	df	均方	F	显著性	
组之间	5353.417	3	1784.472	211.351	.000	
组内	675.453	80	8.443			
总计	6028.870	83				


事后检验


多重比较	
因变量:   VAR00001  	
	(I) VAR00002	(J) VAR00002	平均差 (I-J)	标准 错误	显著性	95% 置信区间	
						下限值	
LSD(L)	1.00	2.00	20.11882*	.89672	.000	18.3343	
		3.00	18.87184*	.89672	.000	17.0873	
		4.00	11.98461*	.89672	.000	10.2001	
	2.00	1.00	-20.11882*	.89672	.000	-21.9034	
		3.00	-1.24698	.89672	.168	-3.0315	
		4.00	-8.13420*	.89672	.000	-9.9187	
	3.00	1.00	-18.87184*	.89672	.000	-20.6564	
		2.00	1.24698	.89672	.168	-.5376	
		4.00	-6.88723*	.89672	.000	-8.6718	
	4.00	1.00	-11.98461*	.89672	.000	-13.7691	
		2.00	8.13420*	.89672	.000	6.3497	
		3.00	6.88723*	.89672	.000	5.1027	
Tamhane	1.00	2.00	20.11882*	.59635	.000	18.4198	
		3.00	18.87184*	1.22519	.000	15.4197	
		4.00	11.98461*	.60996	.000	10.2567	
	2.00	1.00	-20.11882*	.59635	.000	-21.8179	
		3.00	-1.24698	1.11183	.854	-4.4663	
		4.00	-8.13420*	.32732	.000	-9.0412	
	3.00	1.00	-18.87184*	1.22519	.000	-22.3239	
		2.00	1.24698	1.11183	.854	-1.9724	
		4.00	-6.88723*	1.11919	.000	-10.1199	
	4.00	1.00	-11.98461*	.60996	.000	-13.7125	
		2.00	8.13420*	.32732	.000	7.2272	
		3.00	6.88723*	1.11919	.000	3.6546	

多重比较	
因变量:   VAR00001  	
	(I) VAR00002	(J) VAR00002	95% 置信区间	
			上限	
LSD(L)	1.00	2.00	21.9034	
		3.00	20.6564	
		4.00	13.7691	
	2.00	1.00	-18.3343	
		3.00	.5376	
		4.00	-6.3497	
	3.00	1.00	-17.0873	
		2.00	3.0315	
		4.00	-5.1027	
	4.00	1.00	-10.2001	
		2.00	9.9187	
		3.00	8.6718	
Tamhane	1.00	2.00	21.8179	
		3.00	22.3239	
		4.00	13.7125	
	2.00	1.00	-18.4198	
		3.00	1.9724	
		4.00	-7.2272	
	3.00	1.00	-15.4197	
		2.00	4.4663	
		4.00	-3.6546	
	4.00	1.00	-10.2567	
		2.00	9.0412	
		3.00	10.1199	

*. 均值差的显著性水平为 0.05。	
