# Supplementary material for: Fecal Microbiota Transplantation Relieves Gastrointestinal and Autism Symptoms by Improving the Gut Microbiota in an Open-Label Study
Source: Front Cell Infect Microbiol. 2021 Oct 19;11:759435. doi: 10.3389/fcimb.2021.759435 (PMC8560686; doi:10.3389/fcimb.2021.759435)
Supplement: Supplementary file 1 [file DataSheet_1.zip › raw data/Figure 3/GABA/GABA Rectal-week 0, 4, 8, 12.doc]

ONEWAY VAR00001 BY VAR00002
  /STATISTICS DESCRIPTIVES HOMOGENEITY
  /MISSING ANALYSIS
  /POSTHOC=LSD T2 ALPHA(0.05).


Oneway


附注	
已创建输出	14-SEP-2019 22:22:08	
注释		
输入	活动数据集	数据集1	
	过滤器	<无>	
	宽度(W)	<无>	
	拆分文件	<无>	
	工作数据文件中的行数	48	
缺失值处理	缺失定义	用户定义的缺失值视为缺失。	
	使用的个案	每个分析的统计量都基于对于该分析中的任意变量都没有缺失数据的个案。	
语法	ONEWAY VAR00001 BY VAR00002
  /STATISTICS DESCRIPTIVES HOMOGENEITY
  /MISSING ANALYSIS
  /POSTHOC=LSD T2 ALPHA(0.05).	
资源	处理器时间	00:00:00.00	
	用时	00:00:00.02	


描述性	
VAR00001  	
	N	平均值	标准 偏差	标准 错误	平均值 95% 置信区间	最小值	最大值	
					下限值	上限			
1.00	12	56.9969	4.94138	1.42645	53.8573	60.1365	49.14	65.91	
2.00	12	45.2643	.48028	.13864	44.9592	45.5695	44.44	45.97	
3.00	12	48.4395	1.41740	.40917	47.5389	49.3401	46.89	51.02	
4.00	12	50.2299	1.62656	.46955	49.1964	51.2633	48.25	53.11	
总计	48	50.2327	5.06591	.73120	48.7617	51.7036	44.44	65.91	


方差同质性检验	
VAR00001  	
Levene 统计	df1	df2	显著性	
9.193	3	44	.000	


ANOVA	
VAR00001  	
	平方和	df	均方	F	显著性	
组之间	883.855	3	294.618	40.217	.000	
组内	322.329	44	7.326			
总计	1206.184	47				


事后检验


多重比较	
因变量:   VAR00001  	
	(I) VAR00002	(J) VAR00002	平均差 (I-J)	标准 错误	显著性	95% 置信区间	
						下限值	
LSD(L)	1.00	2.00	11.73257*	1.10496	.000	9.5057	
		3.00	8.55738*	1.10496	.000	6.3305	
		4.00	6.76704*	1.10496	.000	4.5401	
	2.00	1.00	-11.73257*	1.10496	.000	-13.9595	
		3.00	-3.17518*	1.10496	.006	-5.4021	
		4.00	-4.96552*	1.10496	.000	-7.1924	
	3.00	1.00	-8.55738*	1.10496	.000	-10.7843	
		2.00	3.17518*	1.10496	.006	.9483	
		4.00	-1.79034	1.10496	.112	-4.0172	
	4.00	1.00	-6.76704*	1.10496	.000	-8.9939	
		2.00	4.96552*	1.10496	.000	2.7386	
		3.00	1.79034	1.10496	.112	-.4366	
Tamhane	1.00	2.00	11.73257*	1.43318	.000	7.1696	
		3.00	8.55738*	1.48398	.000	3.9504	
		4.00	6.76704*	1.50175	.003	2.1391	
	2.00	1.00	-11.73257*	1.43318	.000	-16.2955	
		3.00	-3.17518*	.43202	.000	-4.5043	
		4.00	-4.96552*	.48959	.000	-6.4832	
	3.00	1.00	-8.55738*	1.48398	.000	-13.1643	
		2.00	3.17518*	.43202	.000	1.8461	
		4.00	-1.79034	.62281	.052	-3.5931	
	4.00	1.00	-6.76704*	1.50175	.003	-11.3950	
		2.00	4.96552*	.48959	.000	3.4478	
		3.00	1.79034	.62281	.052	-.0124	

多重比较	
因变量:   VAR00001  	
	(I) VAR00002	(J) VAR00002	95% 置信区间	
			上限	
LSD(L)	1.00	2.00	13.9595	
		3.00	10.7843	
		4.00	8.9939	
	2.00	1.00	-9.5057	
		3.00	-.9483	
		4.00	-2.7386	
	3.00	1.00	-6.3305	
		2.00	5.4021	
		4.00	.4366	
	4.00	1.00	-4.5401	
		2.00	7.1924	
		3.00	4.0172	
Tamhane	1.00	2.00	16.2955	
		3.00	13.1643	
		4.00	11.3950	
	2.00	1.00	-7.1696	
		3.00	-1.8461	
		4.00	-3.4478	
	3.00	1.00	-3.9504	
		2.00	4.5043	
		4.00	.0124	
	4.00	1.00	-2.1391	
		2.00	6.4832	
		3.00	3.5931	

*. 均值差的显著性水平为 0.05。	
