# Supplementary material for: Fecal Microbiota Transplantation Relieves Gastrointestinal and Autism Symptoms by Improving the Gut Microbiota in an Open-Label Study
Source: Front Cell Infect Microbiol. 2021 Oct 19;11:759435. doi: 10.3389/fcimb.2021.759435 (PMC8560686; doi:10.3389/fcimb.2021.759435)
Supplement: Supplementary file 1 [file DataSheet_1.zip › raw data/Table 1-3/Table 1 demographics statistics/Carbohydrate consumption▓ε╥∞═│╝╞.doc]

ONEWAY VAR00001 BY VAR00002
  /STATISTICS DESCRIPTIVES HOMOGENEITY
  /MISSING ANALYSIS
  /POSTHOC=LSD T2 ALPHA(0.05).


Oneway


附注	
已创建输出	11-SEP-2019 20:29:23	
注释		
输入	活动数据集	数据集1	
	过滤器	<无>	
	宽度(W)	<无>	
	拆分文件	<无>	
	工作数据文件中的行数	56	
缺失值处理	缺失定义	用户定义的缺失值视为缺失。	
	使用的个案	每个分析的统计量都基于对于该分析中的任意变量都没有缺失数据的个案。	
语法	ONEWAY VAR00001 BY VAR00002
  /STATISTICS DESCRIPTIVES HOMOGENEITY
  /MISSING ANALYSIS
  /POSTHOC=LSD T2 ALPHA(0.05).	
资源	处理器时间	00:00:00.00	
	用时	00:00:00.01	


描述性	
VAR00001  	
	N	平均值	标准 偏差	标准 错误	平均值 95% 置信区间	最小值	
					下限值	上限		
1.00	16	106.5625	17.19678	4.29919	97.3990	115.7260	81.00	
2.00	27	116.1111	17.44295	3.35690	109.2109	123.0113	90.00	
3.00	13	115.6154	13.91365	3.85895	107.2074	124.0233	88.00	
总计	56	113.2679	16.88409	2.25623	108.7463	117.7894	81.00	

描述性	
VAR00001  	
	最大值	
		
1.00	135.00	
2.00	143.00	
3.00	132.00	
总计	143.00	


方差同质性检验	
VAR00001  	
Levene 统计	df1	df2	显著性	
.856	2	53	.431	


ANOVA	
VAR00001  	
	平方和	df	均方	F	显著性	
组之间	1009.301	2	504.651	1.823	.171	
组内	14669.681	53	276.786			
总计	15678.982	55				


事后检验


多重比较	
因变量:   VAR00001  	
	(I) VAR00002	(J) VAR00002	平均差 (I-J)	标准 错误	显著性	95% 置信区间	
						下限值	
LSD(L)	1.00	2.00	-9.54861	5.24886	.075	-20.0765	
		3.00	-9.05288	6.21212	.151	-21.5128	
	2.00	1.00	9.54861	5.24886	.075	-.9793	
		3.00	.49573	5.61628	.930	-10.7691	
	3.00	1.00	9.05288	6.21212	.151	-3.4070	
		2.00	-.49573	5.61628	.930	-11.7606	
Tamhane	1.00	2.00	-9.54861	5.45452	.245	-23.2897	
		3.00	-9.05288	5.77707	.339	-23.7559	
	2.00	1.00	9.54861	5.45452	.245	-4.1925	
		3.00	.49573	5.11471	1.000	-12.4551	
	3.00	1.00	9.05288	5.77707	.339	-5.6502	
		2.00	-.49573	5.11471	1.000	-13.4465	

多重比较	
因变量:   VAR00001  	
	(I) VAR00002	(J) VAR00002	95% 置信区间	
			上限	
LSD(L)	1.00	2.00	.9793	
		3.00	3.4070	
	2.00	1.00	20.0765	
		3.00	11.7606	
	3.00	1.00	21.5128	
		2.00	10.7691	
Tamhane	1.00	2.00	4.1925	
		3.00	5.6502	
	2.00	1.00	23.2897	
		3.00	13.4465	
	3.00	1.00	23.7559	
		2.00	12.4551	
