# Supplementary material for: Fecal Microbiota Transplantation Relieves Gastrointestinal and Autism Symptoms by Improving the Gut Microbiota in an Open-Label Study
Source: Front Cell Infect Microbiol. 2021 Oct 19;11:759435. doi: 10.3389/fcimb.2021.759435 (PMC8560686; doi:10.3389/fcimb.2021.759435)
Supplement: Supplementary file 1 [file DataSheet_1.zip › raw data/Table 1-3/Table 1 demographics statistics/Fat consumption▓ε╥∞═│╝╞.doc]

ONEWAY VAR00001 BY VAR00002
  /STATISTICS DESCRIPTIVES HOMOGENEITY
  /MISSING ANALYSIS
  /POSTHOC=LSD T2 ALPHA(0.05).


Oneway


附注	
已创建输出	11-SEP-2019 20:55:15	
注释		
输入	活动数据集	数据集1	
	过滤器	<无>	
	宽度(W)	<无>	
	拆分文件	<无>	
	工作数据文件中的行数	56	
缺失值处理	缺失定义	用户定义的缺失值视为缺失。	
	使用的个案	每个分析的统计量都基于对于该分析中的任意变量都没有缺失数据的个案。	
语法	ONEWAY VAR00001 BY VAR00002
  /STATISTICS DESCRIPTIVES HOMOGENEITY
  /MISSING ANALYSIS
  /POSTHOC=LSD T2 ALPHA(0.05).	
资源	处理器时间	00:00:00.00	
	用时	00:00:00.01	


描述性	
VAR00001  	
	N	平均值	标准 偏差	标准 错误	平均值 95% 置信区间	最小值	最大值	
					下限值	上限			
1.00	16	54.0625	14.05451	3.51363	46.5734	61.5516	34.00	77.00	
2.00	27	56.0370	16.17805	3.11347	49.6372	62.4369	30.00	81.00	
3.00	13	58.3846	14.47721	4.01526	49.6361	67.1331	30.00	78.00	
总计	56	56.0179	15.02542	2.00786	51.9940	60.0417	30.00	81.00	


方差同质性检验	
VAR00001  	
Levene 统计	df1	df2	显著性	
.444	2	53	.644	


ANOVA	
VAR00001  	
	平方和	df	均方	F	显著性	
组之间	134.005	2	67.002	.289	.750	
组内	12282.977	53	231.754			
总计	12416.982	55				


事后检验


多重比较	
因变量:   VAR00001  	
	(I) VAR00002	(J) VAR00002	平均差 (I-J)	标准 错误	显著性	95% 置信区间	
						下限值	
LSD(L)	1.00	2.00	-1.97454	4.80293	.683	-11.6080	
		3.00	-4.32212	5.68436	.450	-15.7235	
	2.00	1.00	1.97454	4.80293	.683	-7.6589	
		3.00	-2.34758	5.13914	.650	-12.6554	
	3.00	1.00	4.32212	5.68436	.450	-7.0793	
		2.00	2.34758	5.13914	.650	-7.9602	
Tamhane	1.00	2.00	-1.97454	4.69460	.966	-13.7416	
		3.00	-4.32212	5.33553	.810	-17.9552	
	2.00	1.00	1.97454	4.69460	.966	-9.7925	
		3.00	-2.34758	5.08094	.956	-15.2992	
	3.00	1.00	4.32212	5.33553	.810	-9.3109	
		2.00	2.34758	5.08094	.956	-10.6041	

多重比较	
因变量:   VAR00001  	
	(I) VAR00002	(J) VAR00002	95% 置信区间	
			上限	
LSD(L)	1.00	2.00	7.6589	
		3.00	7.0793	
	2.00	1.00	11.6080	
		3.00	7.9602	
	3.00	1.00	15.7235	
		2.00	12.6554	
Tamhane	1.00	2.00	9.7925	
		3.00	9.3109	
	2.00	1.00	13.7416	
		3.00	10.6041	
	3.00	1.00	17.9552	
		2.00	15.2992	
