# Supplementary material for: Fecal Microbiota Transplantation Relieves Gastrointestinal and Autism Symptoms by Improving the Gut Microbiota in an Open-Label Study
Source: Front Cell Infect Microbiol. 2021 Oct 19;11:759435. doi: 10.3389/fcimb.2021.759435 (PMC8560686; doi:10.3389/fcimb.2021.759435)
Supplement: Supplementary file 1 [file DataSheet_1.zip › raw data/Table 1-3/Table 1 demographics statistics/Protein consumption▓ε╥∞═│╝╞.doc]

ONEWAY VAR00001 BY VAR00002
  /STATISTICS DESCRIPTIVES HOMOGENEITY
  /MISSING ANALYSIS
  /POSTHOC=LSD T2 ALPHA(0.05).


Oneway


附注	
已创建输出	11-SEP-2019 21:04:43	
注释		
输入	活动数据集	数据集1	
	过滤器	<无>	
	宽度(W)	<无>	
	拆分文件	<无>	
	工作数据文件中的行数	56	
缺失值处理	缺失定义	用户定义的缺失值视为缺失。	
	使用的个案	每个分析的统计量都基于对于该分析中的任意变量都没有缺失数据的个案。	
语法	ONEWAY VAR00001 BY VAR00002
  /STATISTICS DESCRIPTIVES HOMOGENEITY
  /MISSING ANALYSIS
  /POSTHOC=LSD T2 ALPHA(0.05).	
资源	处理器时间	00:00:00.02	
	用时	00:00:00.01	


描述性	
VAR00001  	
	N	平均值	标准 偏差	标准 错误	平均值 95% 置信区间	最小值	最大值	
					下限值	上限			
1.00	16	45.6875	9.60360	2.40090	40.5701	50.8049	32.00	66.00	
2.00	27	52.4815	11.14716	2.14527	48.0718	56.8912	32.00	67.00	
3.00	13	50.8462	6.60614	1.83221	46.8541	54.8382	40.00	58.00	
总计	56	50.1607	10.09910	1.34955	47.4562	52.8653	32.00	67.00	


方差同质性检验	
VAR00001  	
Levene 统计	df1	df2	显著性	
2.018	2	53	.143	


ANOVA	
VAR00001  	
	平方和	df	均方	F	显著性	
组之间	471.683	2	235.842	2.433	.098	
组内	5137.871	53	96.941			
总计	5609.554	55				


事后检验


多重比较	
因变量:   VAR00001  	
	(I) VAR00002	(J) VAR00002	平均差 (I-J)	标准 错误	显著性	95% 置信区间	
						下限值	
LSD(L)	1.00	2.00	-6.79398*	3.10632	.033	-13.0245	
		3.00	-5.15865	3.67638	.166	-12.5325	
	2.00	1.00	6.79398*	3.10632	.033	.5635	
		3.00	1.63533	3.32376	.625	-5.0313	
	3.00	1.00	5.15865	3.67638	.166	-2.2152	
		2.00	-1.63533	3.32376	.625	-8.3020	
Tamhane	1.00	2.00	-6.79398	3.21971	.121	-14.8618	
		3.00	-5.15865	3.02015	.269	-12.8571	
	2.00	1.00	6.79398	3.21971	.121	-1.2739	
		3.00	1.63533	2.82120	.918	-5.4277	
	3.00	1.00	5.15865	3.02015	.269	-2.5398	
		2.00	-1.63533	2.82120	.918	-8.6983	

多重比较	
因变量:   VAR00001  	
	(I) VAR00002	(J) VAR00002	95% 置信区间	
			上限	
LSD(L)	1.00	2.00	-.5635	
		3.00	2.2152	
	2.00	1.00	13.0245	
		3.00	8.3020	
	3.00	1.00	12.5325	
		2.00	5.0313	
Tamhane	1.00	2.00	1.2739	
		3.00	2.5398	
	2.00	1.00	14.8618	
		3.00	8.6983	
	3.00	1.00	12.8571	
		2.00	5.4277	

*. 均值差的显著性水平为 0.05。	
