# Supplementary material for: Fecal Microbiota Transplantation Relieves Gastrointestinal and Autism Symptoms by Improving the Gut Microbiota in an Open-Label Study
Source: Front Cell Infect Microbiol. 2021 Oct 19;11:759435. doi: 10.3389/fcimb.2021.759435 (PMC8560686; doi:10.3389/fcimb.2021.759435)
Supplement: Supplementary file 1 [file DataSheet_1.zip › raw data/Table 1-3/Table 2 bristol score statistics/Abnormal - Oral 0 vs 12.doc]

CROSSTABS
  /TABLES=VAR00001 BY VAR00002
  /FORMAT=AVALUE TABLES
  /STATISTICS=CHISQ
  /CELLS=COUNT EXPECTED ROW
  /COUNT ROUND CELL.


交叉表格


附注	
已创建输出	12-SEP-2019 10:40:24	
注释		
输入	活动数据集	数据集1	
	过滤器	<无>	
	宽度(W)	VAR00003	
	拆分文件	<无>	
	工作数据文件中的行数	4	
缺失值处理	对缺失的定义	用户定义的缺失值被视作缺失。	
	已使用的个案	每个表格的统计信息是基于指定范围内每个表格的所有变量中带有有效数据的所有个案。	
语法	CROSSTABS
  /TABLES=VAR00001 BY VAR00002
  /FORMAT=AVALUE TABLES
  /STATISTICS=CHISQ
  /CELLS=COUNT EXPECTED ROW
  /COUNT ROUND CELL.	
资源	处理器时间	00:00:00.00	
	用时	00:00:00.01	
	请求的维数	2	
	可用单元格	131029	


个案处理摘要	
	个案	
	有效	缺失	总计	
	数字	百分比	数字	百分比	数字	百分比	
VAR00001 * VAR00002	54	100.0%	0	0.0%	54	100.0%	


VAR00001 * VAR00002 交叉表	
	VAR00002	总计	
	1.00	2.00		
VAR00001	1.00	计数	23	4	27	
		预期计数	14.5	12.5	27.0	
		百分比在 VAR00001 内	85.2%	14.8%	100.0%	
	2.00	计数	6	21	27	
		预期计数	14.5	12.5	27.0	
		百分比在 VAR00001 内	22.2%	77.8%	100.0%	
总计	计数	29	25	54	
	预期计数	29.0	25.0	54.0	
	百分比在 VAR00001 内	53.7%	46.3%	100.0%	


卡方检验	
	值	自由度	渐近显著性 （双向）	精确显著性（双向）	精确显著性（单向）	
皮尔逊卡方	21.526a	1	.000			
连续校正b	19.068	1	.000			
似然比(L)	23.307	1	.000			
Fisher 精确检验				.000	.000	
线性关联	21.127	1	.000			
有效个案数	54					

a. 0 个单元格 (0.0%) 具有的预期计数少于 5。最小预期计数为 12.50。	
b. 仅为 2x2 表格计算	


警告号 3211
至少在一个个案中，weight 变量的值为零、负数或缺少该值。这些个案对需要正值 加权个案的统计过程和图形不可见，但仍然保留在文件中，并且由非统计设施（例如
LIST 和 SAVE）处理。
