# Supplementary material for: Fecal Microbiota Transplantation Relieves Gastrointestinal and Autism Symptoms by Improving the Gut Microbiota in an Open-Label Study
Source: Front Cell Infect Microbiol. 2021 Oct 19;11:759435. doi: 10.3389/fcimb.2021.759435 (PMC8560686; doi:10.3389/fcimb.2021.759435)
Supplement: Supplementary file 1 [file DataSheet_1.zip › raw data/Table 1-3/Table 2 bristol score statistics/No stool - Rectal 0 vs 12.doc]

CROSSTABS
  /TABLES=VAR00001 BY VAR00002
  /FORMAT=AVALUE TABLES
  /STATISTICS=CHISQ
  /CELLS=COUNT EXPECTED ROW
  /COUNT ROUND CELL.


交叉表格


附注	
已创建输出	12-SEP-2019 10:15:38	
注释		
输入	活动数据集	数据集1	
	过滤器	<无>	
	宽度(W)	VAR00003	
	拆分文件	<无>	
	工作数据文件中的行数	2	
缺失值处理	对缺失的定义	用户定义的缺失值被视作缺失。	
	已使用的个案	每个表格的统计信息是基于指定范围内每个表格的所有变量中带有有效数据的所有个案。	
语法	CROSSTABS
  /TABLES=VAR00001 BY VAR00002
  /FORMAT=AVALUE TABLES
  /STATISTICS=CHISQ
  /CELLS=COUNT EXPECTED ROW
  /COUNT ROUND CELL.	
资源	处理器时间	00:00:00.02	
	用时	00:00:00.01	
	请求的维数	2	
	可用单元格	131029	


警告	
没有为 VAR00001 * VAR00002 的交叉表计算关联的度量。双向表格中至少一个变量（有关已计算的关联的度量）是常量。	


个案处理摘要	
	个案	
	有效	缺失	总计	
	数字	百分比	数字	百分比	数字	百分比	
VAR00001 * VAR00002	54	100.0%	0	0.0%	54	100.0%	


VAR00001 * VAR00002 交叉表	
	VAR00002	总计	
	2.00		
VAR00001	1.00	计数	27	27	
		预期计数	27.0	27.0	
		百分比在 VAR00001 内	100.0%	100.0%	
	2.00	计数	27	27	
		预期计数	27.0	27.0	
		百分比在 VAR00001 内	100.0%	100.0%	
总计	计数	54	54	
	预期计数	54.0	54.0	
	百分比在 VAR00001 内	100.0%	100.0%	


卡方检验	
	值	
皮尔逊卡方	.a	
有效个案数	54	

a. 没有计算统计信息，因为 VAR00002 是常量。	


警告号 3211
至少在一个个案中，weight 变量的值为零、负数或缺少该值。这些个案对需要正值 加权个案的统计过程和图形不可见，但仍然保留在文件中，并且由非统计设施（例如
LIST 和 SAVE）处理。
