# Supplementary material for: Fecal Microbiota Transplantation Relieves Gastrointestinal and Autism Symptoms by Improving the Gut Microbiota in an Open-Label Study
Source: Front Cell Infect Microbiol. 2021 Oct 19;11:759435. doi: 10.3389/fcimb.2021.759435 (PMC8560686; doi:10.3389/fcimb.2021.759435)
Supplement: Supplementary file 1 [file DataSheet_1.zip › raw data/Table 1-3/Table1-3.docx]

**Table 1** Demographic Characteristics of study participants and their medical history. All values are mean ± standard deviation (SD). p-value is calculate by one-way anova analysis. ns : not-significant.

| Category | Neurotypical  population  n =16 | Oral population  n = 27 | Rectal population  n = 13 | F-value  (two-tailed) |
| --- | --- | --- | --- | --- |
| Demographic characteristics | | | | |
| Age, (years), mean (SD) | 7.13±3.20 | 9.15±4.10 | 5.76±1.50 | 0.013 |
| Gender, n (%) |  |  |  |  |
| Female | 1 | 2 | 1 |  |
| Male | 15 | 25 | 12 |  |
| BMI (kg/m2), mean (SD) | 16.90±2.52 | 18.77±4.61 | 16.27±1.60 | ns, P=0.081 |
| Autim severity, n (%) |  |  |  |  |
| Mild |  | 40.74% | 23.08% |  |
| Moderate |  | 37.04% | 30.77% |  |
| Severe |  | 22.22% | 46.15% |  |
| Food allergy (moderate or severe) | 6.25% | 59.26% | 61.54% | P<0.001 |
| Oral antibiotic use during first 4 years of life (number of rounds) | 5.75±1.86 | 10.27±3.41 | 9.62±3.82 | P<0.001 |
| Carbohydrate consumption (g) | 106.56±17.20 | 116.11±17.44 | 115.62±13.91 | ns, P=0.171 |
| Fat consumption (g) | 54.06±14.05 | 56.04±16.18 | 58.38±14.48 | ns, P=0.750 |
| Protein consumption (g) | 48.44±11.36 | 52.48±11.15 | 50.85±6.67 | ns, P=0.098 |

**Table 2** The percent of stool style based on the daily stool record and the Bristol Stool Form Scale (p-valve by two-tailed χ^2^ test).

| Category | Week  0 | | Week  4 | | Week  12 | | 1. valve (week 0 vs.) | | | |  |  |
| --- | --- | --- | --- | --- | --- | --- | --- | --- | --- | --- | --- | --- |
|  |  |  |  |  |  |  | week 4 | | week 12 | | |  |
|  | Oral  (%) | Rectal  (%) | Oral  (%) | Rectal  (%) | Oral  (%) | Rectal  (%) | Oral | Rectal | Oral | Rectal | | |
| No stool | 7.4 | 0 | 0 | 0 | 3.7 | 0 | 0.491 | 1.000 | 1.000 | 1.000 | | |
| Hard stool  (type 1 or 2) | 70.4 | 61.5 | 18.5 | 15.4 | 18.5 | 15.4 | 0.000 | 0.041 | 0.000 | 0.041 | | |
| Soft/liquid stool  (type 6 or 7) | 7.4 | 15.4 | 0 | 0 | 0 | 0 | 0.491 | 0.480 | 0.491 | 0.480 | | |
| Abnormal stool  ( in total of hard, soft/liquid/ and no stool) | 85.2 | 76.9 | 18.5 | 23.1 | 22.2 | 30.8 | 0.000 | 0.017 | 0.000 | 0.047 | | |

**Table 3** Adverse effects of oral and rectal administration.

| Adverse effect | % adverse effects | |
| --- | --- | --- |
|  | Oral | Rectal |
| Rash | 0% | 0% |
| Fever | 3.7% | 0% |
| Hyperactivity | 3.7% | 7.7% |
| Tantrums/aggression | 3.7% | 0% |
| Nausea/vomiting | 0% | 0% |
