# Supplementary figures and images for: Fecal Microbiota Transplantation Relieves Gastrointestinal and Autism Symptoms by Improving the Gut Microbiota in an Open-Label Study
Source: Front Cell Infect Microbiol. 2021 Oct 19;11:759435. doi: 10.3389/fcimb.2021.759435 (PMC8560686; doi:10.3389/fcimb.2021.759435)

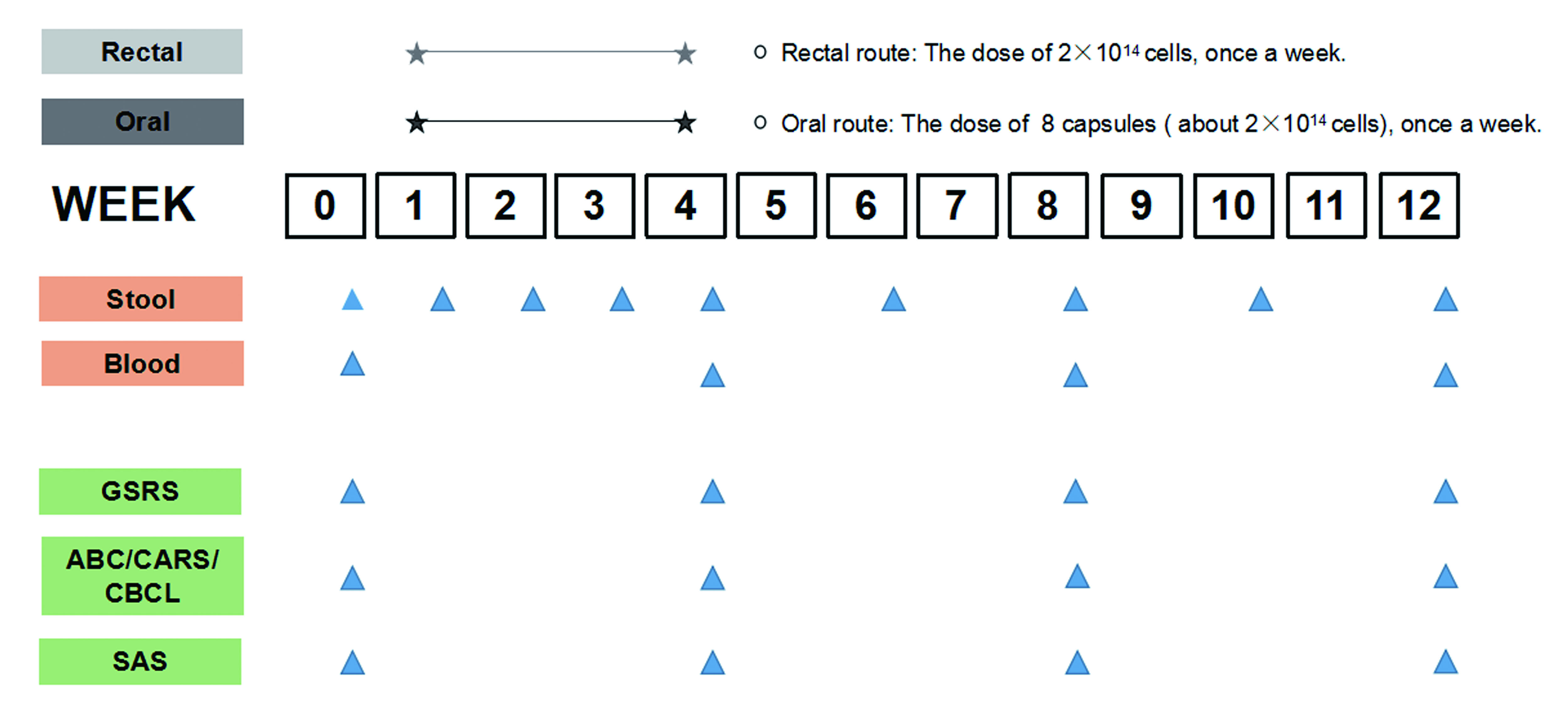

Supplement: Supplementary file 1 [file DataSheet_1.zip › raw data/Figure 1/figure 1.jpg]

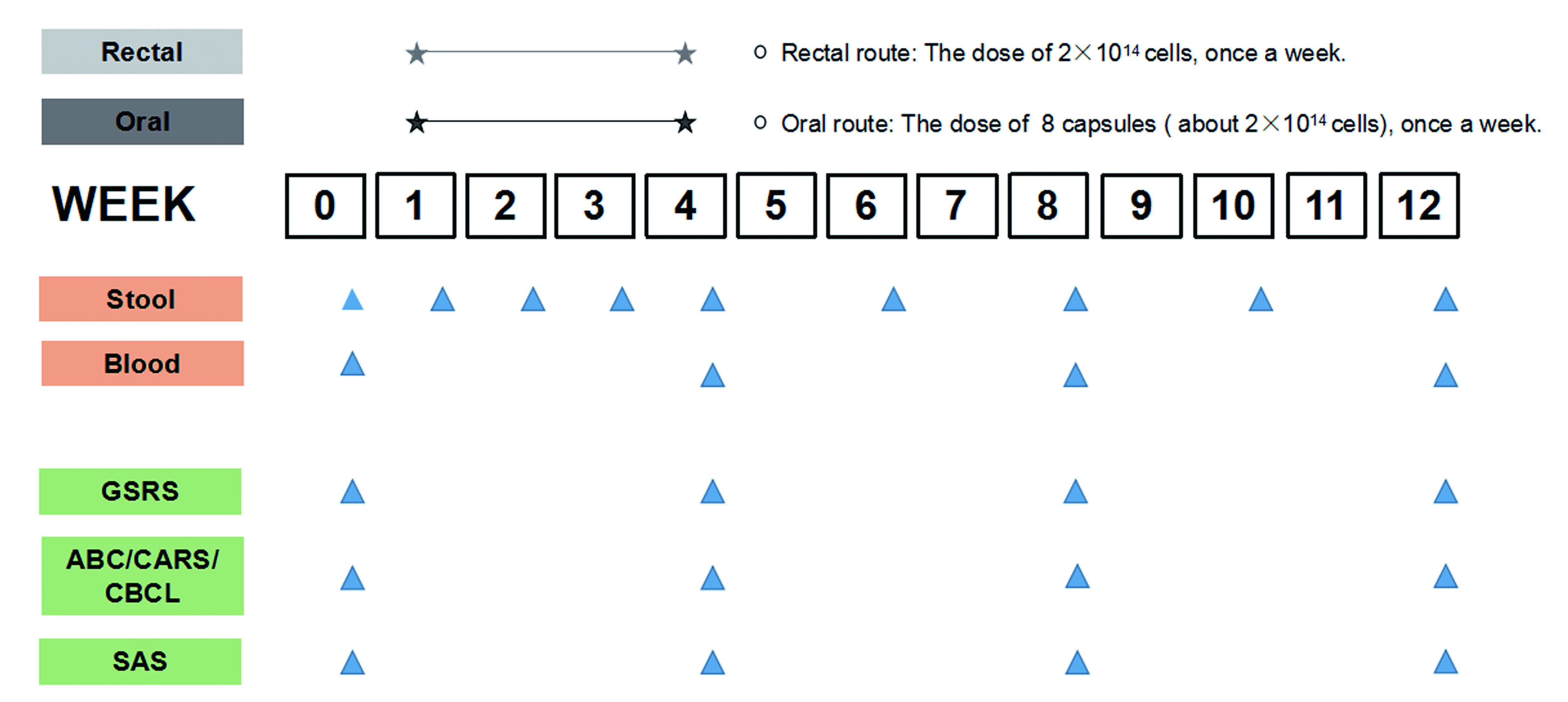

Supplement: Supplementary file 1 [file DataSheet_1.zip › raw data/Figure 1/figure 1.tif]

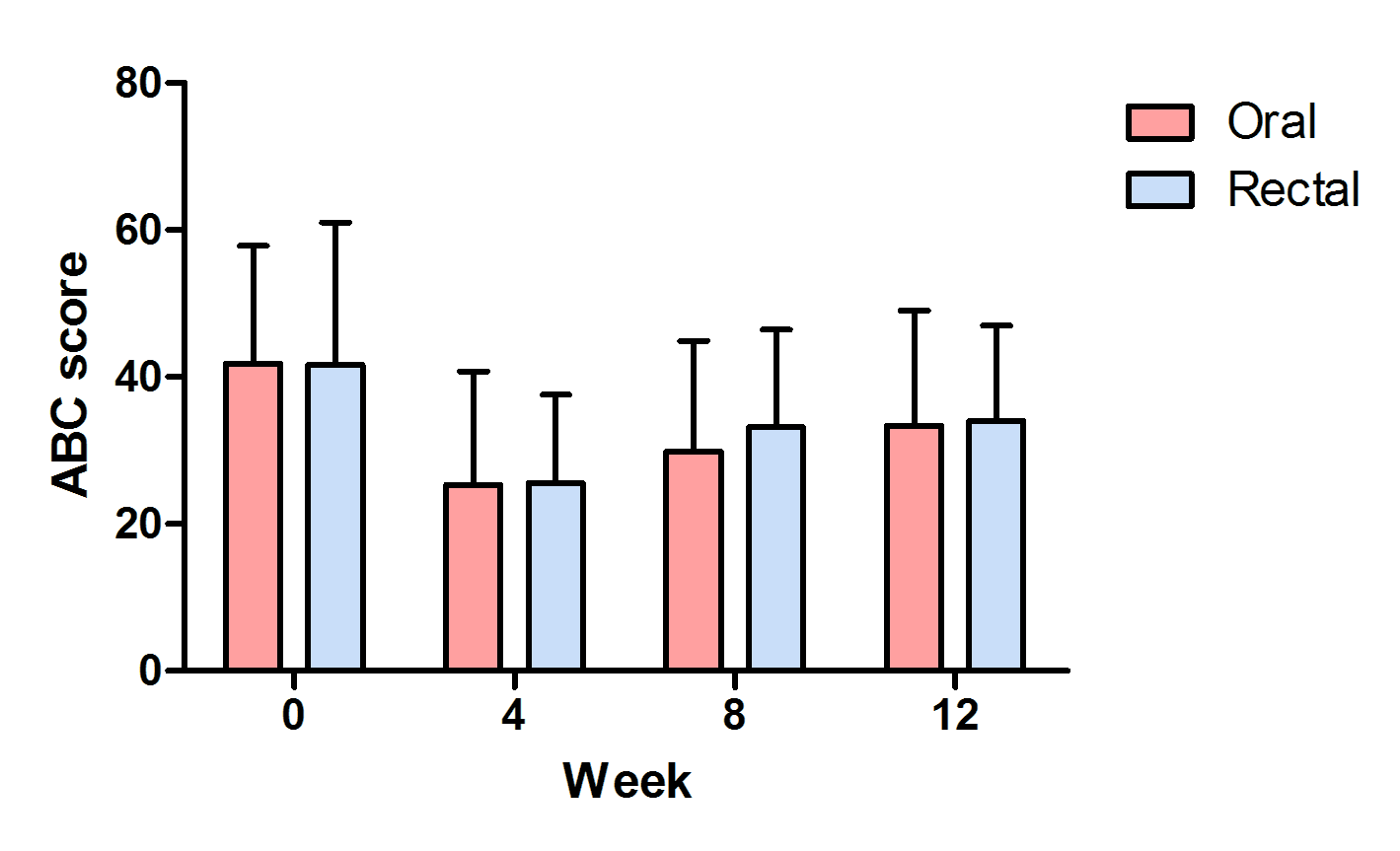

Supplement: Supplementary file 1 [file DataSheet_1.zip › raw data/Figure 2/CARS+CBCL+ABC+SAS/ABC.tif]

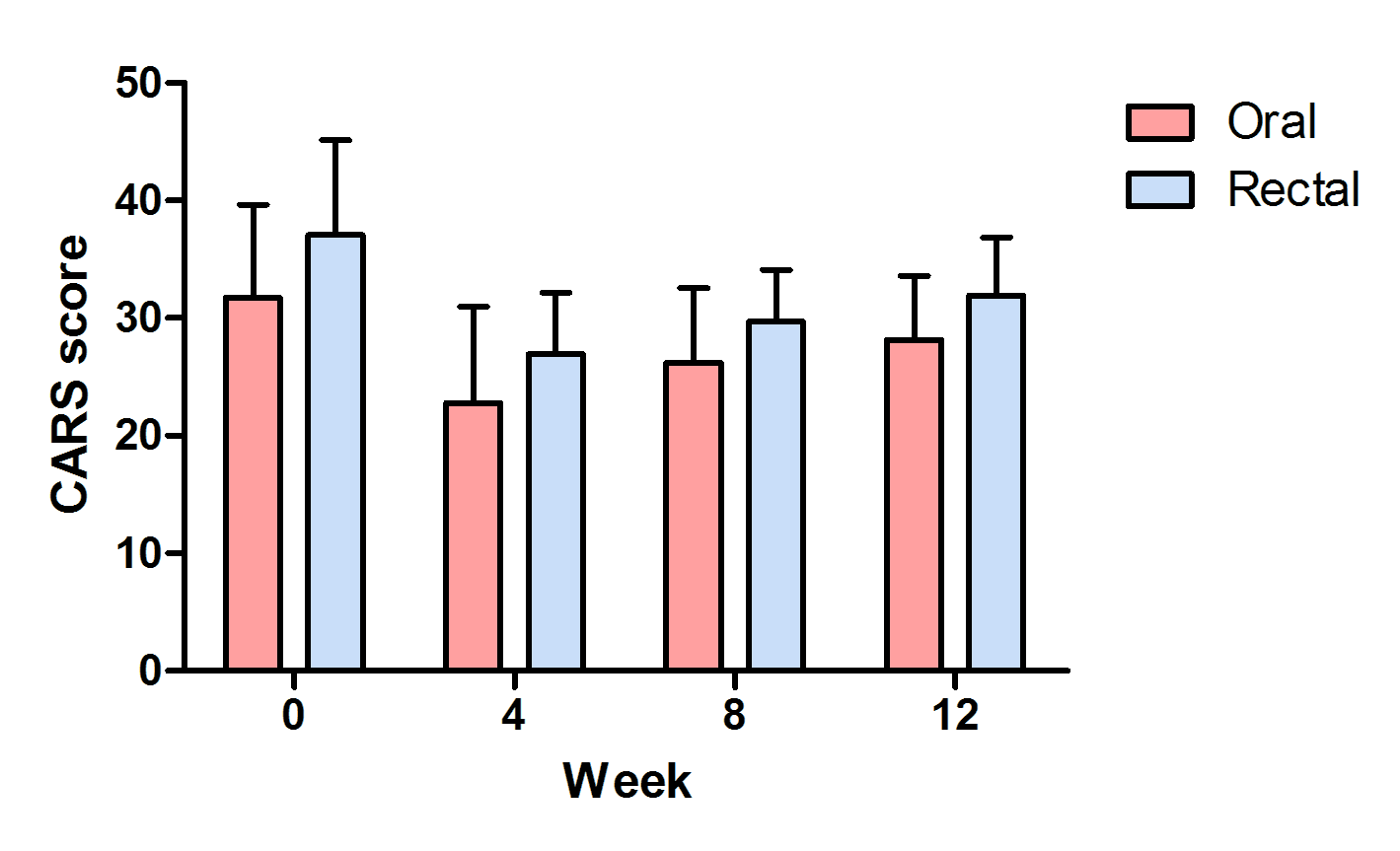

Supplement: Supplementary file 1 [file DataSheet_1.zip › raw data/Figure 2/CARS+CBCL+ABC+SAS/CARS.tif]

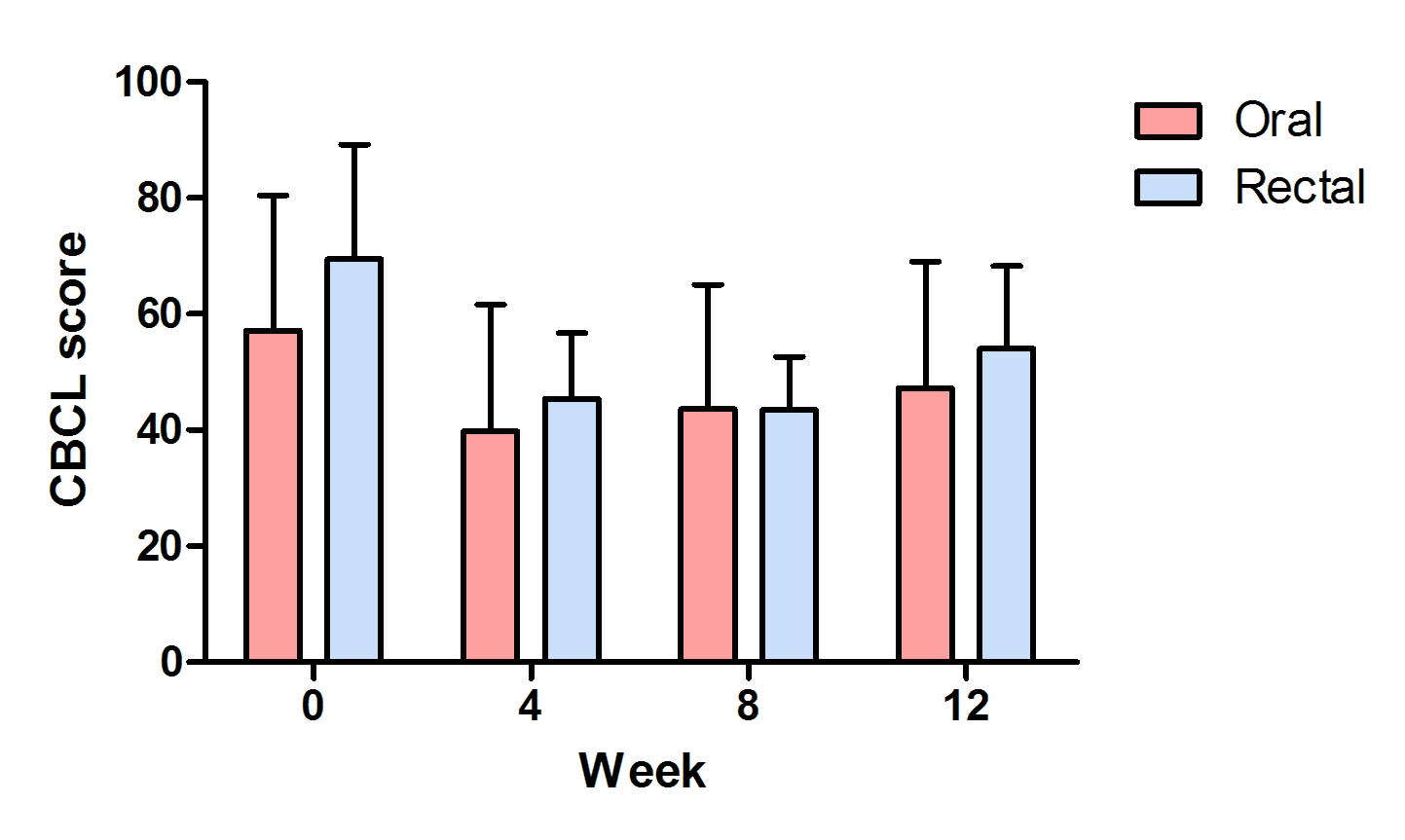

Supplement: Supplementary file 1 [file DataSheet_1.zip › raw data/Figure 2/CARS+CBCL+ABC+SAS/CBCL.tif]

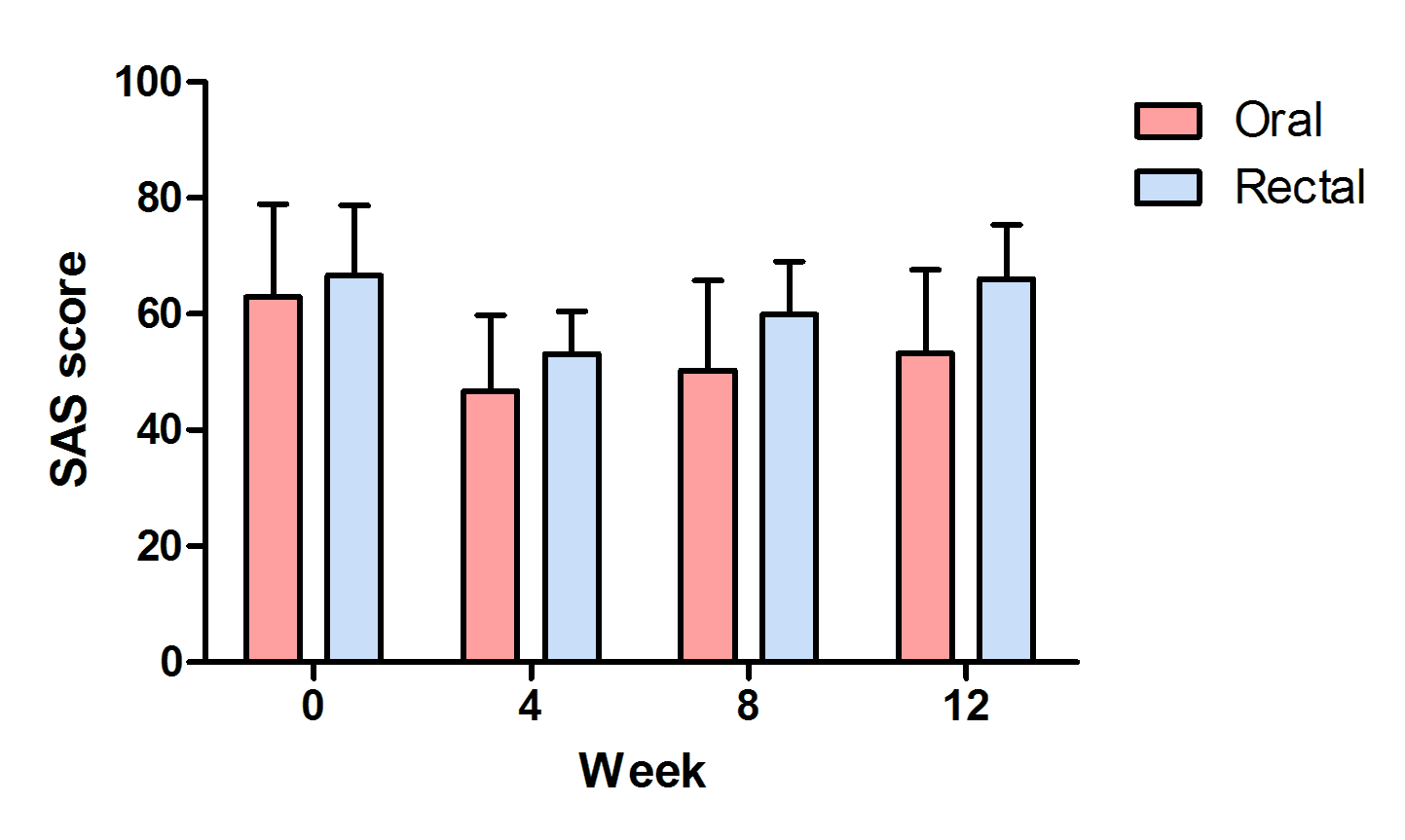

Supplement: Supplementary file 1 [file DataSheet_1.zip › raw data/Figure 2/CARS+CBCL+ABC+SAS/SAS.tif]

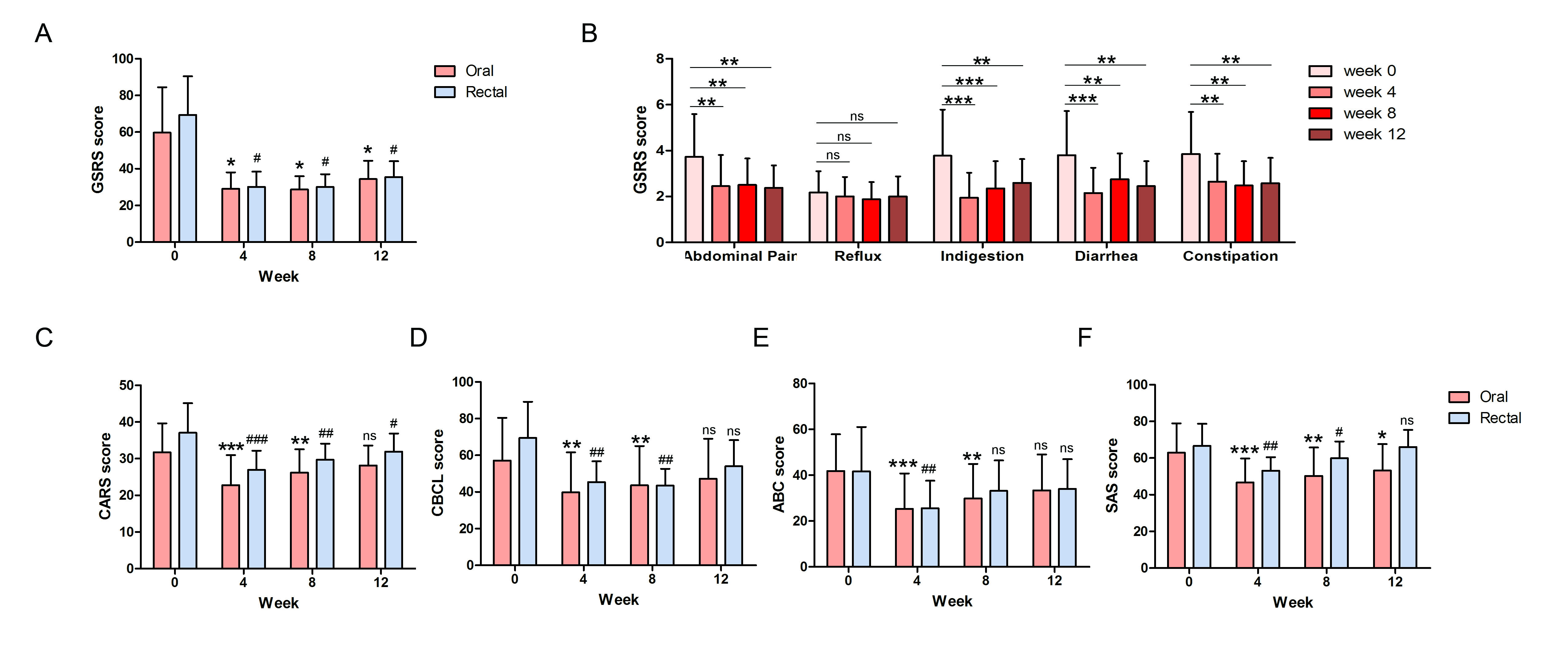

Supplement: Supplementary file 1 [file DataSheet_1.zip › raw data/Figure 2/figure 2-1.jpg]

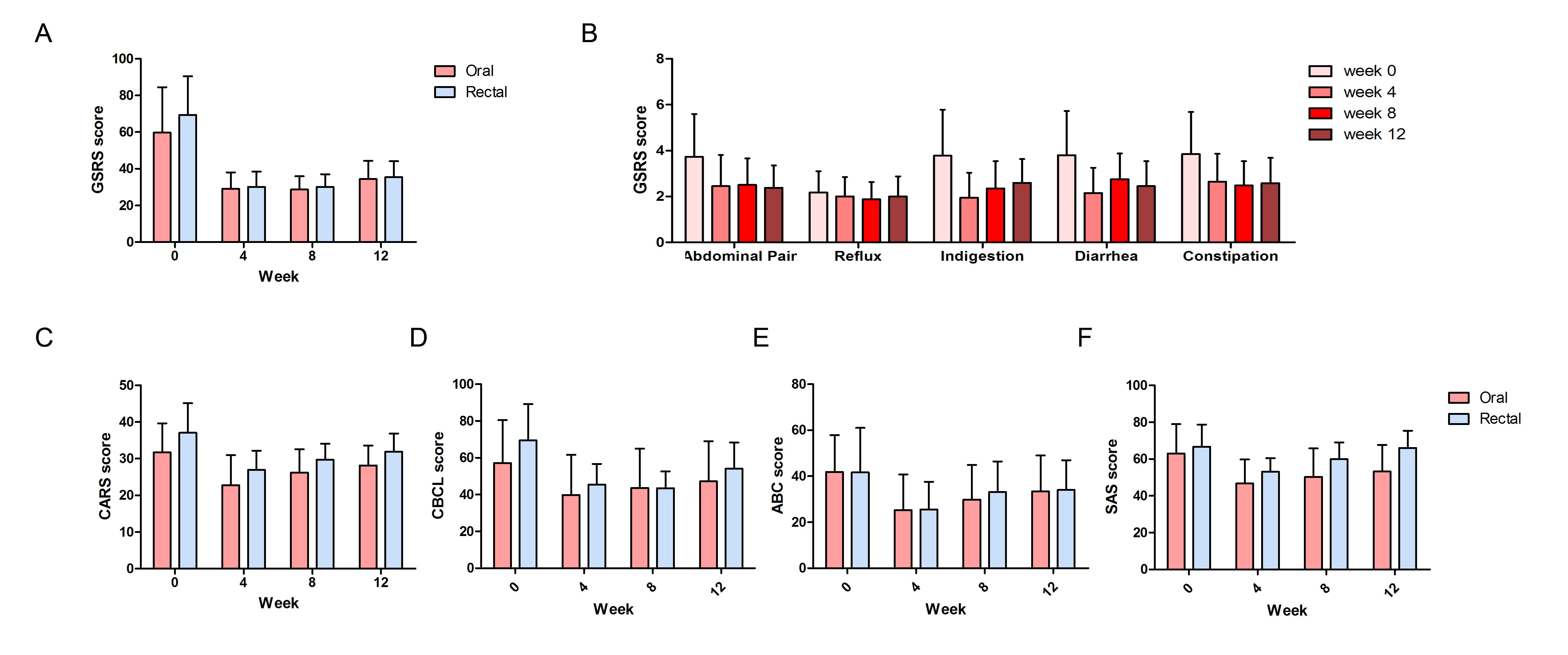

Supplement: Supplementary file 1 [file DataSheet_1.zip › raw data/Figure 2/figure 2.jpg]

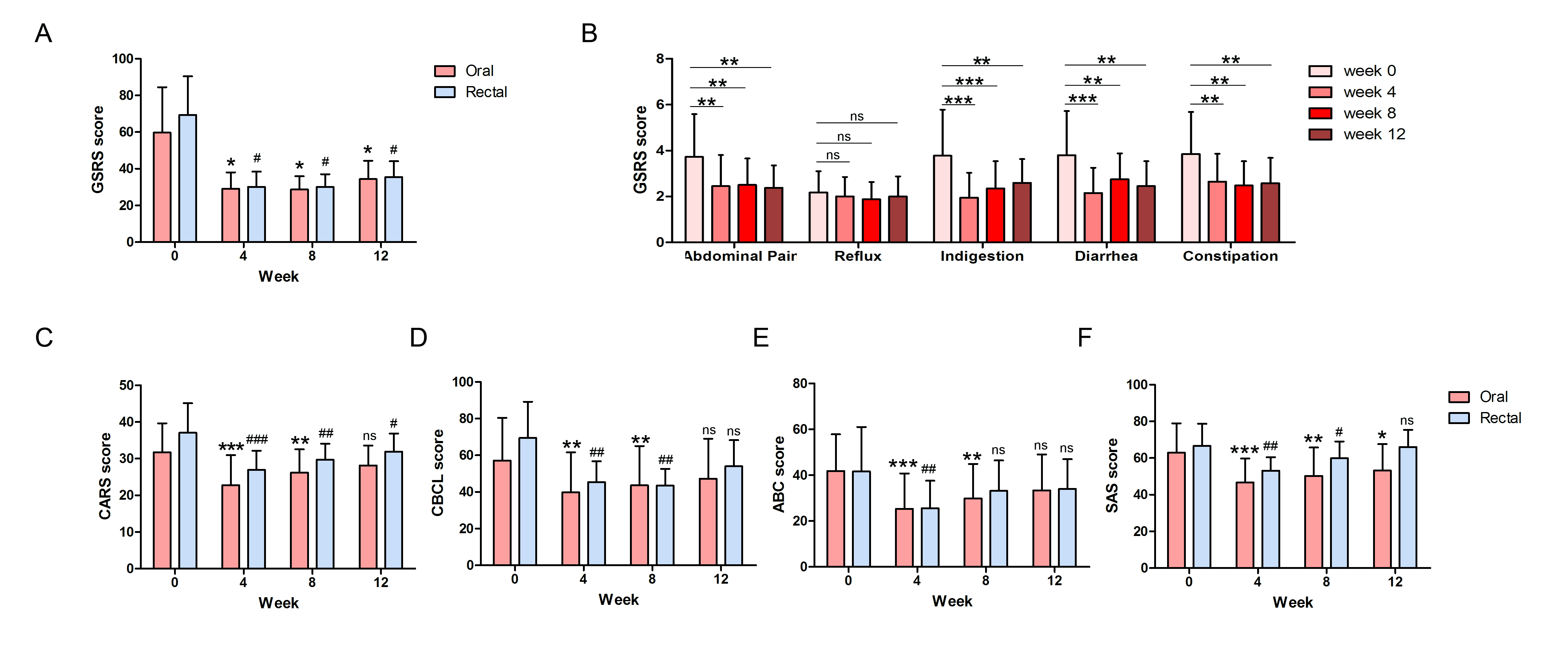

Supplement: Supplementary file 1 [file DataSheet_1.zip › raw data/Figure 2/figure 2.tif]

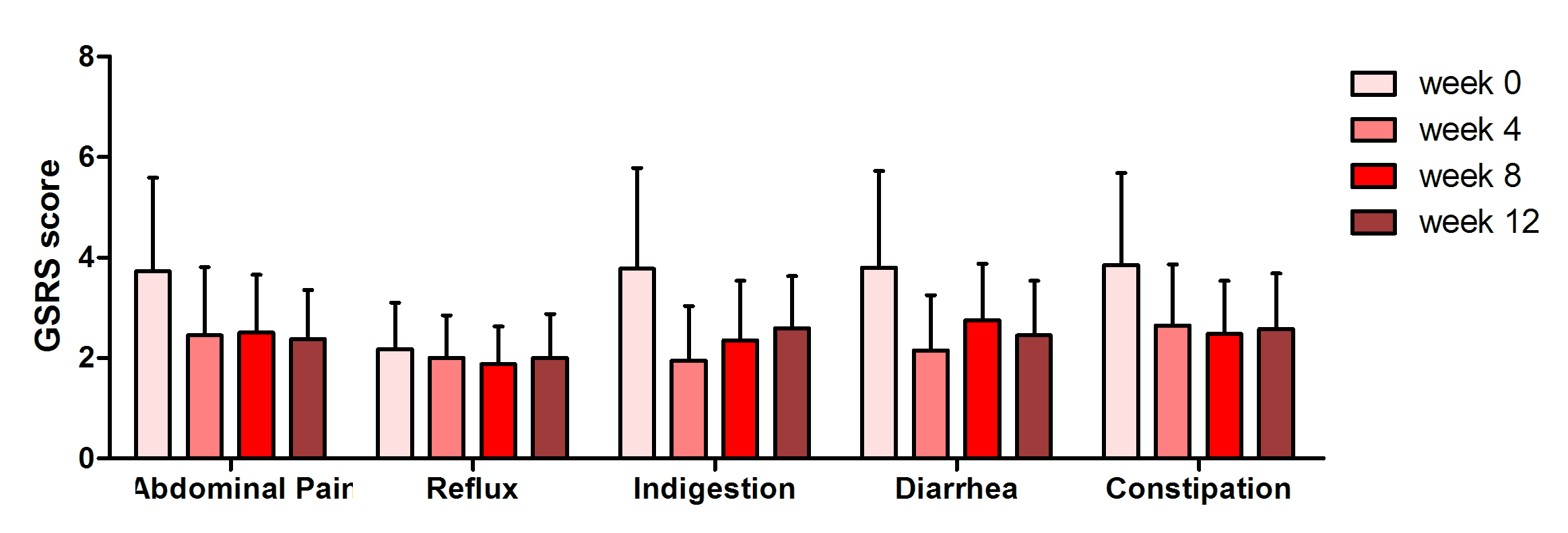

Supplement: Supplementary file 1 [file DataSheet_1.zip › raw data/Figure 2/GSRS/GSRS subgroup.tif]

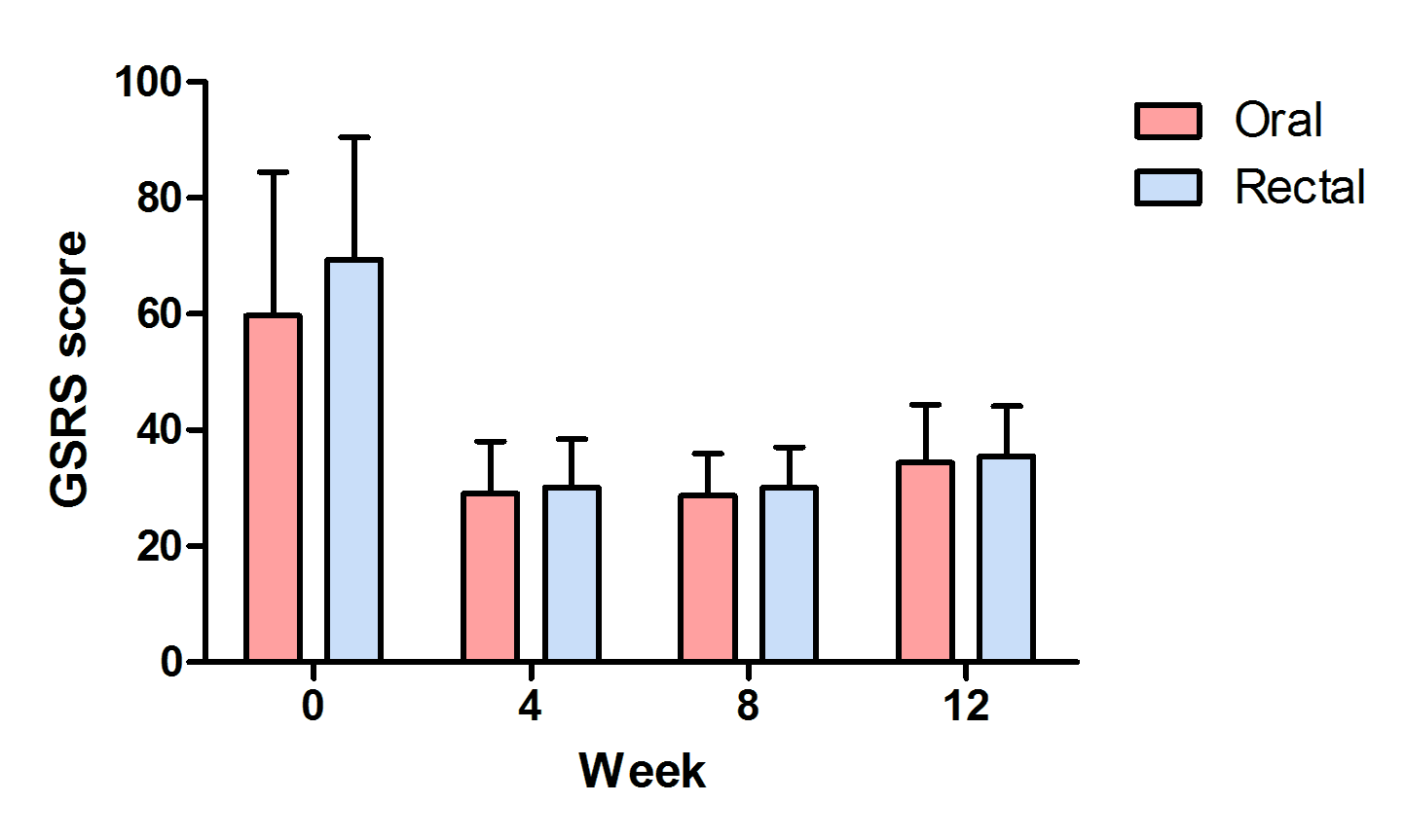

Supplement: Supplementary file 1 [file DataSheet_1.zip › raw data/Figure 2/GSRS/GSRS.tif]

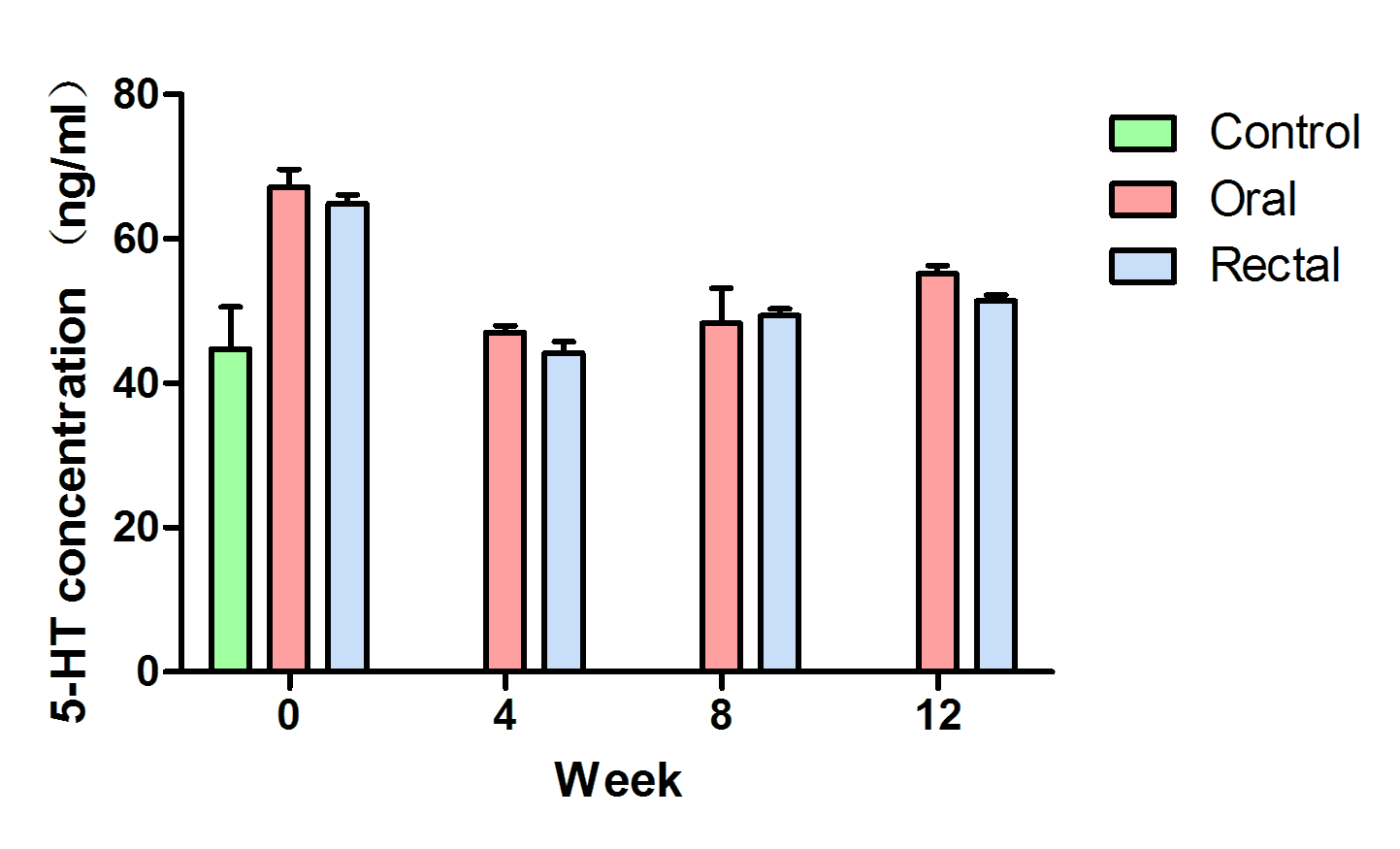

Supplement: Supplementary file 1 [file DataSheet_1.zip › raw data/Figure 3/5-HT/5-HT.tif]

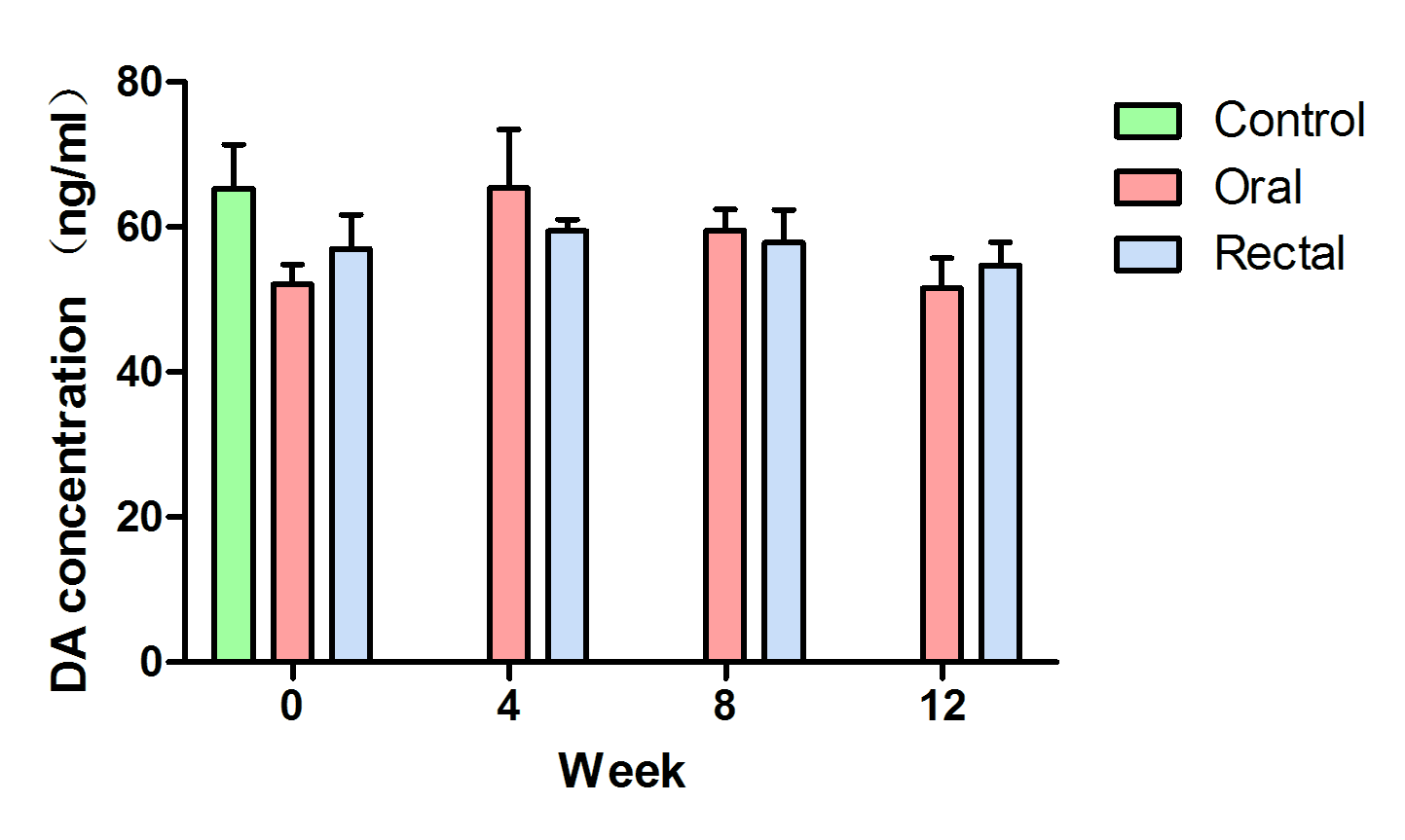

Supplement: Supplementary file 1 [file DataSheet_1.zip › raw data/Figure 3/DA/DA.tif]

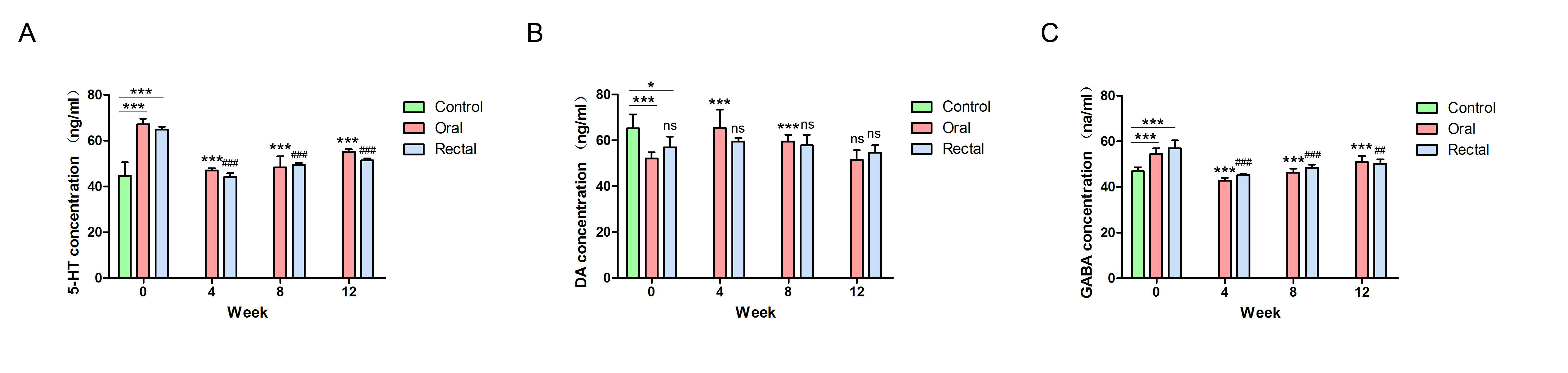

Supplement: Supplementary file 1 [file DataSheet_1.zip › raw data/Figure 3/figure 3.jpg]

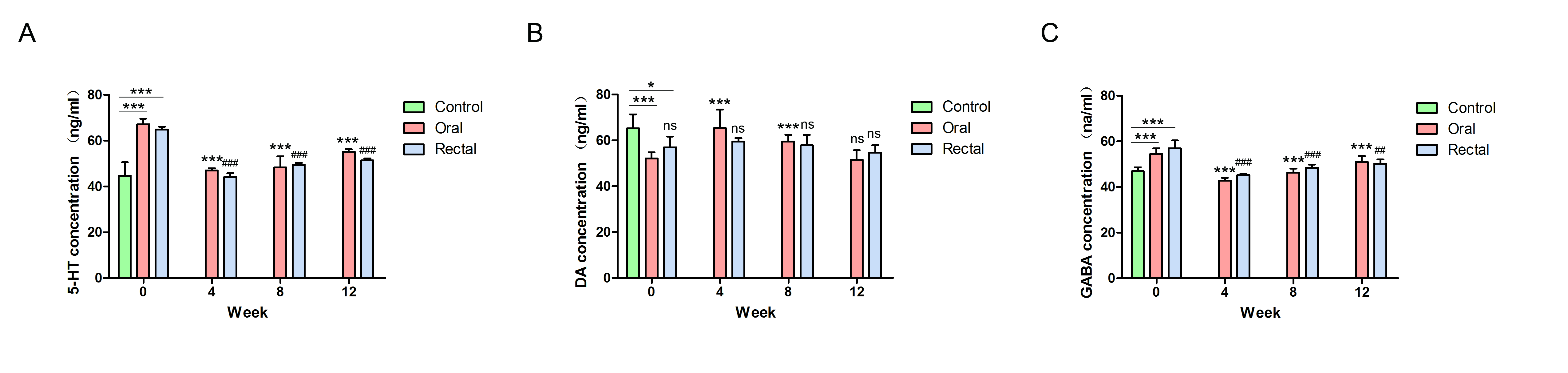

Supplement: Supplementary file 1 [file DataSheet_1.zip › raw data/Figure 3/figure 3.tif]

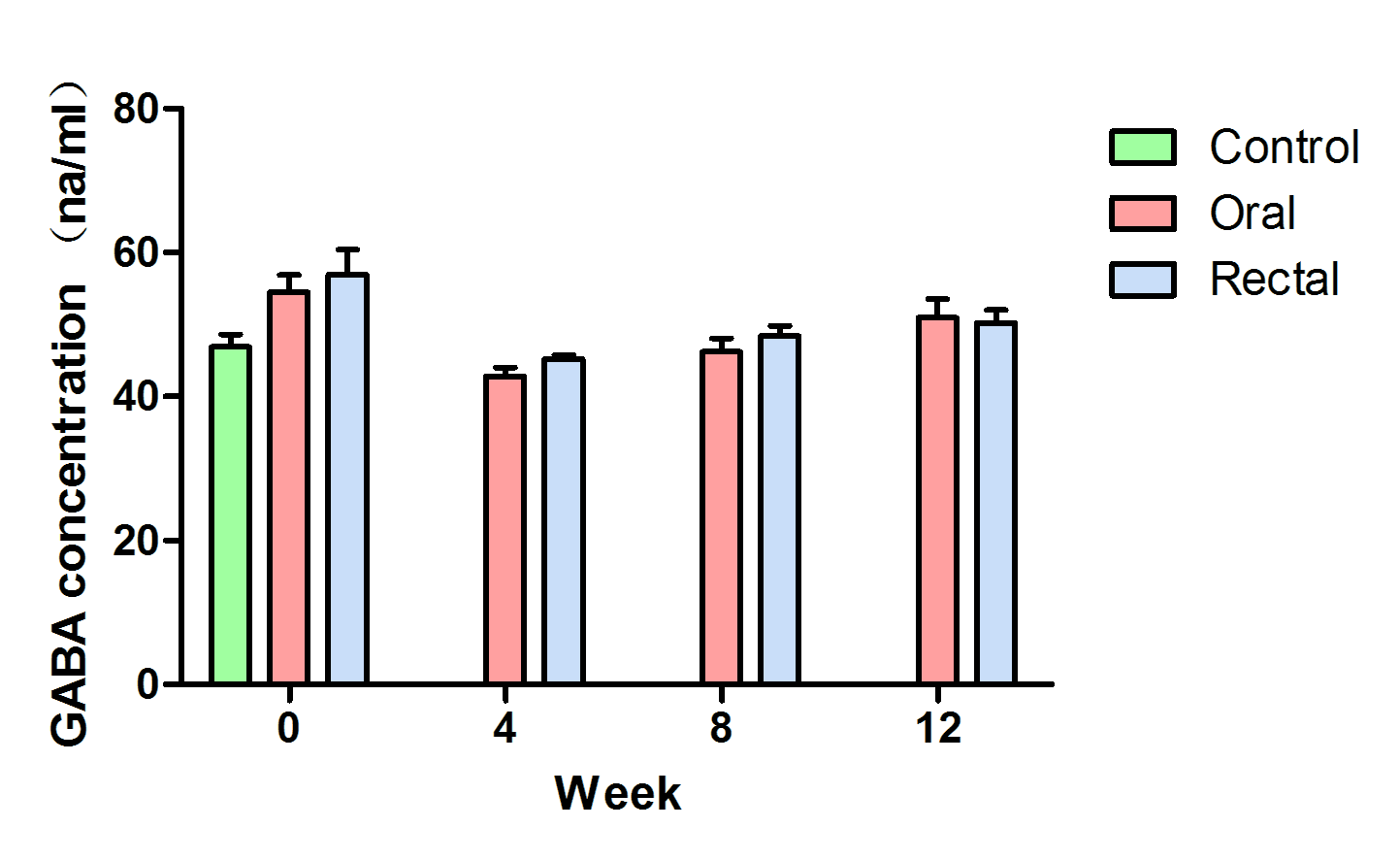

Supplement: Supplementary file 1 [file DataSheet_1.zip › raw data/Figure 3/GABA/GABA.tif]

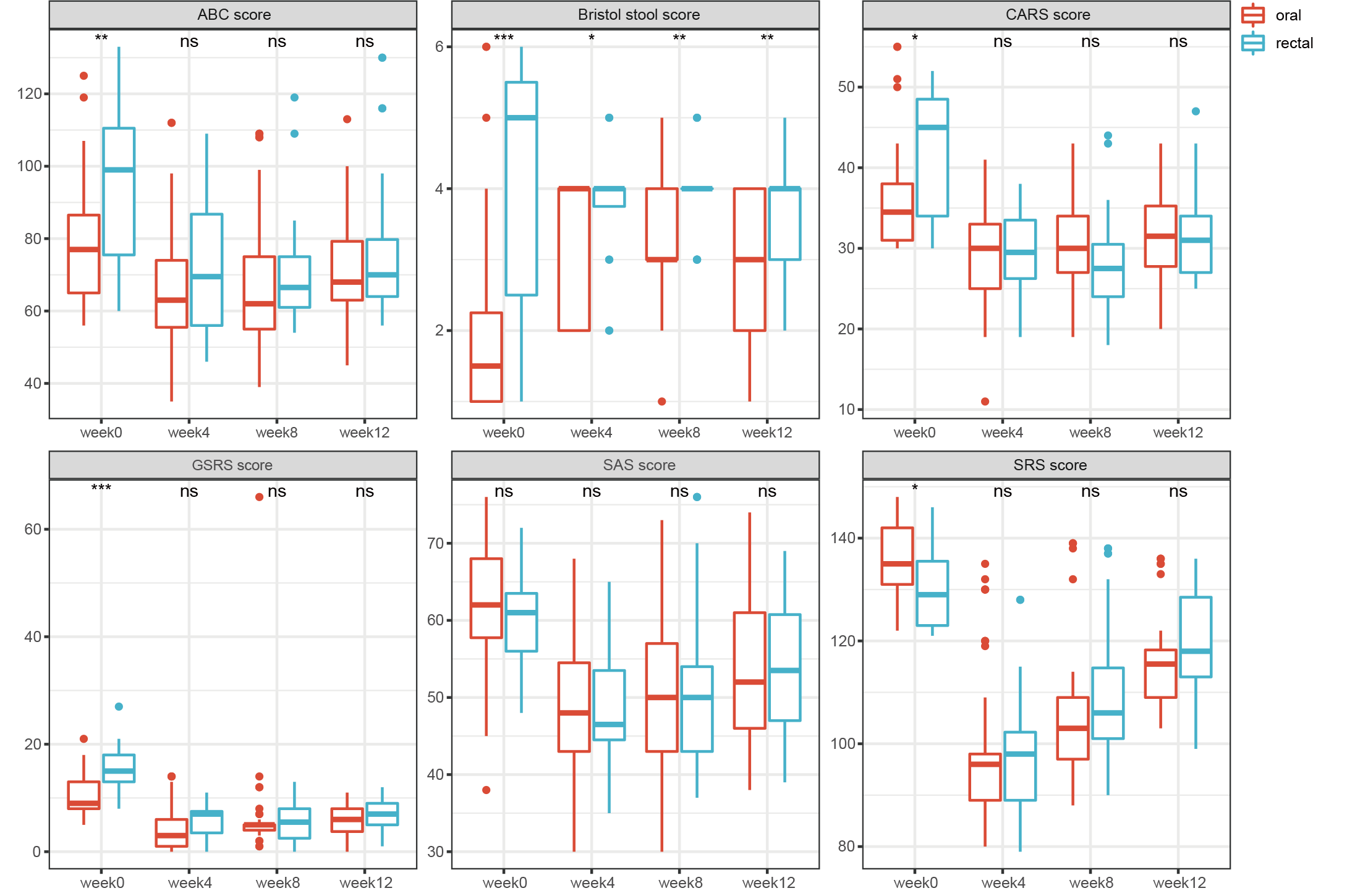

Supplement: Supplementary file 2 [file Image_1.tif]

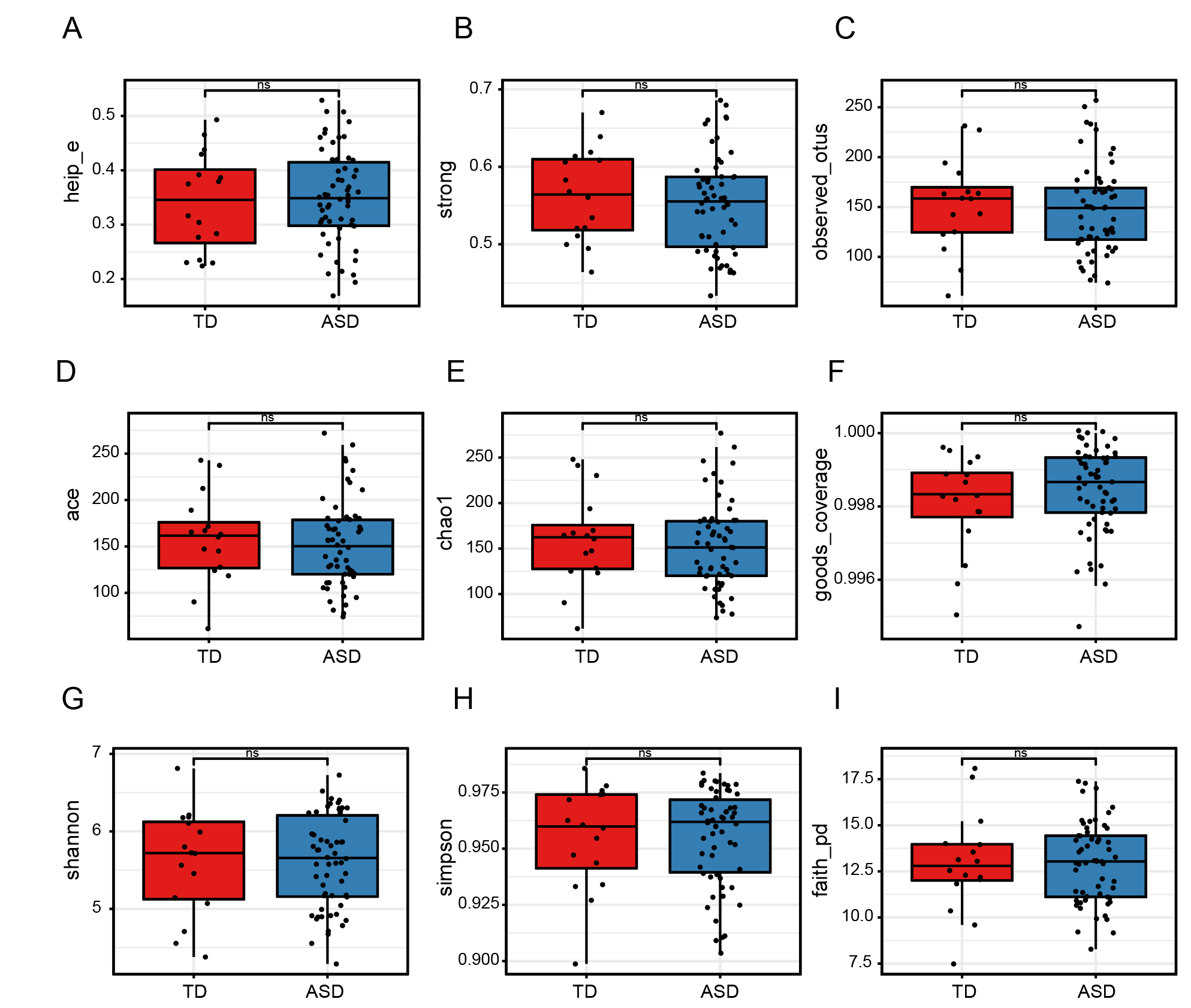

Supplement: Supplementary file 3 [file Image_2.tif]

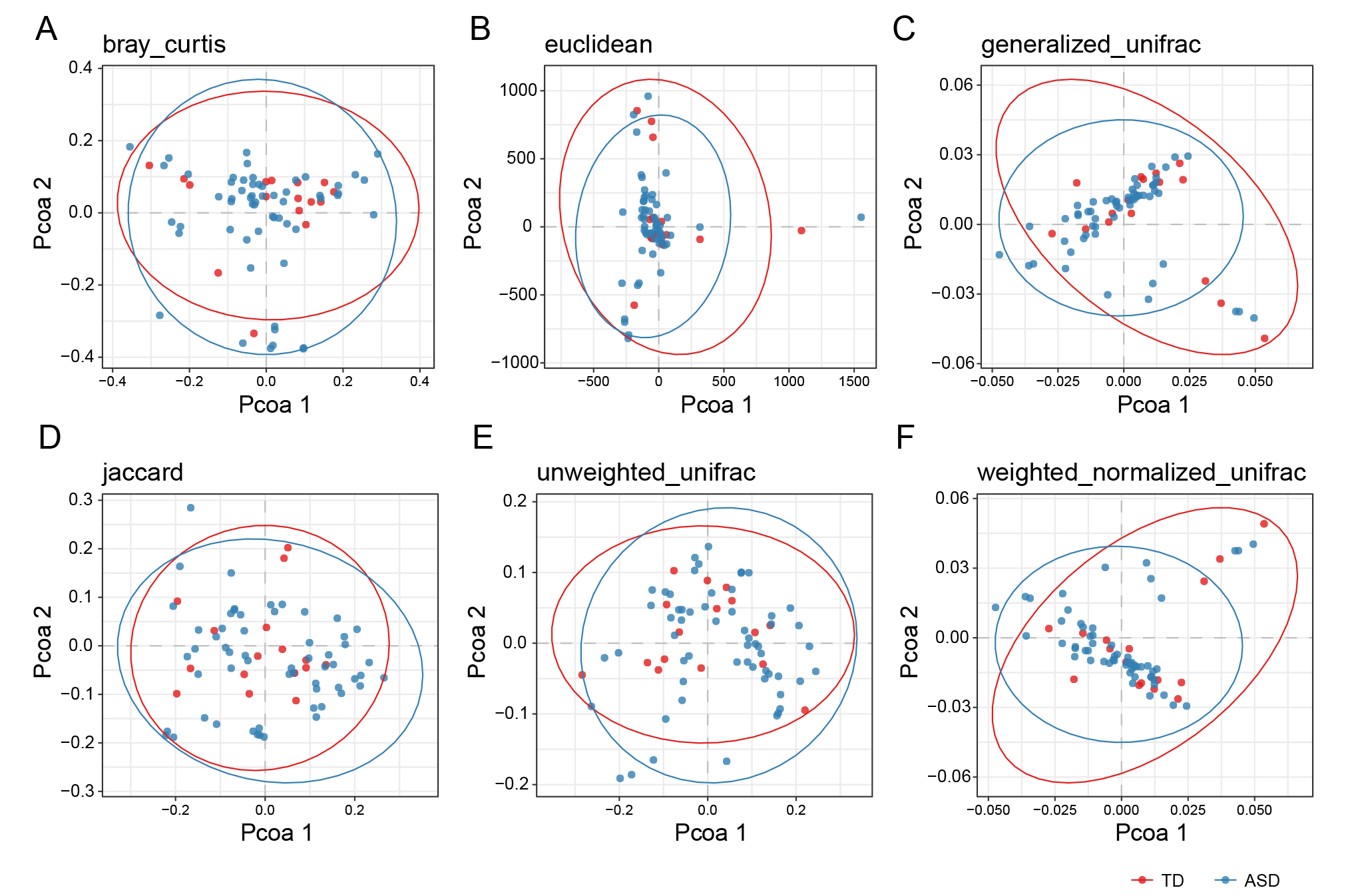

Supplement: Supplementary file 4 [file Image_3.tif]

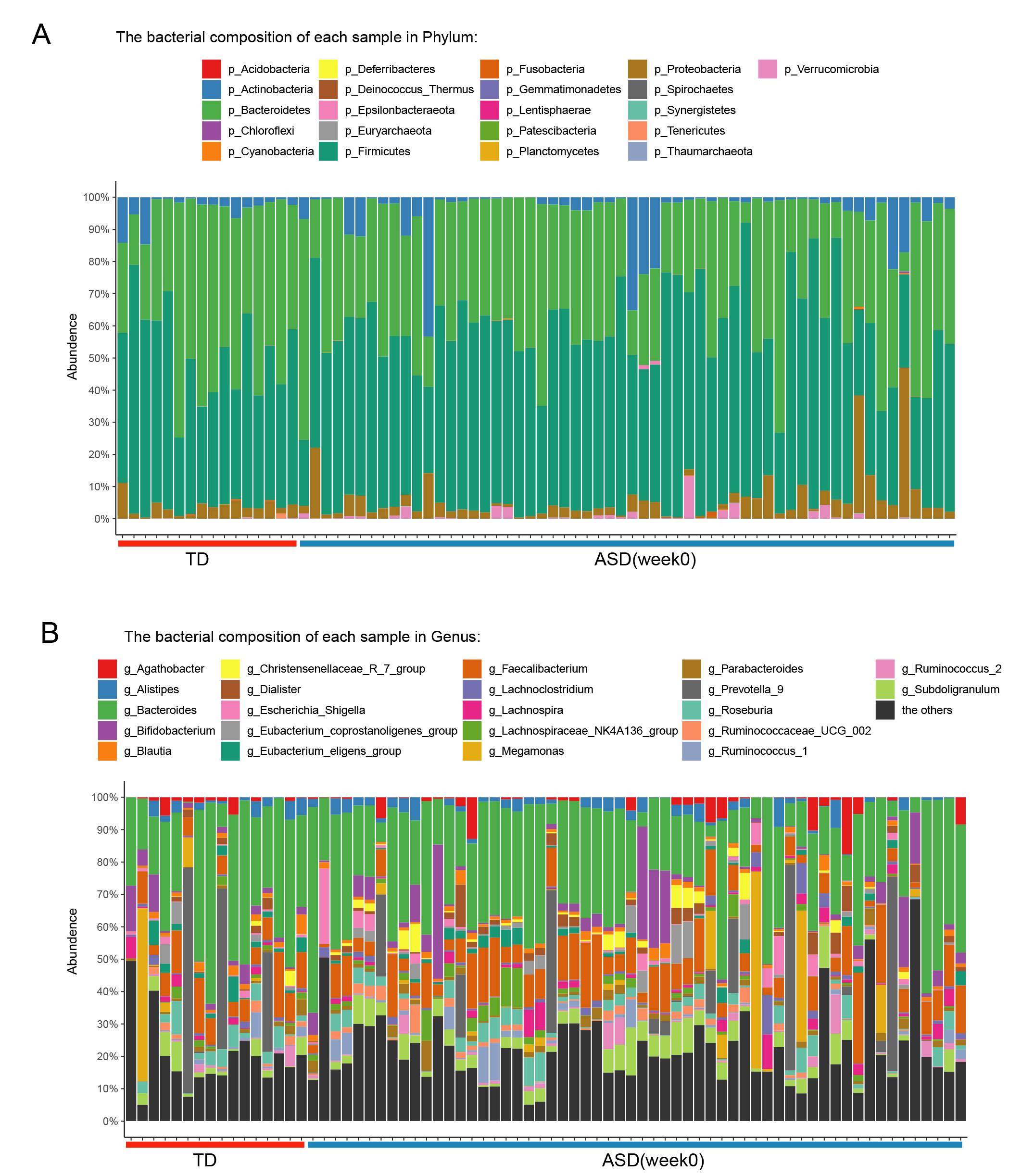

Supplement: Supplementary file 5 [file Image_4.jpg]

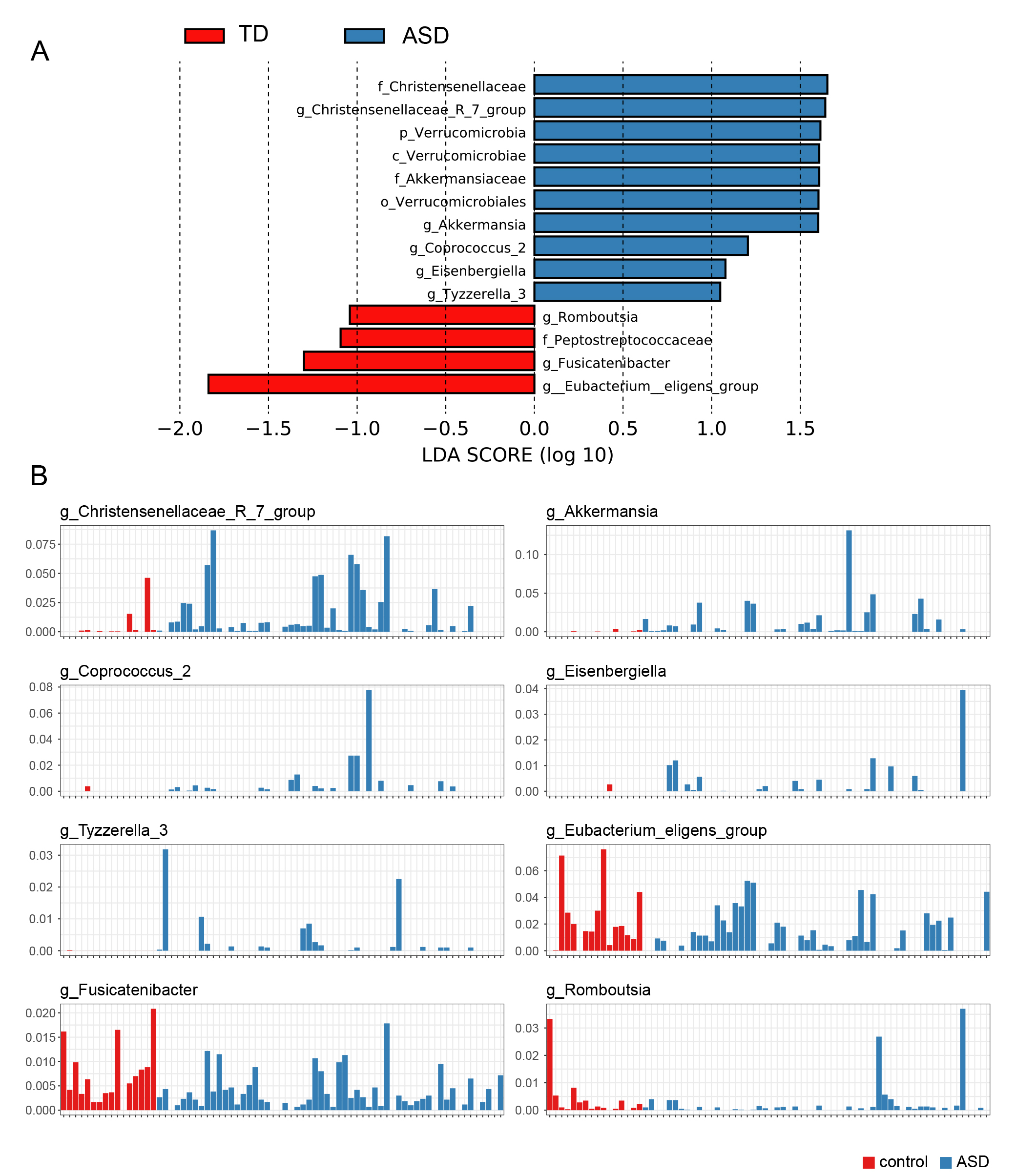

Supplement: Supplementary file 6 [file Image_5.jpg]

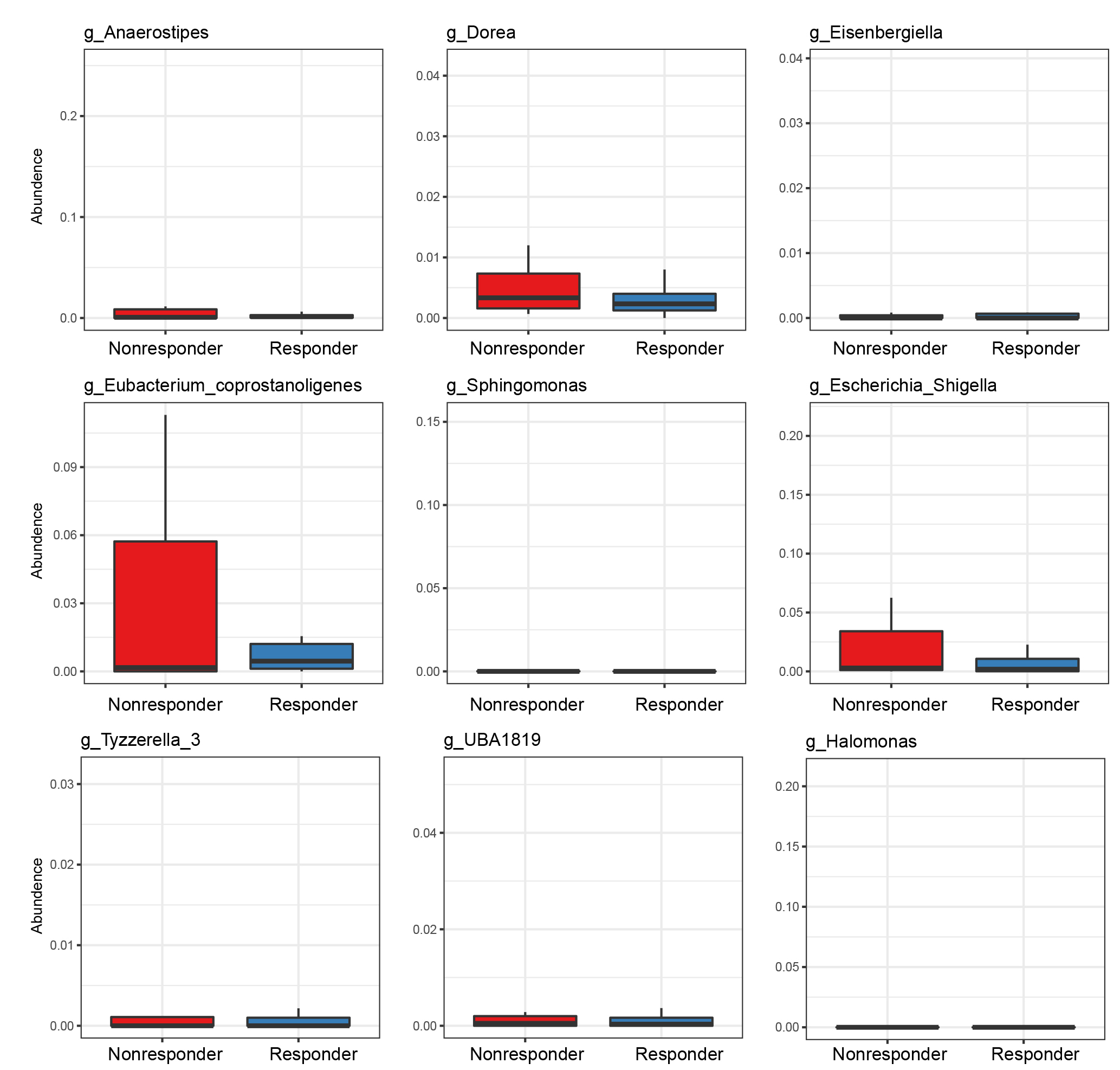

Supplement: Supplementary file 7 [file Image_6.jpg]
