# Supplementary material for: Fecal Microbiota Transplantation Relieves Gastrointestinal and Autism Symptoms by Improving the Gut Microbiota in an Open-Label Study
Source: Front Cell Infect Microbiol. 2021 Oct 19;11:759435. doi: 10.3389/fcimb.2021.759435 (PMC8560686; doi:10.3389/fcimb.2021.759435)
Supplement: Supplementary file 8 [file Table_1.docx]

**Supplementary table 1. Correlation between serum neurotransmitters and clinical outcomes of ASD participants after FMT treatment.**

| Neurotransmitters | Clinical outcomes | R | P |
| --- | --- | --- | --- |
| 5-HT | GSRS | -0.152 | 0.295 |
| 5-HT | Bristol stool score | -0.434 | 0.001 |
| 5-HT | CARS | 0.016 | 0.911 |
| 5-HT | ABC | -0.068 | 0.637 |
| 5-HT | SRS | -0.2036 | 0.160575 |
| 5-HT | SAS | 0.072 | 0.618 |
| DA | GSRS | 0.115 | 0.461 |
| DA | Bristol stool score | -0.110 | 0.482 |
| DA | CARS | 0.215 | 0.164 |
| DA | ABC | -0.024 | 0.876 |
| DA | SRS | -0.047 | 0.764 |
| DA | SAS | 0.048 | 0.755 |
| GABA | GSRS | 0.022 | 0.876 |
| GABA | Bristol stool score | 0.527 | P < 0.001 |
| GABA | CARS | -0.086 | 0.555 |
| GABA | ABC | 0.021 | 0.885 |
| GABA | SRS | 0.068 | 0.638 |
| GABA | SAS | -0.127 | 0.380 |
